# High-resolution imaging and manipulation of endogenous AMPA receptor surface mobility during synaptic plasticity and learning

**DOI:** 10.1126/sciadv.abm5298

**Published:** 2022-07-27

**Authors:** Angela M. Getz, Mathieu Ducros, Christelle Breillat, Aurélie Lampin-Saint-Amaux, Sophie Daburon, Urielle François, Agata Nowacka, Mónica Fernández-Monreal, Eric Hosy, Frédéric Lanore, Hanna L. Zieger, Matthieu Sainlos, Yann Humeau, Daniel Choquet

**Affiliations:** ^1^ Université de Bordeaux, CNRS, Interdisciplinary Institute for Neuroscience (IINS), UMR 5297, F-33000 Bordeaux, France.; ^2^ Université de Bordeaux, CNRS, INSERM, Bordeaux Imaging Center (BIC), UAR 3420, US 4, F-33000 Bordeaux, France.

## Abstract

Regulation of synaptic neurotransmitter receptor content is a fundamental mechanism for tuning synaptic efficacy during experience-dependent plasticity and behavioral adaptation. However, experimental approaches to track and modify receptor movements in integrated experimental systems are limited. Exploiting AMPA-type glutamate receptors (AMPARs) as a model, we generated a knock-in mouse expressing the biotin acceptor peptide (AP) tag on the GluA2 extracellular N-terminal. Cell-specific introduction of biotin ligase allows the use of monovalent or tetravalent avidin variants to respectively monitor or manipulate the surface mobility of endogenous AMPAR containing biotinylated AP–GluA2 in neuronal subsets. AMPAR immobilization precluded the expression of long-term potentiation and formation of contextual fear memory, allowing target-specific control of the expression of synaptic plasticity and animal behavior. The AP tag knock-in model offers unprecedented access to resolve and control the spatiotemporal dynamics of endogenous receptors, and opens new avenues to study the molecular mechanisms of synaptic plasticity and learning.

## Teaser

We measure and control receptor surface traffic in intact tissue with a versatile knock-in mouse model and molecular toolkit.

## INTRODUCTION

Changes in synaptic transmission efficacy and network connectivity, particularly in the context of experience-dependent synaptic plasticity, are at the core of behavioral adaptation, learning, and memory (*1*, *2*). This has brought to center stage the need to understand the cellular and molecular mechanisms that modulate synaptic function. Among the many pre- and postsynaptic mechanisms that control synaptic gain, regulation of the number, type, and nanoscale organization of neurotransmitter receptors at the postsynaptic density (PSD) has emerged as a principal determinant of the amplitude of both excitatory and inhibitory synaptic responses (*3*, *4*). This is established by a complex interplay between a series of trafficking processes, including intracellular transport, exo- and endocytosis, lateral diffusion on the cell surface, and reversible stabilization at the PSD through interactions with scaffolding elements. Together, this results in a dynamic equilibrium of receptors distributed among different subcellular compartments that ultimately sets their number in front of transmitter release sites.

Molecular replacement strategies or overexpression approaches introducing modified receptor subunits [e.g., chimeras, truncations, and super-ecliptic pHluorin (SEP) tags] have been widely used to study the contributions of receptor trafficking mechanisms to synaptic plasticity (*5*–*10*). Notwithstanding their extensive use, these approaches face a number of experimental limitations, including disrupting the set point of the “diffusion-trapping” equilibrium through changes in receptor subunit composition, synaptic targeting, alterations of the receptor surface pool, and saturation of synaptic anchoring sites. These off-target effects are likely to produce measurement artifacts while allowing only a handful of neurons in a circuit to be assayed at a given time. This makes them of limited use for addressing experimental questions at the level of integrated neuronal circuits under endogenous conditions, or with behavioral paradigms in vivo. Strategies to label endogenous receptors through either the creation of knock-in (KI) mouse lines with fluorescently tagged subunits (*11*) or chemical labeling of native receptors (*12*, *13*) have recently been developed, and avoid artifacts linked to receptor overexpression. However, important limitations remain to be overcome, including the presence of bulky extracellular tags that may impede normal receptor function or synaptic access (*14*), or the ability to specifically label distinct cell subsets within dense tissues for high-resolution imaging.

An important feature of the control of receptor numbers at synapses is that sites of receptor endo- and exocytosis are primarily extrasynaptic (*15*–*17*). Consequently, the primary pathway for the addition or removal of synaptic receptors is through surface movements powered by Brownian diffusion (*18*–*20*). Controlling receptor surface diffusion has emerged as a powerful avenue toward artificial regulation of synaptic plasticity by preventing variations in receptor numbers at synapses. The use of antibodies against extracellular domains of endogenous receptor subunits [e.g., γ-aminobutyric acid type A (GABA_A_), *N*-methyl-d-aspartate (NMDA), α-amino-3-hydroxy-5-methyl-4-isoxazolepropionic acid (AMPA), and kainate receptors] has proven an effective method to control their movements on the neuronal surface through the cross-linking effects introduced by the divalent binding domains of antibodies (*21*–*25*). Extracellular cross-linkers provide a specific tool to manipulate receptor mobility without altering basal synaptic transmission or circuit function. These approaches have established the pivotal role of AMPA receptor (AMPAR) surface trafficking in short-term and canonical long-term plasticity (LTP), as well as the link between variations in synaptic receptor numbers and higher brain functions such as cued fear learning and whisker-dependent somatosensory behavior (*23*, *25*–*27*). The ability to visualize and control receptor fluxes during plasticity could potentially allow researchers to study the precise timing and contributions of changes in synaptic receptor content during various higher brain functions such as working memory or memory engram formation. However, progress in this area has been hindered by the lack of appropriate experimental tools that can specifically monitor or manipulate receptor dynamics at the level of single synaptic contacts or particular subclasses of neurons within a circuit.

There is therefore a great need to develop new molecular tools that will allow researchers to overcome the aforementioned experimental limitations. An ideal approach would be one that allows target-specific monitoring and manipulation of endogenous receptor surface mobility dynamics, with a resolution that can be tuned to the level of single molecules, individual synapses, or integrated synaptic networks, while preserving endogenous receptor composition and function. The avidin-biotin system for labeling cell surface proteins has recently emerged as a promising candidate for such an approach (*28*, *29*). Proteins tagged with the 15–amino acid biotin acceptor peptide (AP) sequence are selectively biotinylated in the endoplasmic reticulum (ER) upon the expression of biotin ligase (BirA) from *Escherichia coli* modified to contain an ER retention sequence (BirA^ER^) or on the cell surface upon the addition of soluble recombinant BirA (sBirA). Biotinylated proteins on the cell surface are recognized by the avidin family of small, high-affinity biotin binding proteins, including StreptAvidin and NeutrAvidin (SA/NA; ~6 nm), or monomeric SA (mSA; ~3 nm), which can efficiently penetrate dense tissues (*30*).

A number of features make the avidin-biotin system particularly well suited for studying the surface mobility dynamics and nanoscale organization of synaptic proteins in integrated systems. First, distinct molecular strategies to introduce BirA allow time-controlled and target-specific AP tag biotinylation and avidin recognition in a constitutive KI model where all neurons express the AP-tagged protein. This approach uniquely allows endogenous receptor labeling of sparse neurons in tissue, a feature that is particularly important for high-resolution imaging. Second, addition of the small AP tag sequence is unlikely to have a substantial impact on protein expression, structure, function, or trafficking. Third, the small size and high affinity of biotin binding proteins offer enhanced synaptic access, labeling stability, and signal intensity over antibody- or fluorescent protein conjugate–based approaches. Fourth, because of the availability of monovalent (mSA) and tetravalent (NA and SA) variants of biotin binding proteins, AP tagging presents a molecular strategy that can be used either to monitor the surface mobility dynamics and nanoscale organization of individual proteins or to efficiently manipulate surface diffusion dynamics by cross-linking–dependent immobilization (*25*, *31*).

Here, we report the development and characterization of an AP-GluA2 KI mouse model, where *Gria2* was modified by CRISPR-Cas9 genome editing to introduce the AP tag to the extracellular N-terminal of the GluA2 AMPAR subunit. We chose GluA2 as a primary target for this proof-of-principle study, as most AMPARs in hippocampal pyramidal neurons are composed of GluA2/GluA1 heteromers, with little, if any, GluA1 homomers expressed on the cell surface at the basal level (*32*). The expression, localization, and function of AP-GluA2 were not affected by the modification, and the synaptic physiology and behavioral phenotyping of KI animals were indistinguishable from wild type (WT). We developed in parallel a molecular toolkit that allows the biotinylation of AP-GluA2 to be tailored to a variety of spatiotemporal resolutions and experimental preparations, ranging from superresolution imaging of single molecules to manipulating integrated circuits in behaving animals. Development of a custom lattice light sheet microscope (LLSM) with a photostimulation module (PSM) enabled us to perform fluorescence recovery after photobleaching (FRAP) imaging of biotinylated AP-GluA2 (bAP-GluA2) with monovalent or tetravalent avidin probes to measure the native surface mobility and cross-linking–dependent immobilization of endogenous AMPAR in brain slices. This module also allowed us to perform simultaneous LLSM imaging and two-photon glutamate uncaging, demonstrating that our combination of LLSM and PSM technology allows all-optical physiology experiments in live tissue preparations. In acute slices, NA cross-linking of bAP-GluA2–containing AMPAR blocked the expression of LTP at Schaffer collaterals, and delivery of NA into the CA1 region in vivo blocked the formation of contextual fear memory. This experimental model affords unparalleled resolution and control over the spatiotemporal dynamics of endogenous AMPAR for integrated physiological studies.

## RESULTS

### Target-specific detection of surface bAP-GluA2 in organized brain tissue

**Fig. 1.**
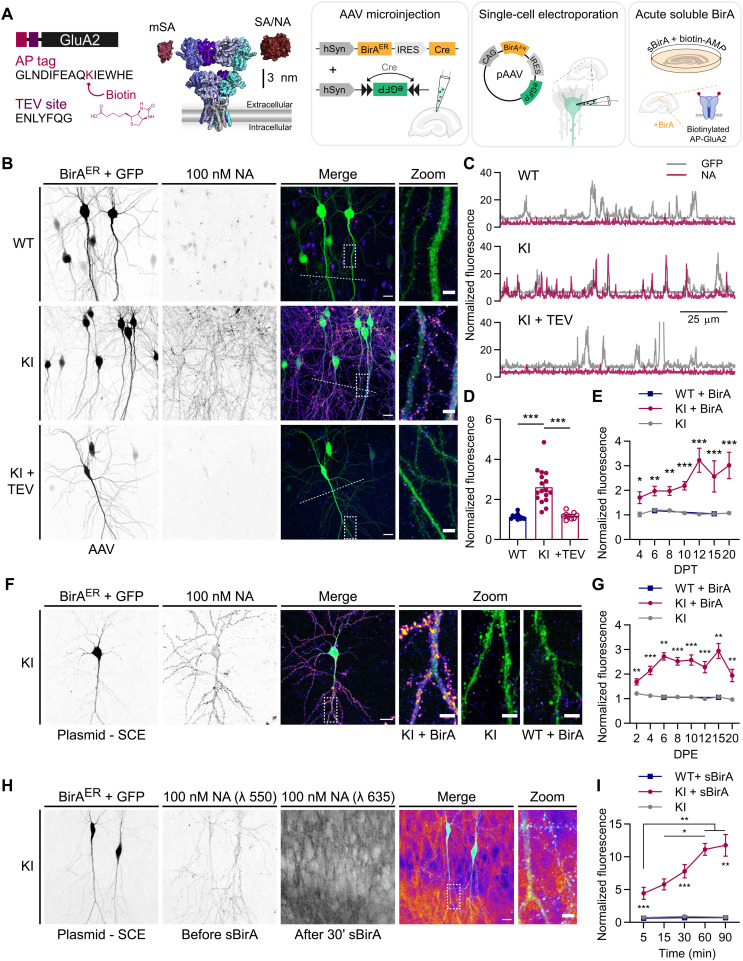
Target-specific surface labeling of AMPAR by biotin binding proteins in an AP-GluA2 KI model. (**A**) Schematic representation of the AP-GluA2 KI-modified extracellular N-terminal containing an AP tag and TEV protease consensus sequence, with a scaled model of AMPAR with monovalent (mSA) or tetravalent (SA and NA) biotin binding proteins (left) and molecular strategies to biotinylate AP-GluA2 (right). (**B**) Representative confocal images of CA1 pyramidal neurons in WT or AP-GluA2 KI slice cultures transduced with BirA^ER^-Cre + FLEx eGFP AAV, incubated with NA–DyLight 633 to label surface bAP-GluA2 (top and middle) and TEV protease to cleave the AP tag and remove the NA surface label (bottom). (**C**) Line scans [dashed lines in (B)] reveal extent of NA colocalization with eGFP reporter. (**D**) Normalized fluorescence intensity of NA–DyLight 633, coincident with eGFP reporter. *N* ≥ 9. ***P ≤ 0.0001 [Welch’s analysis of variance (ANOVA), *F* = 23.99, *P* < 0.0001; Dunnett’s post hoc test]. (**E**) Time course of bAP-GluA2 expression in KI slices transduced with BirA^ER^-Cre + FLEx eGFP AAV at 1 DIV, relative to WT (+BirA) or Cre control (−BirA). DPT, days post-transduction. *N* ≥ 3. *P ≤ 0.0201, **P ≤ 0.0089, and ***P ≤ 0.0009 (Mann-Whitney *U* test, unpaired *t* test, or Kruskal-Wallis test; *F* ≥ 12.44, *P* ≤ 0.002, Dunn’s post hoc test). (**F**) CA1 pyramidal neurons in AP-GluA2 KI or WT slices electroporated with BirA^ER^-eGFP or eGFP control plasmids at 3 DIV. (**G**) Time course of bAP-GluA2 expression in KI slices electroporated with BirA^ER^-eGFP, relative to WT (+BirA) or eGFP control (−BirA). DPE, days post-electroporation. *N* ≥ 5. **P ≤ 0.0098 and ***P ≤ 0.0005 (Mann-Whitney *U* test, unpaired *t* test, Kruskal-Wallis test, or Welch’s ANOVA; *F* ≥ 16.22, *P* ≤ 0.0006, Dunn’s or Dunnett’s post hoc test). (**H**) Two-color labeling of BirA^ER^-eGFP electroporated KI slices, first with NA–DyLight 550 and then with NA-STAR 635P after 30-min incubation with sBirA, which rapidly biotinylates surface AP-GluA2. (**I**) Time course of bAP-GluA2 expression in sBirA-incubated KI slices, relative to WT or biotin-AMP control. *N* ≥ 5. *P ≤ 0.042, **P ≤ 0.0051, and ***P ≤ 0.0007 (Kruskal-Wallis test or Welch’s ANOVA; *F* ≥ 14.69, *P* ≤ 0.0003, Dunn’s or Dunnett’s post hoc test). Scale bars, 20 and 5 μm. Error bars, SEM. See also figs. S1 to S8.

We used CRISPR-Cas9 genome editing of *Gria2* to introduce the biotin AP sequence (GLNDIFEAQKIEWHE) and a downstream tobacco etch virus (TEV) protease consensus sequence (ENLYFQG) onto the GluA2 N-terminal (Fig. 1A and fig. S1). The AP tag enables endogenous GluA2-containing AMPAR to be labeled with avidin probes upon enzymatic biotinylation by BirA, while the TEV site allows the KI sequence to be enzymatically removed for reversal of surface labeling and cross-linking. To achieve target-specific biotinylation of AP-GluA2, we developed a variety of molecular approaches via the chronic expression of ER-resident BirA (BirA^ER^) by adeno-associated virus (AAV) transduction or single-cell electroporation (SCE), or the acute application of sBirA (Fig. 1A). For applications requiring sparse labeling, we developed a two-component AAV viral system, with one virus encoding BirA^ER^ with Cre recombinase following an internal ribosomal entry site (IRES) (BirA^ER^-Cre), used at a low concentration with a second virus encoding a floxed enhanced green fluorescent protein (eGFP) reporter. We also used an alternative plasmid system encoding BirA^ER^ IRES eGFP (BirA^ER^-eGFP) (see Methods). The design of these constructs allows the reporter to be easily exchanged, thereby facilitating coupling of the avidin-biotin system to various functional assays with fluorescent reporters. We used AAV microinjection or SCE of CA1 pyramidal neurons in organotypic hippocampal slices cultured with 10 μM biotin supplementation and performed live labeling with dye-conjugated NA to detect surface bAP-GluA2. For controls, we mirrored the above conditions in WT slices, or in AP-GluA2 KI slices without BirA^ER^, using an AAV encoding Cre alone or a plasmid encoding IRES eGFP. NA labeling was specific to bAP-GluA2 in KI slices with BirA^ER^ and was well correlated with the eGFP reporter (Fig. 1, B to G). TEV incubation efficiently removed the extracellular NA label within 10 min (Fig. 1, B to D, and fig. S2). To define the experimental time frame for this system, we monitored the expression of bAP-GluA2 over time in culture and observed saturation after 12 days in vitro (DIV) with AAV-mediated expression of BirA^ER^ (Fig. 1E and fig. S3) and after 6 DIV with plasmid-mediated expression of BirA^ER^ (Fig. 1G and fig. S4). We found no differences in CA1 pyramidal neuron spine density between WT and KI organotypic slices expressing BirA^ER^-eGFP or eGFP control plasmids, suggesting that both the KI mutation and BirA^ER^ expression are well tolerated by neurons (fig. S5).

For bulk labeling applications, we developed an acute approach using incubation with sBirA and biotin–AMP (adenosine 5′-monophosphate), where detection of bAP-GluA2 saturated after 60 min (Fig. 1, H and I, and fig. S6), and a chronic approach with an AAV encoding BirA^ER^-eGFP, where essentially all neurons within the infection zone expressed bAP-GluA2 (fig. S7). We found that serial dilution of the BirA^ER^-eGFP AAV similarly allows sparse labeling of neurons in preparations from KI mice; however, with this construct, biotinylation of AP-GluA2 is often detectable where the expression of the eGFP reporter is below the observable limit (figs. S7 and S8). This highlights the high enzymatic efficiency of BirA^ER^, the limited utility of low-titer single-virus strategies for imaging applications with the AP tag KI system (fig. S8), and the advantage brought by the BirA^ER^-Cre construct described above (Fig. 1, B and C, and fig. S3). Note that this system is amenable to the characterization of distinct populations of surface AMPAR using two-color avidin labeling before and after an experimental stimulus. Here, for example, we used differentially dye-coupled NA to identify surface bAP-GluA2 before and after acute biotinylation by sBirA (Fig. 1H). The long-term BirA^ER^ gene delivery strategies and the rapid acute sBirA approaches therefore offer access to two distinct temporal windows, making the AP-GluA2 KI model amenable to a variety of experimental approaches. Together, these results demonstrate the high degree of specificity and flexibility of this system for labeling endogenous AMPAR in organized brain tissue.

### Monitoring and manipulating endogenous AMPAR surface mobility

**Fig. 2.**
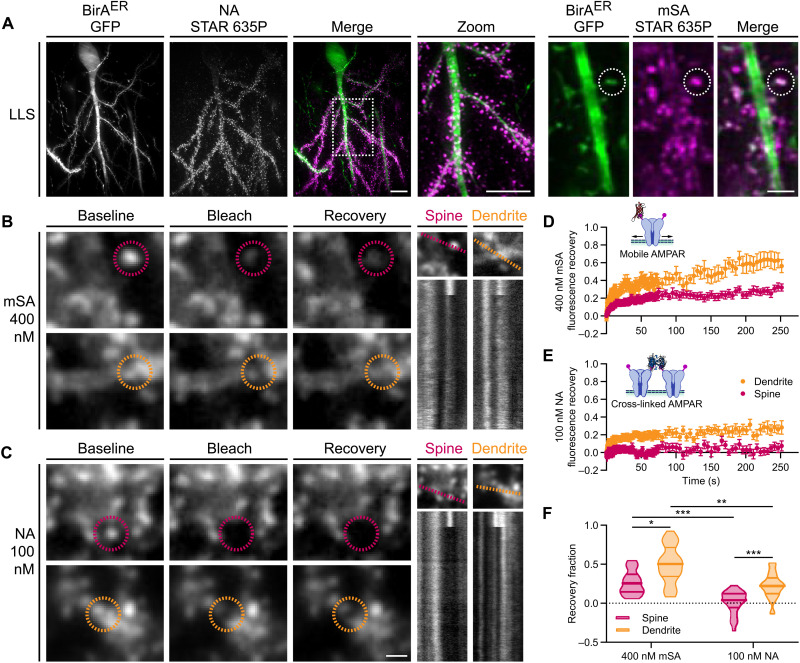
LLSM-FRAP with biotin binding proteins reveals surface mobility and immobilization of AP-GluA2–containing AMPAR. (**A**) Representative three-dimensional (3D) reconstructed LLSM images of CA1 pyramidal neurons in AP-GluA2 KI slice cultures transduced with BirA^ER^-Cre + FLEx eGFP or BirA^ER^-eGFP AAV, incubated with NA (left) or mSA (right) to label surface bAP-GluA2. Scale bars, 10 and 2 μm. (**B** and **C**) FRAP experiments performed on a custom LLSM with PSM in AP-GluA2 KI slices labeled with mSA-STAR 635P (B) or NA-STAR 635P (C). Representative images show spine or dendrite ROIs (dashed circles) before (baseline; −1 s) and after targeted photobleaching (bleach; +0.5 s) and diffusion-dependent recovery (recovery; +70 s). Scale bar, 1 μm. Kymographs illustrate fluorescence recovery profiles of the targeted ROIs during the acquisition (dashed line; ~250 s). (**D** and **E**) Normalized mean fluorescence recovery curves for bAP-GluA2 labeled with monovalent mSA reveal synaptic and extrasynaptic mobility of endogenous AMPAR (D) or tetravalent NA, which immobilizes surface AMPAR by cross-linking of bAP-GluA2 (E). *N* ≥ 25. In total, 52 to 88% of FRAP ROIs were maintained in the LLSM excitation plane and followed for the full length of the acquisition. Error bars, SEM. (**F**) Quantification of the AMPAR mobile fraction by curve fitting of individual FRAP recovery profiles; plot shows median with first and third quartiles. *N* ≥ 25. *P ≤ 0.0333, **P ≤ 0.0026, and ***P ≤ 0.0005 (Kruskal-Wallis test; *F* = 53.60, *P* < 0.0001; Dunn’s post hoc test). See also figs. S9 to S12.

The small size of biotin binding proteins affords enhanced labeling accessibility for confined environments such as in organized tissue or at the synapse (*31*). However, live high-resolution imaging of endogenous protein dynamics in optically scattering tissue preparations represents a considerable challenge. To circumvent photobleaching, phototoxicity, and optical scattering constraints, we developed an LLSM with a PSM to determine the mobility of bAP-GluA2 by measuring the rate of FRAP (fig. S9). In BirA^ER^-eGFP– or BirA^ER^-Cre + eGFP–transduced KI organotypic slices, we labeled surface bAP-GluA2 with 400 nM mSA or 100 nM NA conjugated to the fluorophore STAR 635P (Fig. 2A). Regions of interest (ROIs) on CA1 pyramidal neuron spines and dendrites in the stratum radiatum were photobleached, and fluorescence recovery was followed for ~250 s to measure the surface diffusion of synaptic and extrasynaptic AMPAR. We used curve fitting of FRAP profiles to determine the recovery fraction as an estimate of the mobile AMPAR population. Using mSA, we found that the recovery fraction was 0.27 ± 0.03 at spines and 0.50 ± 0.05 on dendrites. With NA, the recovery fraction was reduced to 0.01 ± 0.03 at spines and 0.22 ± 0.03 on dendrites (Fig. 2, B to F). The density of bAP-GluA2 and AMPAR mobility, measured as initial ROI intensity of mSA or NA labeling versus FRAP recovery fraction, was not significantly correlated (fig. S10). Together, these observations indicate that (i) mSA labeling of bAP-GluA2 affords access to monitoring the diffusion dynamics of endogenous AMPAR in organized brain tissue, and (ii) NA-mediated cross-linking of bAP-GluA2 efficiently reduces the surface diffusion of endogenous AMPAR and eliminates their exchange between synaptic and extrasynaptic sites.

As the cross-linking effects of certain autoantibodies against GluA2 have been reported to trigger the internalization of surface-bound AMPAR (*33*, *34*), we next sought to characterize the impact of NA-mediated cross-linking on AMPAR internalization. To this end, we used kymograph plot profiles to analyze the number of internalized vesicles observed passing through a ~5-μm dendritic segment during LLSM-FRAP acquisitions and found no significant difference between mSA- and NA-labeled bAP-GluA2 (fig. S11). We then used tetrodotoxin (TTX) or NMDA treatment on mSA- or NA-labeled slices to decrease or increase the rate of activity-dependent AMPAR internalization, respectively. After 30 min, we used TEV to cleave surface-labeled bAP-GluA2 to reveal the fraction of internalized AMPAR. Again, we found no significant difference between mSA- and NA-labeled bAP-GluA2 (fig. S12). This suggests that NA is an efficient molecular tool to control AMPAR surface diffusion with minimal impact on receptor surface localization and recycling dynamics.

### Biotinylated fraction of AP-GluA2 revealed by avidin labeling correlates with GluA2 density and synapse function

**Fig. 3.**
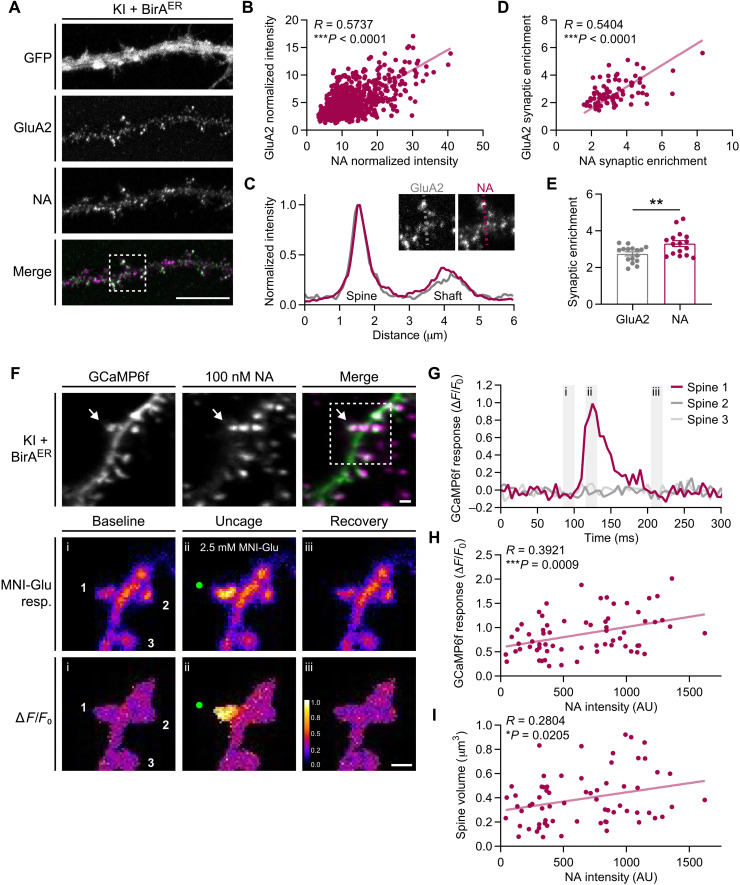
Avidin detection of bAP-GluA2 correlates with GluA2 density and synapse function. (**A**) Representative confocal images of hippocampal neuron cultures from AP-GluA2 KI mice transduced with BirA^ER^-eGFP AAV, live-labeled with NA–DyLight 633 and α-GluA2. Scale bar, 10 μm. (**B**) Correlation of GluA2 versus NA intensity at synaptic spines. *N* = 671 spines, 16 cells. ***P < 0.0001 (Pearson correlation; *R* = 0.5737). (**C**) Representative line scans across synaptic and dendritic ROIs (inserts) used to calculate the synaptic enrichment factor. (**D**) Correlation of GluA2 versus NA synaptic enrichment. *N* = 80 spines, 16 cells. ***P < 0.0001 (Pearson correlation; *R* = 0.5404). (**E**) Mean synaptic enrichment of GluA2 and NA. *N* = 16. **P = 0.0069 (unpaired *t* test). Error bars, SEM. (**F**) Representative 3D reconstructed LLSM images from CA1 pyramidal neuron dendrites in AP-GluA2 KI slice cultures transduced with BirA^ER^-Cre + FLEx GCaMP6f, incubated with NA-STAR 635P to label surface-localized bAP-GluA2 (top). Representative images of synaptic Ca^2+^ responses induced by two-photon glutamate uncaging (green dots, focal point for MNI-glutamate photolysis; middle, raw images of GCaMP6f intensity; bottom, Δ*F*/*F*_0_ averaged projection). Scale bars, 1 μm. (**G**) Plot profile of Δ*F*/*F*_0_ projection of GCaMP6f intensity at synaptic spines, as indicated in (F). Gray bars (i to iii) correspond to baseline (i), uncage (ii), and recovery (iii) time points, as indicated in (F). (**H**) Correlation of GCaMP6f glutamate uncaging synaptic response versus NA intensity over the spine surface. *N* = 68. ***P = 0.0009 (Pearson correlation; *R* = 0.3921). (**I**) Correlation of spine volume versus NA intensity over the spine surface. *N* = 68. *P = 0.0205 (Pearson correlation, *R* = 0.2804).

A critical question that arises with this AP tag KI system is the extent to which avidin detection of bAP-GluA2 reflects the actual content of GluA2-containing AMPAR on the neuronal surface. To address this point, we performed simultaneous live labeling with dye-coupled NA and the 15F1 GluA2 antibody in KI dissociated primary hippocampal cultures expressing the BirA^ER^-eGFP AAV (Fig. 3A). NA and GluA2 labeling intensity of synaptic spines was highly correlated (Fig. 3B), and the synaptic enrichment factor was proportional for the two signals (Fig. 3, C and D). However, the extent of synaptic enrichment was higher for NA than for the GluA2 antibody (Fig. 3E), likely owing to the smaller size of the NA probe and enhanced labeling access at synaptic nanodomains, in line with previous observations (*31*).

The subsequent query was whether avidin labeling intensity of bAP-GluA2 accurately reports differences in synaptic GluA2 content and can therefore be used as a proxy for synaptic strength. To this end, we added a near-infrared femtosecond (NIR fs) laser to the LLSM PSM path to perform simultaneous synaptic imaging and two-photon glutamate uncaging. We used the genetically encoded calcium indicator GCaMP6f to observe synaptic Ca^2+^ responses in organotypic slices from AP-GluA2 KI mice that were transduced with BirA^ER^-Cre + GCaMP6f AAVs and labeled with 100 nM NA conjugated to STAR 635P. We first acquired three-dimensional (3D) volume stacks of CA1 pyramidal neuron dendrites in the stratum radiatum to quantify the intensity of NA labeling over the spine surface. Then, with bath perfusion of 4-Methoxy-7-nitroindolinyl-caged-L-glutamate (MNI-glutamate) we monitored the amplitude of synaptic Ca^2+^ transients induced by two-photon glutamate uncaging during high-frequency single-plane time-lapse acquisitions (Fig. 3, F and G). NA labeling intensity was positively correlated with both the amplitude of GCaMP6f responses and spine volume (Fig. 3, H and I), indicating that variations in spine bAP-GluA2 labeling intensity reflect substantive correlated variations in AMPAR content and synaptic function. This is consistent with a previous report on the correlation between AMPA current amplitude and spine volume, and a role for AMPAR-mediated membrane depolarization in relieving Mg^2+^ block of NMDAR and/or activating voltage-gated Ca^2+^ channels (*35*, *36*). Together, these observations validate the use of avidin cell surface labeling approaches for measuring meaningful differences in protein expression, subcellular localization, and synaptic function in the AP tag KI model.

### High-resolution imaging of AP-GluA2 synaptic organization and surface mobility

**Fig. 4.**
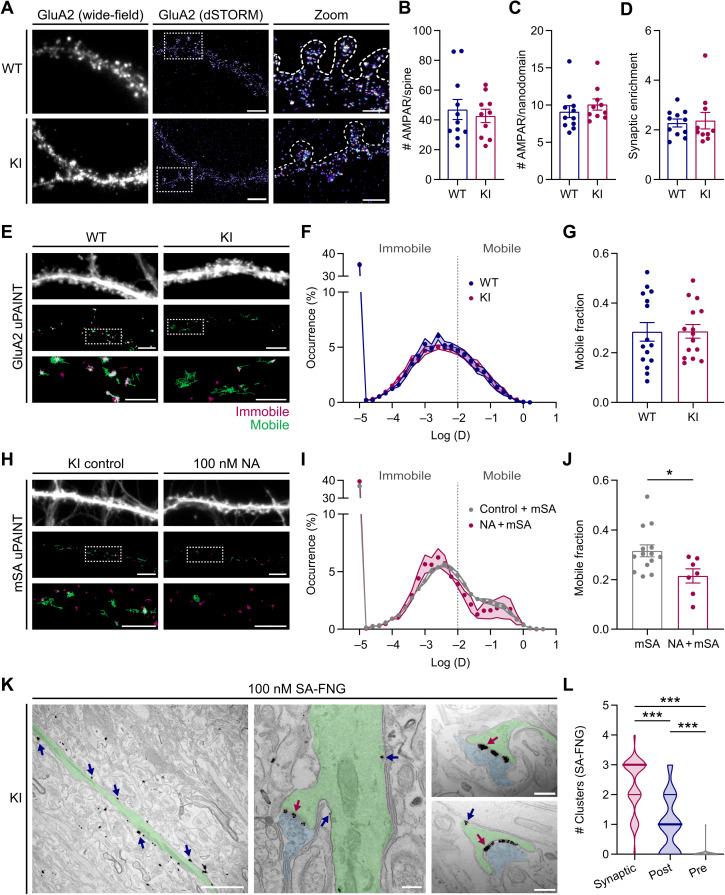
Superresolution and TEM imaging approaches with AP-GluA2. (**A**) Representative wide-field and superresolved dSTORM images of cultured WT and KI hippocampal neurons, live-labeled with α-GluA2. Scale bars, 5 and 2.5 μm. (**B** to **D**) Number of AMPARs on individual spines (B), in synaptic nanodomains (C), and AMPAR synaptic enrichment factor (D), estimated by counting the single emitters. Plots show the median per cell. *N* ≥ 10. *P* ≥ 0.1971 (Mann-Whitney *U* tests). (**E**) Representative wide-field and reconstructed uPAINT trajectory images of WT and KI neurons transduced with eGFP AAV and live-labeled with α-GluA2–SeTau 647 [mobile GluA2 trajectories in green and immobile GluA2 trajectories in red (log(*D*) ≥ or ≤ −2, respectively)]. (**F** and **G**). Average distribution of the logarithm of diffusion (F) and ratio of mobile over immobile fraction (G). *N* = 15. *P* = 0.9675 (unpaired *t* test). (**H**) Representative images of KI neurons transduced with BirA^ER^-eGFP AAV, incubated with vehicle control or 100 nM unconjugated NA. mSA-STAR 635P (7.8 nM) was used to record uPAINT trajectories of bAP-GluA2. Scale bars, 5 and 2.5 μm. (**I** and **J**). Average distribution and ratio, as in (F) and (G). *N* ≥ 7. *P = 0.0193 (unpaired *t* test). Error bars, SEM. (**K**) Representative TEM images of bAP-GluA2 in CA1 pyramidal neurons from AP-GluA2 KI slice cultures transduced with BirA^ER^-eGFP AAV and incubated with 100 nM SA conjugated to FluoroNanogold (SA-FNG) and then processed with silver enhancement for visualization. Left: An apical dendrite (shaded green) with multiple SA-FNG clusters (arrows). Middle: A dendritic spine and presynaptic bouton (shaded blue), with synaptic (red arrow) and extrasynaptic SA-FNG clusters (blue arrows). Right: Representative images used to quantify SA-FNG cluster localization to synaptic cleft (red arrow), postsynaptic spine (blue arrow), or presynaptic bouton (not shown/infrequently detected). Scale bars, 2 μm and 200 nm. (**L**) Subcellular distribution of SA-FNG clusters; plot shows the median with first and third quartiles. *N* = 39 synapses, three experiments. ***P < 0.0001 (Kruskal-Wallis test; *F* = 74.88, *P* < 0.0001; Dunn’s post hoc test). See also fig. S13.

As the N-terminal domain of AMPAR subunits has been reported to influence synaptic organization and surface mobility dynamics (*14*, *37*), we next performed direct stochastic optical reconstruction microscopy (dSTORM) and universal point accumulation for imaging in nanoscale topography (uPAINT) superresolution imaging to characterize AMPAR synaptic nanoscale organization and surface diffusion in dissociated hippocampal neuron cultures from AP-GluA2 KI or WT mice. Surface AMPAR were live-labeled with the 15F1 GluA2 antibody before fixation and observed by dSTORM using an Alexa Fluor 647–coupled secondary antibody. The number of AMPAR was estimated on the basis of the blinking properties of the fluorophore (*38*). We found no difference in the number of AMPAR at spines, in synaptic nanodomains, or in the synaptic enrichment factor between WT and KI cultures (Fig. 4, A to D). For uPAINT, surface GluA2 were live-labeled with a low concentration of SeTau 647–coupled 15F1 to measure total surface AMPAR diffusion. We found no difference in the distribution of diffusion coefficients or the fraction of mobile receptors between KI and WT cultures (Fig. 4, E to G). This suggests that the modified N-terminal sequence in the AP-GluA2 KI model does not affect AMPAR synaptic organization and mobility.

We then used a low concentration of STAR 635P–coupled mSA to monitor AMPAR surface diffusion by tracking bAP-GluA2. To evaluate the impact of NA cross-linking on AMPAR lateral diffusion, we pretreated cultures with unlabeled NA before the application of mSA, reasoning that the remaining free biotin sites within the pool of NA–cross-linked surface AMPAR would be accessible to dye-conjugated mSA. NA application shifted the distribution of bAP-GluA2 diffusion coefficients toward lower values, with a concomitant decrease in the fraction of mobile receptors (Fig. 4, H to J), consistent with our observations of NA-mediated immobilization in organotypic slices by LLSM-FRAP (see Fig. 2, E and F).

Next, we took advantage of the large catalog of commercially available SA conjugates and used FluoroNanogold (SA-FNG) to characterize the distribution of bAP-GluA2–containing AMPAR in organotypic slices by transmission electron microscopy (TEM) (fig. S13). We quantified the distribution of silver-enhanced SA-FNG clusters on CA1 stratum radiatum synaptic micrographs and found that clusters were predominantly localized to the synaptic cleft and postsynaptic spine membranes, but infrequently associated with presynaptic bouton membranes identified by synaptic vesicle content (Fig. 4, K and L). Together, these observations demonstrate the utility of the AP-GluA2 KI model and small avidin probes to study the nanoscale organization and diffusion dynamics of endogenous AMPAR with high-resolution imaging approaches.

### Biochemical characterization confirms WT expression levels of AP-GluA2

**Fig. 5.**
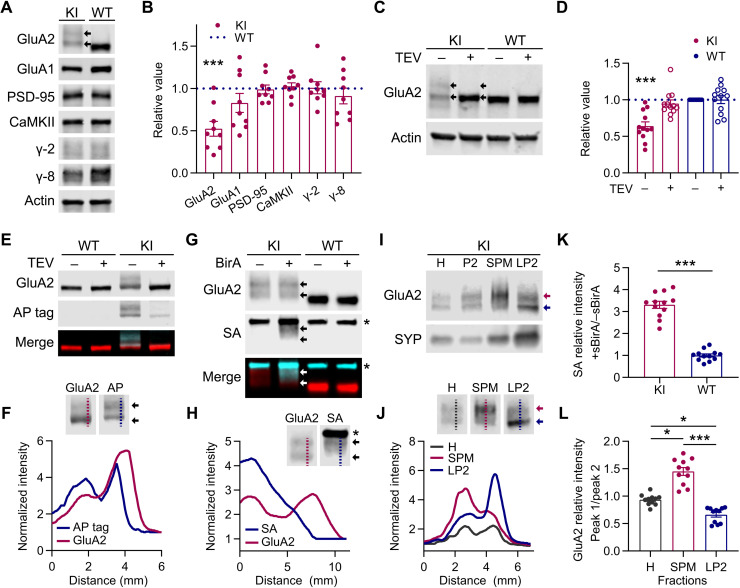
Biochemical characterization of AP-GluA2 expression and localization. (**A**) Representative Western blots of AP-GluA2 KI or WT protein samples (whole brain or hippocampal lysate). Double banding is observed for AP-GluA2 KI (arrows). (**B**) Quantification of KI protein expression relative to WT and normalized to β-actin loading control. *N* = 9. ***P = 0.0006 (one-sample *t* tests). (**C**) In vitro incubation of KI or WT protein samples with TEV protease cleaves the AP tag and resolves GluA2 to a single band (arrows) with the same relative expression as WT. (**D**) Quantification of GluA2 expression relative to WT (−TEV) and normalized to β-actin loading control. *N* = 12. ***P = 0.0005 (Wilcoxon signed-rank test). (**E**) Dual labeling of AP tag and GluA2 in WT or KI protein samples with or without TEV protease. (**F**) Line scans (dashed lines in insets) reveal partial colocalization of AP tag and lower GluA2 band (arrows). (**G**) In vitro incubation of KI or WT protein samples with sBirA + biotin-AMP; SA binds bAP-GluA2 in the upper GluA2 band (arrows). Asterisks denote SA binding to endogenous biotin binding proteins. (**H**) Line scans reveal colocalization of SA with the upper but not lower GluA2 band. (**I**) Subcellular fractionation of hippocampal lysate (H, homogenate; P2, crude membranes) shows enrichment of the upper AP-GluA2 band in the synaptic plasma membrane fraction (SPM) and the lower band in the vesicular fraction (LP2). (**J**) Line scans show differential sorting of AP-GluA2 populations to the membrane (SPM, red line and arrow) and intracellular vesicles (LP2, blue line and arrow). (**K**) Quantification of SA binding (+sBirA relative to −sBirA). *N* = 12. ***P < 0.0001 (unpaired *t* test). (**L**) Quantification of relative AP-GluA2 distribution (upper, peak 1; lower, peak 2) in line scans from blots of hippocampal lysate (H), membrane (SPM), and vesicular (LP2) fractions. *N* = 11. *P ≤ 0.0337, and ***P ≤ 0.0001 (Kruskal-Wallis test; *F* = 27.08, *P* < 0.0001; Dunn’s post hoc test). See also figs. S14 to S17.

To characterize AP-GluA2 protein expression in the KI model, we performed Western blot analysis of KI or WT brain protein lysates. The assay of several synaptic proteins indicated no differences in expression, except for AP-GluA2, which unexpectedly appeared as a smeared double band with reduced apparent expression when quantified (Fig. 5, A and B, and fig. S14). However, when protein samples were incubated with TEV to cleave the AP tag, GluA2 resolved to a single band in the AP-GluA2 KI with the same relative expression as in WT (Fig. 5, C and D, and fig. S14). As the observed shift in the apparent molecular weight of AP-GluA2 is larger than would be expected for the addition of 48 amino acids encoding the AP-TEV and linker sequence (~5 kDa), we used various glycosidases to evaluate the glycosylation state AP-GluA2. We found that deglycosylation did not affect the double banding pattern (fig. S15A). While both upper and lower GluA2 bands were recognized by an AP tag antibody, the overlap between α-AP tag and α-GluA2 signals was incomplete, with the lower part of the faster migrating GluA2 band not recognized by the α-AP tag (Fig. 5, E and F, and fig. S15B). When we incubated the protein samples with sBirA and biotin-AMP, SA binding to bAP-GluA2 overlapped with the GluA2 upper band and intermediate smear, but was absent from the lower band (Fig. 5, G, H, and K, and fig. S16). This suggests that the lower band represents an N-terminal degradation product of AP-GluA2, which has lost the biotinylated lysine. We then performed subcellular fractionation to assay the distribution of AP-GluA2 protein and found that the upper band of AP-GluA2 was enriched in the synaptic membrane fraction, whereas the lower band was enriched in the vesicular fraction (Fig. 5, I, J, and L, and fig. S17). We found no difference in the synaptic enrichment profiles of GluA2 or GluA1 AMPAR subunits between KI and WT samples (fig. S17C). These observations indicate that most surface GluA2 in the KI model carry the AP tag conducive to biotinylation by BirA, and therefore recognition by biotin binding proteins.

**Fig. 6.**
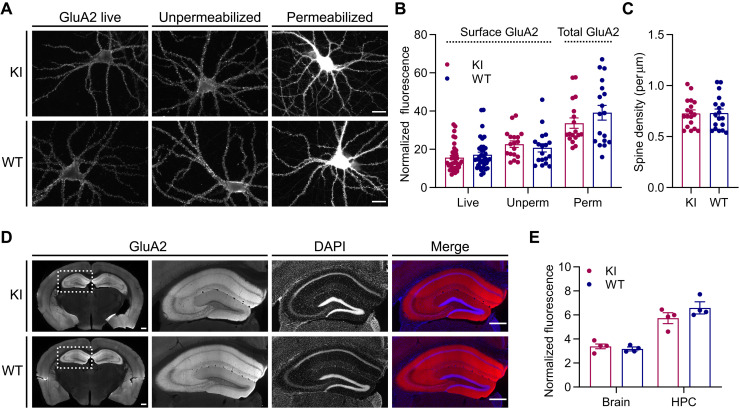
Immunological characterization of AP-GluA2 expression and localization. (**A**) Representative wide-field images of KI and WT hippocampal neurons in primary dissociated culture labeled with α-GluA2 live (left), after fixation (middle), or after fixation and permeabilization (right). Scale bars, 20 μm. (**B**). Normalized fluorescence intensity of surface or whole-cell GluA2. *N* ≥ 18. *P* ≥ 0.2649 (Mann-Whitney *U* tests). (**C**) Spine density in KI and WT hippocampal neuron cultures. *N* = 18. *P* = 0.9484 (unpaired *t* test). (**D**) Representative tiled wide-field images of 50-μm frontal sections from KI or WT brains labeled with α-GluA2. Scale bars, 500 μm. (**E**) Fluorescence intensity of GluA2 in KI or WT sections, normalized to secondary-only control. *N* = 4. *P* ≥ 0.1682 (unpaired *t* tests). Error bars, SEM. See also fig. S18.

We then used dissociated primary hippocampal cultures and frontal brain sections from KI and WT mice to perform immunological characterization of GluA2 expression and localization. We found no difference in the amount of GluA2 at the neuronal surface in live-labeled or fixed-unpermeabilized neurons in culture, or in the total GluA2 content of fixed-permeabilized neurons (Fig. 6, A and B). Images of GluA2-labeled fixed-permeabilized neurons were used to quantify spine density, and we found no difference between KI and WT cultures (Fig. 6C). Whole brain sections from KI and WT adult mice also revealed no changes in GluA2 abundance (Fig. 6, D and E, and fig. S18). Together, these data confirm normal GluA2 expression and subcellular localization in the AP-GluA2 KI model.

### AP tag and biotinylation does not affect AMPAR or synaptic function in the AP-GluA2 KI model

**Fig. 7.**
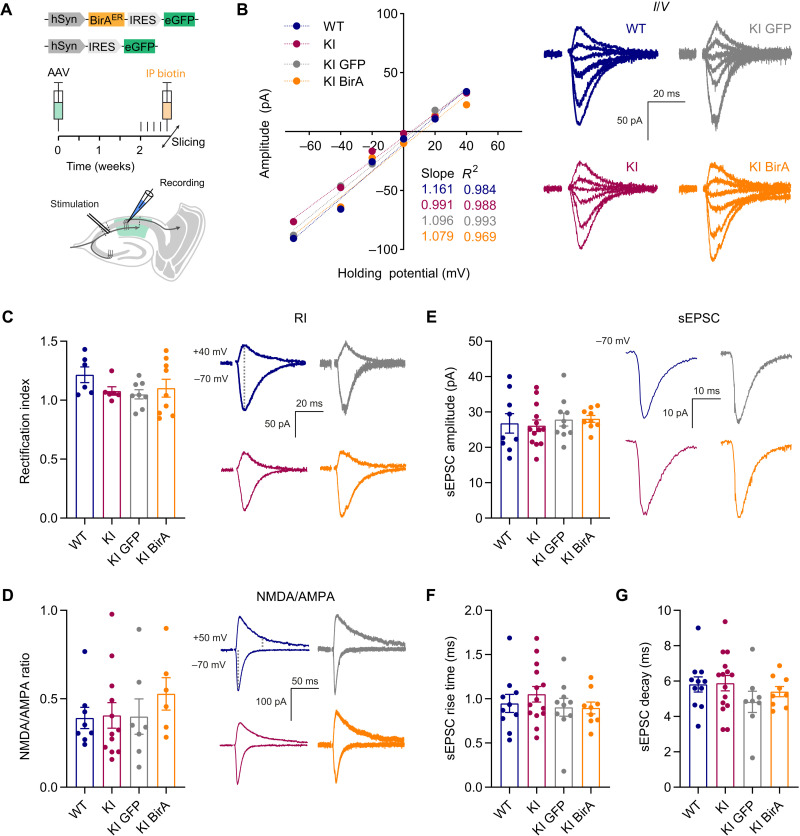
Electrophysiological characterization of AP-GluA2 KI. (**A**) Overview of the experimental preparation, AAV stereotaxic injection in the dorsal hippocampus followed by intraperitoneal (IP) biotin injection (top), and whole-cell voltage-clamp electrophysiological recordings of CA1 pyramidal neurons in acute slices from adult mice to measure spontaneous or evoked synaptic responses by stimulation of Schaffer collaterals (bottom). (**B**) Representative current-voltage (*I*/*V*) plots of synaptic AMPA current amplitudes with fitting to a simple linear regression (WT in blue, KI in red, KI + eGFP AAV in gray, and KI + BirA^ER^-eGFP AAV in orange). (**C**) Rectification index (RI) of synaptic AMPA currents. *N* ≥ 6. *P* ≥ 0.3020 (Kruskal-Wallis test; *F* = 4.135, *P* = 0.2473; Dunn’s post hoc test). (**D**) Ratio of synaptic NMDA and AMPA currents. *N* ≥ 6. *P* ≥ 0.9153 (Kruskal-Wallis test; *F* = 2.231, *P* = 0.5258; Dunn’s post hoc test). (**E** to **G**). Characterization of the amplitude (E), rise time (F), and decay time (G) of sEPSC. *N* ≥ 9. *P* ≥ 0.3840 (one-way ANOVA; *F* = 0.2545, *P* = 0.8576; *F* = 0.6686, *P* = 0.5764; *F* = 1.010, *P* = 0.3984; Tukey’s post hoc test). Representative traces and holding potentials are shown on the right of relevant panels. Error bars, SEM. See also fig. S19 for further synaptic and network characterization.

We next evaluated the impact of AP-GluA2 KI and BirA^ER^ expression on synaptic and AMPAR channel function in adult brain circuits using whole-cell voltage-clamp electrophysiological recordings in acute hippocampal slices from KI and WT mice. To achieve in vivo biotinylation of AP-GluA2, we performed stereotaxic injection of AAVs encoding BirA^ER^-eGFP or eGFP control into the CA1 region of AP-GluA2 KI mice. Biotin supplementation was achieved by five consecutive days of intraperitoneal injections before the preparation of acute slices. The amplitude and rectification of evoked AMPAR-mediated responses (synaptic current-voltage responses, rectification index, and NMDA/AMPA ratio) and spontaneous excitatory postsynaptic currents (sEPSCs) were all indistinguishable among WT, KI, and KI + eGFP or KI + BirA^ER^ cells (Fig. 7, A to G). We also found that excitatory/inhibitory balance and spontaneous inhibitory postsynaptic currents (sIPSCs) were unaffected by the modifications (fig. S19). This is in line with our previous characterization of the biophysical properties of AP-tagged GluA2 subunits (*25*) and indicates that bAP-GluA2–containing AMPARs are fully functional and that the genetic modification of GluA2 does not affect basal synaptic or network physiology.

### Cross-linking of bAP-GluA2 by NA precludes LTP in the hippocampal CA1 region

**Fig. 8.**
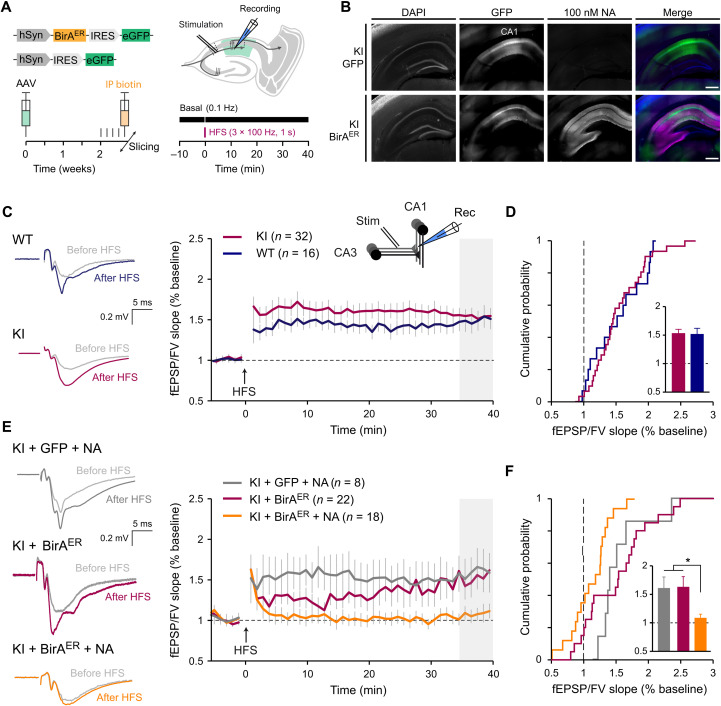
AMPAR immobilization by NA cross-linking precludes LTP. (**A**) Overview of the experimental preparation, AAV stereotaxic injection in the dorsal hippocampus followed by intraperitoneal biotin injection (left), and field recordings of LTP in CA1 evoked by HFS (3 × 1-s trains, 100 Hz) of Schaffer collaterals in acute slices (right). (**B**) Representative tiled wide-field images of 300-μm frontal brain sections from KI mice injected with eGFP control or BirA^ER^-eGFP AAV, live-labeled with 100 nM NA conjugated to DyLight 633. Scale bars, 500 μm. (**C** and **D**) HFS-induced LTP in acute slices from KI and WT mice (KI in red; WT in blue), representative voltage traces and summary time courses (C), and cumulative histograms (D) of mean normalized fEPSP/FV slope 35 to 40 min after HFS induction (shaded gray). *N* ≥ 16. *P* = 0.8580 (unpaired *t* test). Statistical comparison of LTP was 35 to 40 min after HFS. Number of slices is indicated in brackets. (**E** and **F**) HFS-induced LTP in acute slices from KI mice injected in CA1 with eGFP control or BirA^ER^-eGFP AAV (eGFP + NA in gray, BirA^ER^ without NA in red, and BirA^ER^ + NA in orange); slices were incubated with 100 nM NA and then continuously perfused with 10 pM NA, as in (C) and (D). *N* ≥ 8. *P ≤ 0.0365 (Kruskal-Wallis test; *F* = 8.812, *P* = 0.0122; Dunn’s post hoc test). Error bars, SEM. See also fig. S20 for basal transmission controls.

To determine whether LTP is intact at CA3 to CA1 Schaffer collaterals in AP-GluA2 KI mice, we used high-frequency stimulation (HFS; 3 × 1 s at 100 Hz) to induce LTP at CA3-CA1 synapses in acute slices from KI and WT mice. Under basal conditions, we found that input/output curves were indistinguishable (fig. S20). HFS application was followed by a significant increase in the excitatory postsynaptic potential/fiber volley (fEPSP/FV) slope ratio, with a comparable increase in the synaptic response in both groups (Fig. 8, A, C, and D), indicating that appropriate synaptic plasticity is maintained with the AP-GluA2 genetic modification.

To achieve in vivo biotinylation of AP-GluA2, we performed stereotaxic injection of AAVs encoding BirA^ER^-eGFP or eGFP control into the CA1 region of AP-GluA2 KI mice, with biotin supplementation as above. To achieve AMPAR immobilization, NA (100 nM) was preincubated for 30 to 60 min before slices were transferred to the recording chamber, and field recordings were made from areas with a high density of eGFP-expressing neurons in artificial cerebrospinal fluid (ACSF) containing 10 pM NA to immobilize newly exocytosed receptors (Fig. 8B) (*25*). We compared the level of LTP expression after HFS in slices from KI + eGFP + NA or KI + BirA^ER^ (without NA), which lack the necessary conditions for AMPAR cross-linking, and KI + BirA^ER^ + NA to determine the impact of cross-linking bAP-GluA2. LTP levels remained normal in both KI + eGFP + NA and KI + BirA^ER^ control slices but were almost completely abolished when bAP-GluA2–containing AMPARs were cross-linked in KI + BirA^ER^ + NA slices. (Fig. 8, E and F). Notably, we observed an initial posttetanic potentiation, most likely of presynaptic origin, which remained unaffected in the presence of NA and bAP-GluA2. These results demonstrate that NA-mediated immobilization of bAP-GluA2–containing AMPAR is an efficient tool to control the expression of activity-dependent synaptic plasticity without affecting basal synaptic network function.

### Cross-linking of bAP-GluA2 by NA prevents the formation of contextual fear memory

**Fig. 9.**
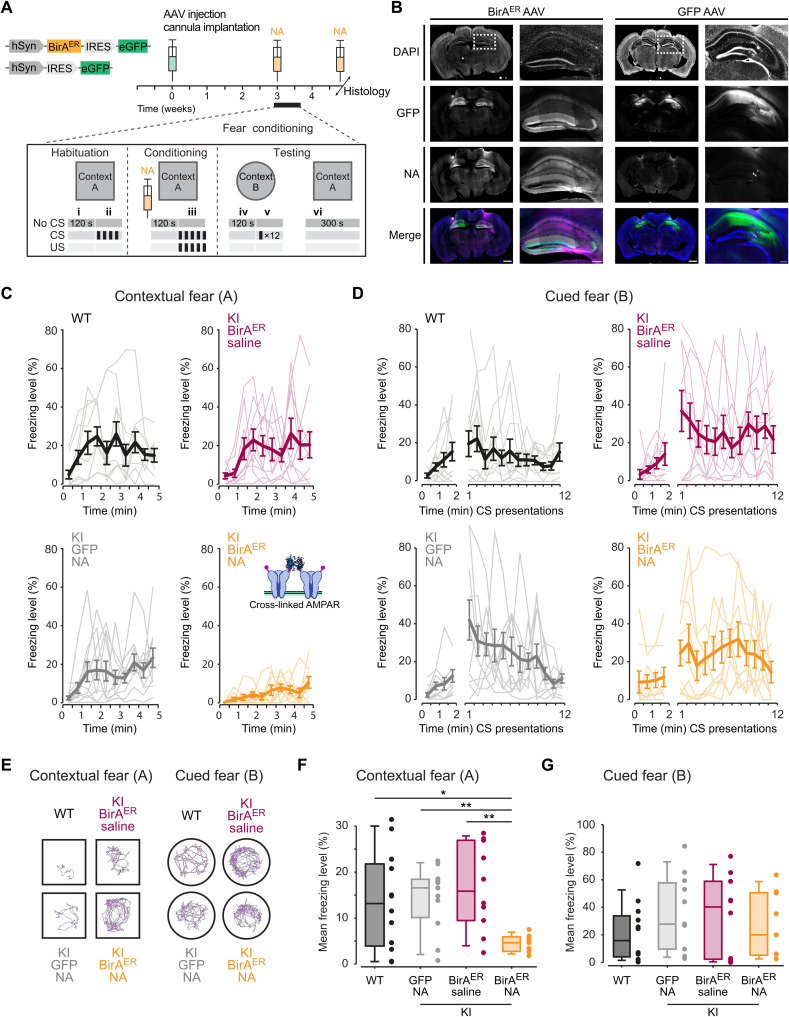
AMPAR immobilization by NA cross-linking blocks formation of contextual fear memories. (**A**) Overview of the experimental design, stereotaxic injection of BirA^ER^-eGFP or eGFP control AAV in the dorsal hippocampus and surgical implantation of the cannula used to deliver NA before fear acquisition, and postmortem histology to verify infusion sites (i and ii: basal freezing; iii: fear acquisition; iv: contextual fear control; v: cued fear expression; vi: contextual fear expression). (**B**) Representative tiled wide-field images of 60-μm frontal brain sections from KI mice injected with BirA^ER^-eGFP or eGFP control AAV and infused with 8.33 μM NA conjugated to Texas Red by implanted cannula. Scale bars, 1000 and 250 μm. (**C** and **D**) Time course of freezing behavior expression observed during contextual fear memory testing (C) and upon CS presentation during cued fear memory testing (D). (**E**) Representative traces of mouse movements during the first 2 min of exposure to contexts A and B. (**F**) Mean freezing levels during contextual fear memory testing. *N* ≥ 9. *P ≤ 0.0391 and **P ≤ 0.0074 (Welch’s ANOVA; *F* = 12.13, *P* = 0.0001; Dunnett’s post hoc test). (**G**) Mean freezing levels during cued fear memory testing. *P* ≥ 0.7825 (one-way ANOVA; *F* = 0.6950, *P* = 0.5609; Dunnett’s post hoc test). See also figs. S21 and S22.

LTP mechanisms in the dorsal hippocampus have been linked to memory acquisition in vivo (*39*), and we have previously reported that antibody-mediated cross-linking of AMPAR surface diffusion impaired the formation of contextual fear memories in mice (*25*). Therefore, we reasoned that cross-linking of bAP-GluA2 with NA would afford target-specific control of memory formation and fear behavior in AP-GluA2 KI mice upon the expression of BirA^ER^. To this end, we performed bilateral stereotaxic injection of BirA^ER^-eGFP or eGFP AAVs in the CA1 region of AP-GluA2 KI mice and implanted guide cannulas to allow infusion of NA into the dorsal hippocampus in vivo (Fig. 9, A and B). Compared to the control conditions of nonmanipulated WT mice, KI + BirA^ER^ + saline infusion or KI + eGFP + NA infusion, KI mice injected with BirA^ER^ and infused with NA to immobilize bAP-GluA2–containing AMPAR exhibited significantly reduced levels of freezing when reexposed to the fear-conditioned context (Fig. 9, C, E, and F; vi in Fig. 9A). All the groups exhibited normal mobility, developed freezing responses to CS+/US pairings (US: unconditioned stimulus, mild foot shocks; CS: conditioned stimulus, 7.5-kHz tones), and exhibited hippocampus-independent cued fear memory the following days when reexposed to CS+ (Fig. 9, D, E, and G; v in Fig. 9A; see also figs. S21 and S22). This demonstrates that in vivo immobilization of surface-diffusing AMPAR with NA offers a new approach to control associative memory and opens to target-specific control of behaviorally relevant synaptic plasticity expression in vivo.

## DISCUSSION

Advancements in understanding the organization and function of the brain are constrained by the limitations of currently available techniques, where experimental access to appropriate spatiotemporal resolutions, endogenous proteins, and opportunities to assay synaptic and neuronal function within complex integrated circuits remain formidable challenges. Bridging this gap requires parallel developments in high-resolution imaging methods and molecular tools to visualize and functionalize proteins of interest. In this study, we have developed and characterized a new mouse model system and an associated molecular toolkit, where genetic KI of the AP tag and target-specific regulation of BirA expression allow the surface trafficking dynamics of endogenous AMPAR to be monitored and manipulated with avidin probes that efficiently access confined synaptic domains in thick biological tissues. By tuning BirA expression, the resolution of this system can be scaled from the study of single molecules in individual neurons to integrated circuits in behaving animals. This opportunity for sparse, target-specific labeling of endogenous surface AMPAR offers a substantial advantage for high-resolution synaptic imaging applications in tissue preparations, which would otherwise be obscured by the high signal density in constitutive genetic KI models. We furthermore anticipate that this technology will be readily adaptable to studying and controlling the nanoscale organization and trafficking dynamics of most cell surface proteins, which can now be accomplished by AP tag functionalization of endogenous proteins with relative ease using CRISPR-Cas9–mediated genome editing of animal models or experimental preparations (*40*–*43*). This is further reinforced by the emerging roles for the lateral diffusion of synaptic adhesion molecules, presynaptic voltage-gated calcium channels, and astrocytic glutamate transporters in shaping synapse assembly, function, and plasticity (*44*–*46*). Together, these features promise to open new avenues of investigation compatible with a wide range of experimental techniques and biological research questions.

We demonstrate that genetically modified AP-GluA2 subunits exhibit normal protein expression, localization, and function and that AP-GluA2 KI mice exhibit normal AMPAR synaptic composition, physiology, circuit function, and behavior. Several lines of evidence indicate that AP-GluA2 KI mice form receptors with comparable stoichiometry and that the different AMPAR subunits (AP-GluA2 and GluA1, in particular) are present at normal levels at synapses. First, we found no differences in rectification index at hippocampal CA3-CA1 synapses (Fig. 7C) or in fractionation experiments (fig. S17) between KI and WT mice. Second, basal synaptic transmission was indistinguishable in WT and KI, and with AP-GluA2 biotinylation upon the expression of BirA, as seen from normal *I*/*O* curves and AMPA/NMDA ratio (Fig. 7D and fig. S20). Third, with quantitative dSTORM, we found indistinguishable GluA2 synaptic nanoscale organization and enrichment in neurons cultured from KI and WT mice (Fig. 4, A to D). Last, we found similar hippocampal CA1 spine density between KI and WT slices with and without BirA expression (fig. S5). These controls are important as GluA N-terminal domains have fascinating emerging roles in AMPAR synaptic targeting and stabilization (*14*, *37*, *47*–*50*), and tagging could affect their trafficking. N-terminal epitope tagging of GluA2 does not seem to affect its synaptic targeting or function [this study and (*14*)]. Furthermore, while N-terminal GFP tagging of GluA1 has been initially reported to affect its synaptic targeting (*14*), lengthening and optimization of the linker sequence to increase the flexibility of SEP tag fusions has been shown to limit disruption of GluA1 function in a SEP-GluA1 KI model (*11*). However, this mouse line was found to exhibit substantial reductions in GluA1 protein expression and synaptic localization, with compensation by upscaling of GluA2/3 (*11*). In our case, the AP N-terminal tag is small and nonstructured, with an extensive linker. Thus, in principle, this KI sequence was unlikely to affect AMPAR synaptic targeting and function. All of our control experiments have pointed to an absence of effect of the N-terminal AP tag on AMPAR function and targeting. This suggests that genetic KI of small sequences such as the AP tag is an advantageous strategy for functionalizing AMPAR subunits that better conserves normal expression patterns and synaptic composition. However, one puzzling observation in the AP-GluA2 KI that is at present difficult to explain is the slower migration and unusual banding pattern of AP-GluA2 in Western blots (Fig. 5 and figs. S14 to S17). We could not find evidence for a role of posttranslational modification in this migration and banding pattern. After extensive characterization by subcellular fractionation and immunoreactivity assays, we propose as the most parsimonious explanation that the AP tag is proteolytically degraded within intracellular compartments but remains intact in the biogenesis pathway and on the cell surface. At this point, we have no concrete explanation as to why GluA2 migration is altered by the presence of the AP-TEV KI sequence.

Exploitation of the AP-GluA2 KI mouse model goes hand in hand with expression of the biotinylation enzyme BirA. Our control experiments indicate that BirA expression and biotinylation of the AP tag do not modify AMPAR channel properties, synaptic function, or animal behavior. CA1 pyramidal neurons expressing BirA in acute slices from AP-GluA2 KI mice exhibited similar basal synaptic properties such as NMDA/AMPA ratio, rectification index, sEPSC amplitude, rise time, or decay (Fig. 7), and the level of LTP at Schaffer collaterals was identical among the various control conditions (Fig. 8 and fig. S20). Together, our data establish that BirA expression in itself does not modify in any detectable manner basal synaptic transmission. Notably, BirA is an extremely efficient enzyme that is able to biotinylate its targets at very low expression levels (*51*). Accordingly, while the absence of BirA yielded no biotinylation of AP-GluA2, we could find avidin binding to bAP-GluA2 in brain regions with undetectable levels of the eGFP reporter after injection of the BirA^ER^-GFP AAV virus (fig. S8). A related question pertains to the dependence of AP-GluA2 biotinylation on exogenously added biotin in the various experimental conditions. In culture, we performed systematic biotin supplementation to ensure that receptors were maximally biotinylated, as we had observed in initial experiments that the absence of biotin supplementation in some preparations leads to suboptimal biotinylation. In vivo, we initially performed intraperitoneal biotin injections for this reason but found that we could obtain adequate AP-GluA2 biotinylation and avidin labeling without biotin supplementation. With long-term expression of BirA following AAV stereotaxic injection, the endogenous levels of biotin in vivo are likely sufficient to saturate the system. This efficacy precluded us from pursuing further detailed study of the impact of BirA expression levels and biotin supplementation with respect to the levels of AP-GluA2 biotinylation and avidin labeling.

As naturally biotinylated proteins are largely absent from cell surface membranes (*31*, *52*), we found avidin labeling to be highly specific to bAP-GluA2 and tightly controlled by the expression or application of BirA. One limitation of this approach is the opportunity to quantify absolute receptor numbers at the synapse, as a small proportion of AP-GluA2 at the neuronal membrane may remain unbiotinylated and therefore undetectable by avidin probes. Note, however, that this type of constraint exists for all protein labeling or genetic tagging strategies (*53*). Together with the remarkably high catalytic efficiency of BirA (*51*), our observations that AP-GluA2 biotinylation saturated over time with chronic and acute BirA approaches (Fig. 1, E and I) and that non–bAP-GluA2 were largely confined to intracellular compartments (Fig. 5, E to L) suggest that the amount of unbiotinylated AP–GluA2 at the neuronal surface is minor. Along this line, we found that bAP-GluA2 labeling intensity was largely proportional to AMPAR density and synapse strength, as evidenced by simultaneous GluA2/avidin labeling and the correlative increases in intensity for avidin labeling, synaptic calcium responses, and spine volume (Fig. 3). Furthermore, we found a comparable, although slightly higher, level of synaptic AMPAR enrichment revealed by avidin versus a GluA2 antibody (Fig. 3E). This difference can be explained by the smaller size and better synaptic access of NA (*31*). Notably, within individual neurons, we did not observe a decrease in AP-GluA2 biotinylation levels with distance from the soma. We expect AP-GluA2 to be homogeneously biotinylated within the dendrite, as BirA^ER^ is resident in the ER (*28*) and the dendritic ER compartment is continuous and represents a highly permissive environment for diffusion (*54*, *55*). Together, this indicates that the detection of bAP-GluA2 is highly correlated with the absolute number of GluA2-containing AMPAR and therefore synaptic strength.

The LLSM imaging modality permits high-resolution, high-speed imaging with low photobleaching and phototoxicity, which has enabled nanoscale imaging of fast dynamic processes in live tissues and organisms (*56*), and permitted previously unidentified insights into the morphological organization of the brain by coupling expansion microscopy with the rapid large-volume imaging capacity of LLSM (*57*). Our development of a PSM that allows simultaneous imaging and one- or two-photon manipulation considerably advances the functionality of LLSM setups by permitting all-optical physiological studies in live tissue preparations with enhanced 4D spatiotemporal resolution (*58*). The combination of LLSM-FRAP, two-photon uncaging, and mSA or NA labeling of bAP-GluA2 enabled characterization of the mobility/immobilization dynamics of endogenous AMPAR as well as quantification of synapse strength in an integrated brain slice preparation (Figs. 2 and 3). Whereas previous studies characterizing AMPAR mobility dynamics in hippocampal slices or in vivo have mostly used overexpression of SEP-tagged AMPAR subunits (*6*, *8*, *23*), few have accomplished measurement of endogenous AMPAR, with the exception of chemical labeling of native AMPAR complexes in acute slices (*12*, *13*). Previous measurements of synaptic AMPAR mobile fractions using SEP overexpression approaches ranged from ~30 to 100% and are generally higher than what we have found in the present study or what has been found on endogenous AMPAR in cultured neurons (*41*, *59*). This lends support to the notion that measurements of AMPAR surface diffusion dynamics are affected by experimental approaches that manipulate the content of AMPAR surface pools through overexpression, and the importance of measuring the properties of receptors expressed at endogenous levels. Tetravalent cross-linking of bAP-GluA2 by NA application efficiently decreased AMPAR surface mobility at synaptic sites, while extrasynaptic AMPAR remained partly mobile. We suspect that some extrasynaptic receptors escape cross-linking because of the low density on the dendritic shaft, or that small clusters may remain mobile if none of the components are bound to stable anchoring structures, as in the PSD. This likely explains why AMPAR internalization, which occurs at extrasynaptic sites (*15*, *16*), was not affected by AMPAR cross-linking in our measurements (figs. S11 and S12).

We urgently need new strategies to progress on understanding the relationship between synaptic plasticity and neuronal network rearrangements induced by memory formation. Several open questions remain, such as the importance of synaptic plasticity mechanisms for encoding, consolidation, and retrieval of memories, as well as the role of specific sleep phase oscillations in reactivating and selecting inputs potentiated during memory encoding (*1*, *60*). As the vast majority of excitatory synapses onto pyramidal cells express postsynaptic LTP using GluA2-containing AMPAR (*61*–*63*), we expect that the spatiotemporal control of AMPAR mobility in vivo afforded by this experimental model will allow progress in establishing the causality link between synaptic plasticity and memory dynamics. Here, we targeted the GluA2 AMPAR subunit for proof-of-principle development of an AP tag KI model, as in our previous work (*25*) we could fully block LTP in hippocampal pyramidal neurons and in vivo through cross-linking surface GluA2-containing AMPAR. We are unsure what would be the consequence of cross-linking only GluA1-containing AMPAR using this approach. Furthermore, the role or existence of transient incorporation of GluA1 homomers during LTP in hippocampal pyramidal neurons is still hotly debated (*32*). If, as hypothesized (*32*), GluA1 homomers are only transiently incorporated at synapses during some forms of LTP, we cannot be certain that our cross-linking method would capture this event. While we expect the present cross-linking strategy to be highly efficient for blocking synaptic plasticity at most excitatory synapses, two limitations must be noted. First, plasticity must remain unaffected at synapses that express presynaptic LTP, such as hippocampal mossy fibers (*64*, *65*). Second, a degree of insensitivity must also exist at excitatory contacts onto interneurons, which have been shown to express synaptic plasticity upon learning and often contain GluA1 homomers (*66*–*68*). Transposing this strategy to the development of AP-GluA1 KI mice should be useful for the study of AMPAR mobility and synaptic plasticity at these excitatory synapses onto interneurons.

The future development of molecular strategies to control cell type–specific or activity-dependent BirA expression holds great potential to answer as-yet inaccessible questions regarding the role of synaptic plasticity for memory formation in discrete cell populations. For example, enhancer-promoter sequences may be useful to restrict AAV-mediated BirA expression to defined cell types (*69*), and AAV-PHP.eB capsids for noninvasive gene delivery to the central nervous system (CNS) may allow BirA transduction of distinct cell populations that are broadly distributed throughout the brain (*70*). Moreover, coupling synaptic plasticity blockade with recent techniques to restrict BirA expression to neurons activated during specific behavioral modalities (e.g., FliCRE and Cal-light) would be of great interest to test the importance of a neuron’s activity profile in its future involvement in memory engrams or behaviorally relevant neuronal ensembles (*71*, *72*). We should note, however, that while expressing BirA under cell- or activity-specific promoters is an exciting future direction for controlling receptor trafficking and synaptic plasticity, it will require an extensive development and characterization of molecular tools. In particular, over the course of the present study, we realized that BirA is an extremely efficient enzyme that biotinylates its targets at very low expression levels (fig. S8). Hence, achieving the necessary regulation of BirA for more precise target-specific applications will require further development of tightly controlled expression systems. Additional applications of the technology presented here include the capacity to perform in vivo imaging of endogenous AMPAR levels and dynamics. This is, in principle, feasible with the current tools, although it requires the injection of fluorescent mSA together with the implementation of a cranial imaging window (*73*), and would likely also require protein engineering developments to improve the mSA fluorescence signal.

In conclusion, this AP tag KI mouse model and molecular toolkit represents a new genetic labeling strategy that enables target-specific measurement and control of the mobility dynamics of endogenous cell surface proteins in integrated experimental systems. For studying AMPAR dynamics, this model opens opportunities to explore as-yet unanswered questions regarding the molecular links between receptor surface mobility, synaptic plasticity, and behavioral adaptation. With the CRISPR-Cas9 revolution in targeted genome engineering, this methodology can be adapted for the study of a large variety of synaptic proteins, with broad implications for advancing our understanding of brain organization and function.

## METHODS

### Animals

The AP-GluA2 KI model was generated by the PHENOMIN Mouse Clinical Institute (Strasbourg, France) using CRISPR-Cas9 genome editing of *Gria2* on C57BL/6N embryos. This line originated from a male F_0_ founder, which was backcrossed on a C57BL/6J background at the Plateforme In Vivo Exempt d’Organisme Pathogène Spécifique (PIV-EOPS) facility of the Interdisciplinary Institute for Neuroscience (IINS) to generate the AP-GluA2 KI mouse line used in this study (B6J-Gria2^em1(AP-TEV)Ics/Iins^ N2). Genotyping was performed by polymerase chain reaction (PCR) assay on tail biopsies by the genotyping facility of Neurocentre Magendie (Bordeaux Neurocampus). See also fig. S1. Primers used for genotyping KI and WT animals are shown in table S1. We used littermate or age-matched KI and WT control animals from the B6J-Gria2^em1(AP-TEV)Ics/Iins^ N2 line, as appropriate. The KI mutation did not affect animal weight, size, growth, or fertility. We used the SmithKline Beecham, Harwell, Imperial College, Royal London Hospital, phenotype assessment (SHIRPA) protocol (*74*, *75*) to assess the behavioral phenotype of AP-GluA2 KI mice and found no significant differences compared to WT littermates of 6- to 24-week-old mice (fig. S21). A group of WT C57BL/6J mice (origin The Jackson Laboratory) from the PIV-EOPS facility of the IINS was used for fear conditioning behavioral experiments to assess the impact of the KI and surgical manipulations. Animals were housed under a 12-hour light/12-hour dark cycle with unrestricted access to food and water. Dissociated hippocampal neuron cultures were prepared from postnatal day 0 (P0) male or female mice, and organotypic hippocampal slice cultures were prepared from P5 to P8 male or female mice. Histology, biochemistry, surgical manipulations, acute slice recordings, and behavior experiments were performed on 4- to 12-week-old male mice. All experiments were performed in accordance with the European guidelines for the care and use of laboratory animals, and the guidelines issued by the University of Bordeaux animal experimental committee (CE50; animal facilities authorizations A5012009, A3306940, and A33063941; ethical project authorizations 20778-2019021913051936 and 18507-201901118522837). Every effort was made to minimize the numbers and suffering of experimental animals.

### Molecular biology

The BirA^ER^ coding sequence was a gift from A. Ting (*29*). BirA^ER^ was cloned upstream (5′) of an encephalomyocarditis virus IRES sequence, and the eGFP reporter or Cre recombinase was cloned downstream (3′) of the IRES sequence such that the start codon of the BirA^ER^ signal sequence corresponded to the 11th ATG of the IRES sequence. An immunoglobulin K (IgK) leader sequence and hemagglutinin (HA) epitope tag was added to the 5′ end of BirA^ER^. BirA^ER^-eGFP, BirA^ER^-Cre, and eGFP constructs were cloned into the multiple cloning site of the pAAV vector (AAV_pSyn backbone). A CAG promoter was used in plasmids prepared for SCE, and a hSyn promoter was used in plasmids prepared for the synthesis of AAV particles. Plasmids were prepared using the ZymoPURE Plasmid MaxiPrep Kit (Zymo Research, ZD4203). All constructs were verified by restriction enzyme digestion and Sanger DNA sequencing. AAV serotype 1 and 9 preps were produced by the viral core facilities of the Bordeaux Neurocampus IMN, Charité Universitätsmedizin Berlin, or ordered from Addgene. pENN.AAV.hSyn.Cre.WPRE.hGH was a gift from J. M. Wilson (Addgene plasmid no. 105553; Addgene viral prep no. 105553-AAV9; https://www.addgene.org/105553; RRID:Addgene_105553). pAAV.synP.DIO.EGFP.WPRE.hGH was a gift from I. Wickersham (Addgene plasmid no. 100043; Addgene viral prep no. 100043-AAV9; https://www.addgene.org/100043; RRID:Addgene_100043). pAAV.Syn.Flex.GCaMP6f.WPRE.SV40 was a gift from D. Kim and GENIE Project (Addgene plasmid no. 100833; Addgene viral prep no. 100833-AAV9; http://n2t.net/addgene:100833; RRID:Addgene_100833) (*76*). Viral titers were between 2.40 × 10^13^ and 5.77 × 10^14^ genome-containing particles (GCP)/ml.

### Primary dissociated neuron cultures

Banker cultures of hippocampal neurons from P0 mice were prepared as previously described (*77*), with modifications. Briefly, pups were sacrificed by decapitation, and hippocampi were dissected and treated with papain for 20 min at 37°C and then dissociated in Hibernate-A medium (Gibco, A1247501). Dissociated neurons were plated at a density of 500,000 to 600,000 cells per 60-mm dish on poly-d-lysine and laminin (Sigma-Aldrich, P6407, 11243217001) precoated 18-mm 1.5H coverslips (Marienfeld-Superior, 0117580) in Neurobasal A medium supplemented with 10% heat-inactivated horse serum, 0.5 mM GlutaMAX, and B-27 Plus (Gibco, 26050088, 12349015, 35050061, and A3582801). After 30 min, coverslips were rinsed with Neurobasal A medium supplemented with 0.5 mM GlutaMAX and B-27 Plus and then flipped onto an astrocyte feeder layer. Neurons were maintained at 37°C with 5% CO_2_. Ara-C (2 μM) was added after 72 hours to stop glial proliferation. Astrocyte feeder layers were prepared 2 weeks in advance from P0 WT mice, plated at a density of 50,000 cells on poly-l-lysine (Sigma-Aldrich, P2636) precoated 60-mm dishes, and maintained at 37°C with 5% CO_2_ in minimum essential medium (MEM) supplemented with glucose (4.5 g/liter), 2 mM GlutaMAX, and 10% heat-inactivated horse serum (Gibco, 26050088). Cultured neurons were transduced with BirA^ER^-eGFP or eGFP AAV1 at 3 to 7 DIV by incubating coverslips overnight in 12-well plates with 0.5 ml of pre-conditioned Neurobasal A medium containing viruses at a multiplicity of infection (MOI) of 10,000 to 20,000. Coverslips were then returned to the 60-mm dishes and maintained with 10 μM d-biotin supplementation (Sigma-Aldrich, B4639) for 2 to 3 weeks, which was added when the medium was changed. Twenty percent to 30% of the medium was changed one to two times per week.

### Organotypic slice cultures

Organotypic hippocampal slice cultures from P5 to P8 mice were prepared as previously described (*78*), with modifications. Briefly, pups were anesthetized on ice and sacrificed by decapitation. Hippocampi were dissected in medium containing 10 mM d-glucose, 4 mM KCl, 26 mM NaHCO_3_, 234 mM sucrose, 5 mM MgCl_2_, 1 mM CaCl_2_, and 1 mM phenol red, equilibrated for ~5 min with carbogen (5% CO_2_ and 95% O_2_). Transverse slices (300 μm) were cut with a tissue chopper (WPI McIlwain) and then positioned on 0.45-μm Durapore membranes on Millicell culture inserts (Millipore, FHLC01300 and PICM0RG50) in six-well plates. Slices were maintained at 35°C with 5% CO_2_ in MEM containing 20% heat-inactivated horse serum, insulin (1 mg/liter), and 30 mM Hepes, 13 mM d-glucose, 5.2 mM NaHCO_3_, 1 mM l-glutamine, 0.25 mM ascorbate, 1 mM CaCl_2_, and 2 mM MgSO_4_ (Sigma-Aldrich, M4642 and I0516). The medium was replaced every 2 to 3 days.

Slices were transduced with BirA^ER^-eGFP, eGFP, BirA^ER^-Cre + FLEx eGFP, Cre + FLEx eGFP, or BirA^ER^-Cre + FLEx GCaMP6f AAV9 at 1 DIV by microinjection of the virus(es) (three to five pulses, 30 ms, 69 kPa) into the CA1 pyramidal cell layer with glass microelectrodes (~1 to 2 megaohms; Science Products, GB150F-10P) (*79*). BirA^ER^-eGFP, eGFP, and FLEx eGFP viruses were used at a dilution of 1:10 to 1:20 in 1× phosphate-buffered saline (PBS). BirA^ER^-Cre or Cre were used with FLEx eGFP or FLEx GCaMP6f at a dilution of 1:5000 to 1:20,000. Samples were maintained in medium on culture inserts and visualized under a stereomicroscope (Nikon SMZ 745T, Lumenera Infinity1). The microelectrode was positioned with a micromanipulator (Scientifica PatchStar), and viruses were injected using a Picospritzer (Parker Picospritzer III).

CA1 pyramidal neurons were electroporated at 3 DIV with glass microelectrodes (~4 to 6 megaohms) filled with an internal solution containing 135 mM K-gluconate, 0.2 mM EGTA, 10 mM Hepes, 4 mM MgCl_2_, 4 mM Na_2_–adenosine triphosphate (ATP), 0.4 mM Na–guanosine triphosphate (GTP), 10 mM Na_2_-phosphocreatine, 3 mM ascorbate (pH 7.2; 290 mOsm), and BirA^ER^-eGFP or eGFP plasmids (13 ng/μl) (*80*). Samples were maintained in prewarmed Tyrode’s solution containing 10 mM d-glucose, 10 mM Hepes, 120 mM NaCl, 3.5 mM KCl, 2 mM MgCl_2_, 2 mM CaCl_2_, 2 mM NaHCO_3_, and 1 mM Na-pyruvate (pH 7.3; 300 mOsm) on an upright microscope (Nikon Eclipse FN1, DS-Fi3); the microelectrode was positioned with a micromanipulator (Scientifica PatchStar); and cells were electroporated after the formation of a loose seal (4 × 25-ms pulses, 1 Hz, −2.5 V; NPI ISO-STIM 01D, Multi Channel Systems STG 4002, Voltcraft FPS-1132). After AAV transduction or SCE, slices were maintained in the culture medium supplemented with 10 μM biotin, which was added when the medium was changed.

### bAP-GluA2 labeling and TEV experiments

mSA was produced and conjugated to STAR 635P (Abberior, ST635P) using *N*-hydroxysuccinimide ester–activated fluorophore coupling as previously described (*30*). NA was conjugated to STAR 635P as above (Thermo Fisher Scientific, 31000), or NA-dye conjugates were purchased from commercial suppliers (Abberior STAR 635P, ST635P-0121; Invitrogen DyLight 550 or 633, 84606 and 22844). mSA and NA labeling experiments were performed on primary hippocampal cultures at 20 to 23 DIV or organotypic slices from 12 to 15 DIV, with the exception of the biotinylation time-course experiments, which were performed from 5 to 23 DIV.

For dissociated cultures, coverslips were washed twice for 5 min in Tyrode’s solution containing 10 mM d-glucose, 10 mM Hepes, 110 mM NaCl, 5 mM KCl, 5 mM MgCl_2_, and 2 mM CaCl_2_ (pH 7.4; ~260 mOsm) with 2% biotin-free bovine serum albumin (BSA) (Roth, 0163) and then incubated for 5 min with 100 nM NA and washed twice for 5 min with Tyrode-BSA. For TEV proteolytic cleavage, coverslips were washed in Tyrode’s solution without BSA, incubated for 10 min with 100 U of AcTEV (Invitrogen, 12575-015) or vehicle control, and then washed twice for 5 min in Tyrode’s solution. The above steps were performed at 37°C with 5% CO_2_. Cells were fixed for 10 min with 4% paraformaldehyde (PFA)–sucrose, washed three times with 1× PBS, incubated for 10 min with 50 mM NH_4_Cl, washed three times with 1× PBS, and then rinsed with H_2_O and mounted with Fluoromount-G + 4′,6-diamidino-2-phenylindole (DAPI) (Thermo Fisher Scientific, 00-4959-52).

For organotypic slices, samples were washed twice for 10 min in Tyrode’s solution containing 10 mM d-glucose, 10 mM Hepes, 120 mM NaCl, 3.5 mM KCl, 2 mM MgCl_2_, 2 mM CaCl_2_, 2 mM NaHCO_3_, and 1 mM Na-pyruvate (pH 7.3; 300 mOsm) with 1% biotin-free BSA and then incubated for 20 min in ~30 μl of Tyrode-BSA with 100 or 400 nM mSA or NA and washed twice for 10 min with Tyrode-BSA. For TEV proteolytic cleavage, slices were washed with Tyrode’s solution without BSA, incubated for 10 min with 100 U of AcTEV (Invitrogen, 12575-015) or vehicle control, and then washed twice for 5 min in Tyrode’s solution. For the AMPAR internalization assay, slices were incubated after mSA or NA labeling in Tyrode’s solution without BSA, with 1 μM TTX citrate (Tocris, 1069), or treated for 3 min with 30 μM NMDA (Sigma-Aldrich, M3262). After 30 min, slices were incubated for 10 min with AcTEV or vehicle control, as above. For biotinylation of AP-GluA2 by sBirA, slices were briefly washed with Tyrode’s solution and then incubated for 5, 15, 30, 60, or 90 min in ~30 μl of Tyrode’s solution with 10 μM biotin-AMP (Jena Bioscience, NU-894-BIO-S) and with or without 0.3 μM recombinant BirA (Sigma-Aldrich, SRP0417) (*28*). Slices were then incubated with NA as above. For two-color labeling, slices electroporated with BirA^ER^-eGFP at 3 DIV were first incubated with NA–DyLight 550 and then incubated for 30 min with 10 μM biotin-AMP and 0.3 μM recombinant BirA, followed by incubation with NA-STAR 635P. The above steps were performed at 35°C with 5% CO_2_. Slices were fixed and mounted as above, except fixation for slices was 2 hours at 4°C.

### Biochemistry and Western blots

Whole brain (minus cerebellum) and isolated hippocampal protein samples were prepared from 6-week-old mice. Tissue was homogenized in 5 ml of isomolar buffer containing 4 mM Hepes and 320 mM sucrose (pH 7.4), with Calbiochem protease and Halt phosphatase inhibitor cocktails (Millipore, 539134; Thermo Fisher Scientific, 78420). Subcellular fractionation was performed as described previously (*81*, *82*). All steps were performed at 4°C. For the TEV proteolytic cleavage assay, protein samples were incubated with AcTEV or vehicle control for 1 hour at 30°C. For the in vitro biotinylation assay, protein samples were incubated with 10 μM biotin-AMP with or without 0.3 μM recombinant BirA in 1× PBS with 5 mM MgCl_2_ (pH 8.0) for 1 hour at 30°C (*28*). For the deglycosylation assay, protein samples were incubated in glycoprotein denaturing buffer for 10 min at 100°C and then incubated for 2 hours at 37°C with 1% NP-40, with or without 500 U of Endoglycosidase H (endoH), 500 U of N-Glycosidase F (PNGase) F, or 40,000 U of *O*-glycosidase (New England Biolabs, P0702S, P0704S, and P0733S). Protein concentration was determined with a Pierce BCA assay (Thermo Fisher Scientific, 23225) before loading 4 to 15% SDS–polyacrylamide gel electrophoresis with the PageRuler plus prestained protein ladder (Thermo Fisher Scientific, 26619). Semidry transfers were done for 10 min with Trans-Blot Turbo HMW (Bio-Rad). Membranes were blocked for 1 hour with the Intercept Blocking Buffer (LI-COR, 927-70001) and immunoblotted overnight at 4°C with shaking in Intercept Blocking Buffer with the following antibodies: α-GluA2 (1 μg/ml; Millipore, MAB397 or Alomone, AGC-005), α-GluA1 (1 μg/ml; Neuromab, 75-327), α-PSD-95 (1 μg/ml; Neuromab, 75-028), α-γ8 (0.1 μg/ml; Frontier Institute, TARPgamma8-RbAf1000), α-stargazin (1 μg/ml; Cell Signaling Technology, 8511), α-CaMKII (1 μg/ml; Millipore, 05-532), α-β-actin (0.8 μg/ml; Sigma-Aldrich, 5316), α-synaptophysin (0.12 μg/ml; Abcam, 32127), and α-AP tag (1 μg/ml; Kerafast, EGO016). After three washes in a buffer containing 25 mM tris, 137 mM NaCl, 2.7 mM KCl, and 0.05% Tween 20, secondary antibodies conjugated to IRDye800CW or IRDye680LT (0.2 μg/ml; LI-COR, 926-68020, 926-68021, and 926-32211) or SA-IRDye800CW (0.2 μg/ml; LI-COR, 926-32230) were used for revelation for 1 hour at room temperature with shaking in the Intercept Blocking Buffer. Blots were imaged with Odyssey FC or CLx scanners, and band intensities were analyzed with Image Studio Lite version 5.2 (LI-COR). Line scan analysis was performed with Fiji ImageJ 1.53c [National Institutes of Health (NIH)]. Band intensities were normalized to loading controls, as indicated in the legends (figs. S14 to S17). Line scan values were normalized to minimum (background) values.

### Immunocytochemistry and immunohistochemistry

For live labeling of dissociated cultures at 20 to 23 DIV, coverslips were washed twice for 5 min in Tyrode’s solution with 1% biotin-free BSA and then incubated for 5 min with 15F1 α-GluA2 (1 μg/ml) and NA–DyLight 633 (100 nM) for live colabeling of the endogenous GluA2 epitope and the bAP tag or for 10 min with 15F1 α-GluA2 (2 μg/ml) for immunocytochemical characterization of GluA2. The 15F1 antibody was provided by E. Gouaux (*83*). Coverslips were then washed twice for 5 min with Tyrode-BSA and rinsed once with Tyrode’s solution without BSA. The above steps were performed at 37°C with 5% CO_2_. Cells were fixed for 10 min with 4% PFA-sucrose, washed three times with 1× PBS, incubated for 10 min with 50 mM NH_4_Cl, and then washed three times with 1× PBS. Blocking was done for 10 min in 1× PBS with 1% BSA, and then cells were incubated for 30 min with the secondary antibody (2 μg/ml; goat α-mouse Alexa Fluor 568, Thermo Fisher Scientific, A-21124) and washed three times with 1× PBS. For labeling of fixed cells, coverslips were fixed as above and then incubated for 10 min in 1× PBS with 1% BSA, with or without 0.1% Triton X-100 for permeabilization. Blocking was done as above, and coverslips were incubated for 30 min with 15F1 α-GluA2 (2 μg/ml), washed three times with 1× PBS, incubated for 30 min with goat α-mouse Alexa Fluor 568, and washed three times with 1× PBS. Coverslips were rinsed with H_2_O and mounted with Fluoromount-G + DAPI.

For immunohistochemistry, 9-week-old mice were anesthetized with ketamine/xylazine (130 and 13 mg/kg) before transcardial perfusion with 1× PBS and then 4% PFA. Brains were postfixed overnight in 4% PFA at 4°C, washed three times with 1× PBS, and incubated overnight with 30% sucrose in 1× PBS at 4°C. Frontal sections (50 μm) were cut in 1× PBS on a vibratome (Leica, VT1200). Floating sections were rinsed with 1× tris-buffered saline (TBS) and then permeabilized for 1 hour with 0.3% Triton X-100 and 5% normal goat serum (NGS) (Gibco, PCN5000). Slices were then incubated overnight with or without 15F1 α-GluA2 (1 μg/ml) in 1× TBS with 0.1% Triton X-100 and 5% NGS at 4°C. Slices were then washed three times for 10 min with 1× TBS, incubated for 2 hours with goat α-mouse Alexa Fluor 568 (1 μg/ml), and then washed three times with 1× TBS. Except for primary antibody incubation, the above steps were performed at room temperature with shaking. Slices were rinsed with H_2_O and mounted with Fluoromount-G + DAPI.

### Wide-field and confocal imaging

Wide-field imaging of cultured neurons and organotypic slice samples was done with a DM5000 (Leica) under HC PL Fluotar 5× numerical aperture 0.15, HC PL Fluotar 10× numerical aperture 0.3, HCX PL Fluotar 20× numerical aperture 0.5, or HCX PL Apo 63× oil numerical aperture 1.40 objectives (Leica). Fluorescence excitation was done with a light-emitting diode SOLA light (Lumencor), and emission was captured by an ORCA-Flash4.0 V2 camera (Hamamatsu) controlled by MetaMorph software (Molecular Devices). Mosaics were done with a motorized *XY* stage (Märzhäuser). Brain slices were imaged with a NanoZoomer 2.0-HT with fluorescence imaging module (Hamamatsu) using a UPS Apo 20× numerical aperture 0.75 objective combined to 1.75× lens (Nikon) for a final magnification of ×35. Fluorescence excitation was done with an LX2000 200-W mercury lamp, and emission was captured by a TDI-3CCD camera (Hamamatsu). Confocal imaging was done with a TCS SP8 or SP5 (Leica). The SP8 was mounted on a DM6 FS upright stand with HC Plan Fluotar 10× dry numerical aperture 0.30, HCX Plan Apo CS 20× multi-immersion numerical aperture 0.70, and HC Plan Apo CS2 63× oil numerical aperture 1.40 objectives (Leica). The SP8 was equipped with a motorized *XY* stage and a galvanometric *Z* stage; 405-, 488-, 552-, and 638-nm laser lines; a conventional scanner (10 to 1800 Hz); two internal photomultiplier tube (PMT) and two internal hybrid detectors for fluorescence detection; and one external PMT for transmission. The SP5 was mounted on a DM6000 upright stand with HCX Plan Apo CS 10× dry numerical aperture 0.40, HCX Plan Apo CS 20× multi-immersion numerical aperture 0.70, HCX Plan Apo CS 40× oil numerical aperture 1.25, and HCX Plan Apo CS 63× oil numerical aperture 1.40 (Leica). The SP5 was equipped with a motorized *XY* stage and a galvanometric *Z* stage; 405-, 488-, 561-, and 633-nm laser lines; a conventional scanner (10 to 2800 Hz); three internal PMT and two internal hybrid detectors for fluorescence detection; and one external PMT for transmission. Imaging parameters were kept the same among relevant samples. Image analysis was performed with ImageJ 1.53c or 1.53f51 (NIH). Except where stated in arbitrary units (AUs), fluorescence intensities were normalized by dividing by the mean value of a background ROI. Line scan values were normalized either to minimum (background) or maximum (peak) values. GFP intensity profiles (Fig. 1C and fig. S12, C and D) were uniformly shifted by +5 along the *y* axis to aid visualization where plotted together with mSA or NA intensity profiles. The synaptic enrichment factor (Fig. 3, D and E) was determined from line scan plot profiles drawn across spines and dendrites (Fig. 3C). For each line scan, the peak value of the spine was divided by the peak value of the corresponding dendritic shaft. Synaptic densities were calculated along a 50-μm length of dendrite from wide-field images (cell cultures) or confocal *z*-stack maximum intensity projections (organotypic slices).

### LLSM and photomanipulation

The LLSM (fig. S9) was built according to the technical information provided by the group of E. Betzig at Janelia Research Campus, Howard Hughes Medical Institute (HHMI), USA (*56*). The LLSM was used under license from Janelia Research Campus, HHMI. The lattice light sheet was focused by a custom 28.6× 0.66 numerical aperture 3.74-mm excitation objective (EO; Special Optics). Fluorescence was collected with a CFI Apo LWD 1.1 numerical aperture 25× 2.0-mm detection objective (DO; Nikon) and imaged on a scientific complementary metal-oxide semiconductor (sCMOS) ORCA-Flash4.0 V2 camera (Hamamatsu). The annular mask minimum and maximum numerical apertures were 0.44 and 0.55, respectively. We characterized the LLSM optical resolution using 170-nm-diameter beads and found values near diffraction limits laterally and better than confocal microscopy axially. Illumination power varied depending on the wavelength, sample brightness, labeling intensity, and depth in slice, which ranged from ~20 to 200 μW spread over the entire width and thickness of the LLSM excitation plane. Images were acquired at ~10 to 20 μm below the surface of the slice. Photobleaching during acquisitions was typically less than 20%. Fast sample translation with a piezo stage was used to acquire *Z* stacks. All images were acquired in dithered square lattice mode.

The LLSM setup was modified by addition of a PSM. Thirty percent of the laser combiner output was sent to the PSM by a variable beam splitter. For two-photon photostimulation, we also coupled a NIR fs laser beam (Coherent, Chameleon Vision-S) into the PSM path through a dichroic beam splitter (Thorlabs, DMLP 650). A mechanical shutter (Uniblitz, VS25) controlled the photostimulation duration. Submicrometer positioning and patterning were achieved by a set a galvanometric mirrors (XYT) (Cambridge Technology, 6215H) that were optically conjugated to the DO back focal plane. The photostimulation beam was magnified to fill the DO back aperture and coupled to the LLSM detection arm by a multiband dichroic mirror (Semrock, Di03-R405/488/561/635 for FRAP experiments and FF409/493/573/652/759-Di01 for two-photon uncaging experiments). Shutter timing and galvo positioning were controlled by a USB A/D card (National Instruments, 6002), programmed by a user interface written in LabVIEW (National Instruments). The photomanipulation beam diameter measured on beads was near diffraction limited (0.43 μm), and submicrometer diameter was maintained in the sample (0.69 μm). See also fig. S9. To ensure submicrometric targeting of user-defined ROIs, the PSM galvanometric mirrors were calibrated before every experimental session. Briefly, in a homogeneously fluorescent sample, a set of nine points corresponding to nine different galvo voltages pairs (*V_x_*,*V_y_*) was illuminated. Their positions were detected on LLSM camera images as nine pixels (*N_x_*,*N_y_*) for each voltage pair (*V_x_*,*V_y_*). An affine transformation matrix was computed to determine the correspondence between camera image pixels and PSM galvo voltages. A set of 10 randomly selected points was then targeted, and the distance between the targeted and detected spots was measured. The average of these 10 distances provided the mean targeting error, which was less than 0.2 μm for all experimental sessions.

### FRAP imaging and two-photon uncaging

For FRAP imaging, organotypic slices at 12 to 15 DIV that had been transduced with BirA^ER^-eGFP or BirA^ER^-Cre + FLEx eGFP AAV9 at 1 DIV were labeled with 100 nM NA or 400 nM mSA coupled to STAR 635P, as above. Membranes were cut and mounted onto poly-l-lysine precoated 5-mm coverslips and then mounted onto the LLSM sample holder with Dow Corning high-vacuum silicone grease (Sigma-Aldrich, Z273554). The imaging chamber was maintained at 34°C and perfused with ACSF containing 12.1 mM d-glucose, 126 mM NaCl, 2.5 mM KCl, 2 mM MgCl_2_, 2 mM CaCl_2_, 25 mM NaHCO_3_, and 1.25 mM NaH_2_PO_4_ (300 mOsm), which was equilibrated with carbogen and perfused at a rate of 1.7 ml/min. Slices were maintained in the imaging chamber for up to 2 hours. 3D images of spines and dendrites (typical volume of 12.8 μm by 12.8 μm by 20 μm) were acquired before FRAP imaging to identify the ROIs and labeling specificity, with 488-nm excitation of eGFP as a volume marker and BirA^ER^ reporter, and 642-nm excitation of mSA- or NA-STAR 635P as the extracellular label for bAP-GluA2. FRAP illumination was 50 ms to a single focal point, the power measured at the back aperture of the DO was below 4 mW, and the photobleach efficiency was typically ~40 to 80%. Fluorescence recovery was followed by single-plane acquisitions (100 ms) in three steps at 10 Hz (15 s), 1 Hz (60 s), and 0.2 Hz (180 s). Recovery curves were corrected for continuous photobleaching and background noise, as previously described (*18*, *84*, *85*). Image analysis was performed with ImageJ 1.53c or 1.53f51 (NIH). To analyze the recovery fraction, individual FRAP curves were fit to a nonlinear regression model using one-phase association with a constraint of *Y*_0_ = 0 (Prism 8.3.1, GraphPad). FRAP curves (68 to 88%) were fit successfully and included in the analysis.

For two-photon glutamate uncaging, organotypic slices at 12 to 17 DIV that had been transduced with BirA^ER^-Cre + FLEx GCaMP6f AAV9 at 1 DIV were labeled with 100 nM NA coupled to STAR 635P and then mounted in the LLSM imaging chamber as above. ACSF containing 2.5 mM MNI-glutamate (Hello Bio, HB0423), 0.0005 mM TTX, 12.1 mM d-glucose, 126 mM NaCl, 2.5 mM KCl, 1.3 mM MgCl_2_, 2.5 mM CaCl_2_, 25 mM NaHCO_3_, and 1.25 mM NaH_2_PO_4_ (300 mOsm) was equilibrated with carbogen and perfused at a rate of 1.7 ml/min. 3D images of dendritic branches (typical volume of 25.6 μm by 25.6 μm by 40 μm) were acquired before uncaging acquisitions for quantification of synaptic bAP-GluA2 content, with 488-nm excitation of GCaMP6f as a volume marker and BirA^ER^ reporter, and 642-nm excitation of NA-STAR 635P as the extracellular label for bAP-GluA2. Illumination power (~100 μW across the LLSM excitation plane) was kept consistent across experiments. Two-photon glutamate uncaging at 720 nm (2 ms) was done at a focal point ~1 μm from the spine head. Synaptic Ca^2+^ transients were followed by single-plane acquisitions (5 ms) for 1.25 s at 200 Hz. Response amplitudes were calculated as Δ*F*/*F*_0_ = ((*F*_peak_ − *F*_bkgd_) − (*F*_bsln_ − *F*_bkgd_))/(*F*_bsln_ − *F*_bkgd_), and up to five trials were averaged per spine. Acquisitions where glutamate uncaging induced dendritic Ca^2+^ spikes were excluded from analyses. GCaMP6f response analysis was performed with ImageJ 1.53f51 (NIH). 3D quantification of NA intensity over the surface of dendritic spines and spine volume were analyzed using the Surfaces module in Imaris 9.8.2 (Oxford Instruments).

### Superresolution imaging

For dSTORM imaging, primary neuronal cultures at 21 DIV were live-labeled with 15F1 α-GluA2 (3.33 μg/ml) for 7 min at 37°C and then fixed as above. Cells were incubated for 30 min with the secondary antibody (2 μg/ml; goat α-mouse Alexa Fluor 647, Thermo Fisher Scientific, A-21235). dSTORM imaging was performed on Leica DMi8 mounted on an antivibrational table (TMC) using an HCX PL Apo 160× 1.43 numerical aperture (NA) oil immersion total internal reflection fluorescence (TIRF) objective (Leica) and fiber-coupled laser launch (405, 488, 532, 561, and 642 nm) (Roper Scientific). Fluorescence was collected with an Evolve electron-multiplying charge-coupled device (EMCCD) camera (Photometrics). Coverslips were mounted on an open Ludin chamber (Life Imaging Services), and 600 μl of imaging buffer was added (*38*). To reduce oxygen exchange during acquisition, an 18-mm coverslip was placed on top of the chamber. Image acquisition was driven by MetaMorph software (Molecular Devices), and one image stack contained 40,000 frames. The size of the region acquired was 512 pixels by 512 pixels (100 nm per pixel). Keeping the 642 laser intensity constant, the power of the 405-nm laser was increased during acquisition to control the level of single molecules per frame. Multicolor fluorescent microspheres (TetraSpeck, Invitrogen) were used as fiducial markers to register long-term acquisitions and correct for lateral drifts. Intensity-based drift-corrected superresolution images (25 nm per pixel) were reconstructed using PALM-Tracer software operating as a plugin of MetaMorph (*86*). The synaptic enrichment factor (Fig. 4D) was determined from ROIs drawn manually around spine heads and extrasynaptic regions on the dendrite in close proximity to the spine. For each ROI, the integrated intensity was normalized to the area, and the value of the synaptic ROI was divided by the value of the corresponding extrasynaptic ROI.

For uPAINT imaging, the microscope was caged and heated to maintain samples at 37°C. Coverslips were mounted on an open Ludin chamber (Life Imaging Services) and maintained in preequilibrated Tyrode’s solution containing 12.5 mM d-glucose, 25 mM Hepes, 108 mM NaCl, 5 mM KCl, 2 mM MgCl_2_, and 2 mM CaCl_2_ (pH 7.4; ~260 mOsm). Imaging was performed on a Ti-Eclipse microscope equipped with an Apo 100× 1.49 NA oil immersion TIRF objective (Nikon) and laser diodes (405, 488, 561, and 642 nm) (Roper Scientific). Fluorescence signal was detected with an Evolve EMCCD camera (Photometrics). To compare diffusion dynamics, KI and WT neurons transduced with BirA^ER^-eGFP or eGFP AAV1 were imaged at 16 to 21 DIV. eGFP^+^ KI or WT neurons were labeled with a low concentration (~1 nM) of 15F1 α-GluA2 coupled to SeTau 647 (SETA BioMedicals), which was added to the Ludin chamber to sparsely and stochastically label endogenous surface GluA2. For cross-link experiments, KI neurons were washed three times with preequilibrated Tyrode’s solution, followed by incubation with Tyrode’s solution (control) or 100 nM unconjugated NA for 8 min at 37°C, and then washed again. After mounting the sample, eGFP^+^ neurons were labeled with a low concentration of mSA-STAR 635P (7.7 nM) to sparsely and stochastically label surface bAP-GluA2. To avoid phototoxicity, the 642-nm laser was activated at low intensity. The TIRF angle was adjusted to maximize signal-to-noise ratio. Image acquisition and control of the microscope were driven by MetaMorph (Molecular Devices), the acquisition time was 30 ms, and 6000 to 8000 frames were acquired to record GluA2 lateral diffusion. The diffusion coefficient (*D*) based on the fit of the mean square displacement curve was extracted from the experiments and analyzed with WaveTracer software operating as a plugin of MetaMorph (*86*).

### Transmission electron microscopy

For TEM imaging, KI organotypic slices transduced with BirA^ER^-eGFP or BirA^ER^-Cre + FLEx eGFP AAV9 were live-labeled at 15 DIV in Tyrode’s solution with SA-FNG, conjugated to Alexa Fluor 546 or Alexa Fluor 594 and 1.4-nm NanoGold particles (SA-FNG, Nanoprobes Inc.). Slices were fixed with 4% PFA-sucrose and 0.2% glutaraldehyde in 1× PBS overnight at 4°C. To confirm SA-FNG staining, slices were imaged in 1× PBS with an SP5 confocal under an HCX IRAPO L 25X water NA 0.95 objective, as above (Leica). SA-FNG and eGFP^+^ regions were dissected under a stereomicroscope equipped with a NightSea fluorescence system (EMS). The dissected tissue was then incubated in a permeabilizing buffer containing 0.1% Triton X-100 and 0.2% gelatin in 1× PBS and then reincubated with SA-FNG in permeabilizing buffer to facilitate synaptic labeling. Samples were then subjected to silver enhancement for 5 min (HQ Silver, Nanoprobes Inc.) to detect silver-enhanced nanogold particles by conventional electron microscopy. Samples were postfixed with 1% glutaraldehyde in 0.15 M Sorensen’s phosphate buffer (SPB; EMS) for 10 min at 4°C and then incubated with 1% sodium thiosulfate in H_2_O, washed with SPB, and fixed with SPB containing 1% OsO_4_ and 1.5% potassium ferrocyanide for 1 hour on ice. Sequential dehydration was performed with 70, 90, and 100% ethanol, followed by two incubations with 100% acetone on ice, and then samples were embedded for 2 hours with 1:1 acetone and epon resin (Embed-812, EMS), followed by 100% epon resin at 60°C for 2 days. TEM imaging was performed in high-contrast mode with an H7650 transmission electron microscope (Hitachi) equipped with an Orius SC1000 CCD camera (Gatan Inc.).

### Stereotaxic surgery and cannula implantation

Mice were positioned in a stereotaxic apparatus (David Kopf Instruments) under continuous isoflurane anesthesia with a vaporizer system and treated with intraperitoneal buprenorphine (0.1 mg/kg) and local lidocaine (0.4 ml/kg; 1% solution). Stereotaxic injections of BirA^ER^-eGFP or eGFP AAV9 were performed on AP-GluA2 KI males aged 4 to 6 weeks for slice electrophysiology or aged 6 to 8 weeks when coupled with cannula implantation for behavioral experiments. The CA1 region was targeted for AAV9 injection with the coordinates Anterior Posterior (AP) −2 mm, Medial – Lateral (ML) ±1.6 mm, and Dorsal Ventral (DV) −1.15 mm. Stainless steel guide cannulae (26 gauge; PlasticsOne) were bilaterally implanted above the hippocampus with the coordinates AP −1.6 to 2.0 mm, ML ±1.9 to 2.3 mm, and DV −0.3 mm. Guide cannulae were anchored to the skull with dental cement (Super-Bond, Sun Medical Co.). After surgery, mice recovered from anesthesia on a heating pad (35°C), and dummy cannulae were inserted into the guides to reduce the risk of infection. To ensure that AP-GluA2 were maximally biotinylated for patch and field LTP experiments in acute slices, 100 μl of biotin at 6 mg/ml (pH 7.4) was injected intraperitoneally daily for 5 days before slicing. To avoid confounding behavioral experiments by stress induced by daily intraperitoneal injections, no biotin supplementation was performed for fear conditioning experiments.

### Acute slice preparation and electrophysiological recordings

Acute slices were prepared 2 to 3 weeks following AAV9 stereotaxic injection, and AP-GluA2 KI and WT mice (7 to 12 weeks) were anesthetized with isoflurane before decapitation. For virus injections, cohorts of five and five animals were done blind (BirA^ER^-eGFP or eGFP control). Animal numbers for all groups are ≥5, with the exception of the eGFP group used for LTP experiments (*N* = 3), where two animals were excluded due to mistargeting of the stereotaxic AAV injection site. Slice numbers are indicated in the figure/legends. Brains were removed, and hippocampal parasagittal slices (320 to 350 μm) were prepared with a vibratome (Leica, VT1200s) in an ice-cold solution containing 2 mM KCl, 26 mM NaHCO_3_, 1.15 mM NaH_2_PO_4_, 10 mM glucose, 220 mM sucrose, 0.2 mM CaCl_2_, and 6 mM MgCl_2_ (pH 7.4; 290 to 310 mOsm), oxygenated with carbogen. Slices were incubated for 30 to 45 min at 35°C and then maintained at room temperature before being transferred to the recording chamber perfused at a rate of 1.8 ml/min with ACSF containing 119 mM NaCl, 2.5 mM KCl, 26 mM NaHCO_3_, 1 mM NaH_2_PO_4_, 10 mM glucose, 2.5 mM CaCl_2_, and 1.3 mM MgCl_2_ (300 mOsm), oxygenated with carbogen.

Whole-cell patch-clamp recordings were made from CA1 pyramidal cells with a borosilicate glass pipette (4 to 6 megaohms) filled with an internal solution containing 125 mM CsMeSO_3_, 2 mM MgCl_2_, 1 mM CaCl_2_, 10 mM EGTA, 10 mM Hepes, 4 mM Na_2_-ATP, 0.4 mM Na-GTP, and 5 mM QX-314-Cl. Spermine (100 mM) was added to the internal solution to quantify the rectification index of AMPA-mediated EPSCs. Spontaneous EPSCs and IPSCs were recorded at a holding potential of −70 and 0 mV, respectively. Spontaneous currents at each potential were averaged, and the rise time (20 to 80%; milliseconds) and decay time constant, determined with a monoexponential fit, were computed. Synaptic responses in CA1 pyramidal cells were elicited by a brief electrical stimulation (0.1 ms, 0.5 to 2 V) of the Schaffer collaterals with a bipolar electrode placed in the stratum radiatum of CA1. For the rectification index, ACSF contained picrotoxin (100 μM) and D-AP5 (25 μM) to block inhibitory and NMDA-mediated synaptic responses. Ten minutes after entering into whole cell, AMPA-mediated synaptic responses were recorded at different holding potentials ranging from −70 to +40 mV. A linear fit was performed to compute the theoretical EPSC amplitude at +40 mV. The rectification index is the ratio between the theoretical and experimental value. The NMDA/AMPA ratio was determined in the presence of picrotoxin (100 μM) in the ACSF. The amplitude of the NMDA component 50 ms after the stimulation onset recorded at +50 mV was divided by the amplitude of the AMPA-mediated EPSCs recorded at −70 mV. A monoexponential fit was performed on the EPSC recorded at +50 mV to determine the NMDA EPSC decay (milliseconds). For the excitatory/inhibitory ratio (*E*/*I*), synaptic inhibitory responses were recorded at 0 mV, which is the reversal potential for AMPA and NMDA synaptic currents. Synaptic current onsets were determined by using the Onset function from NeuroMatic v3.0c (*87*) operating as a plugin of Igor Pro 8.04 (WaveMetrics). Voltage signals were recorded using a MultiClamp 700B amplifier with Clampex 10.7 (Molecular Devices), low pass–filtered at 10 kHz, digitized at 10 kHz, and stored on a PC for analysis.

For extracellular field recordings, electrical stimulation of Schaffer collaterals and recordings of synaptic responses were made in the CA1 stratum radiatum with borosilicate glass pipettes (tip diameter of 5 to 10 μm; resistance of 0.2 to 0.3 megaohms) filled with ACSF. For *I*/*O* curves, Schaffer collaterals in the CA1 region were gradually stimulated (0.5 ms, 0 to 100 μA, 0.1 Hz) and recorded in the stratum radiatum of CA1. For LTP recordings, a 10-min baseline of synaptic responses elicited by stimulation at 0.1 Hz was recorded in the current clamp mode, and HFS (3× 100 Hz, 1 min) was applied to induce LTP. Synaptic responses at 0.1 Hz were recorded for a further 40 min to observe LTP. For cross-linking experiments, slices were preincubated with 100 nM NA in ACSF for 30 to 60 min. Recordings were then performed in ACSF with 10 pM NA to immobilize newly exocytosed bAP-GluA2 (*25*). The FV and fEPSP slopes were measured and compared to the stimulation intensity. For LTP recordings, the FV and fEPSP responses were normalized to baseline by calculating their slopes before and after HFS, and the normalized ratio is presented. Signals were recorded as above, and analysis was performed with Clampfit 10.7 (Molecular Devices) and SigmaPlot V14 (Systat Software Inc.).

### Fear conditioning

Mice were housed individually in a ventilation area before the start of behavioral training. Two weeks after stereotaxic surgery, the mice were handled for an additional week. To reduce stress of the mice during subsequent experiments, they were trained daily for multiple insertions and removals of dummy cannula. On the first experimental day (day 1), animals were allowed to explore the conditioning context (context A) for habituation. Both CS+ (30-s duration, consisting of 50-ms tones repeated at 0.9 Hz, tone frequency of 7.5 kHz, 80-dB sound pressure level) and CS− (30-s duration, consisting of 50-ms white noise tones repeated at 0.9 Hz, 80-dB sound pressure level) were presented four times with a variable interstimulus interval (ISI; 10 to 30 s). On day 2, infusion cannulae (33-gauge) connected to a 1-μl Hamilton syringe via polyethylene tubing were inserted into the guides, extending beyond the end of the guide cannulae by 2 mm to target the CA1 of the hippocampus. Texas Red–conjugated NA (8.33 μM; Invitrogen, A2665) or saline was infused bilaterally at a rate of 50 nl/min for a total volume of 500 nl per hemisphere, under the control of an automatic pump (Legato 100, KD Scientific Inc.). The injector was maintained for an additional 2 min to allow sufficient diffusion. NA or saline was injected just before presentations of CS/US pairs in context A. The conditioning phase was performed as follows: five pairings of CS+ with the US onset coinciding with the CS+ offset (2-s foot shock, 0.6 mA). In all cases, CS− presentations were intermingled with CS+ presentations, and ISI was variable over the whole training course. Contextual memory was tested 48 hours after conditioning by analyzing the freezing levels during 5 min of context A reexposure. Cued fear memory was tested 48 hours after conditioning by analyzing the freezing levels during the first 120 and 30 s following the 12 CS+ presentations in context B. Freezing behavior was recorded in each behavioral session using a FireWire CCD camera (Ugo Basile) connected to an automated freezing detection software (ANY-maze, Stoelting Co.). Measurements of freezing behavior were done blind and in sessions alternating between experimental groups. Mice within each group were also selected randomly for analysis. At the experimental end point, mice were infused with 500 nl of NA and then anesthetized before transcardial perfusion, as above. Frontal brain sections (60 μm) were cut on a vibratome (Leica, VT1200) for postmortem verification of bilateral hippocampal AAV9 expression, NA binding, and implanted cannula position.

### Statistical analysis

All reported results are derived from at least three independent experiments to ensure replicability. No statistical test was used to predetermine sample sizes. For imaging data, analysis was performed blinded to the experimental condition or done with a semiautomated macro to minimize user bias. For electrophysiology and animal behavior experiments, the experimenter was blind to the genotype or AAV identity. Statistical analysis and data plotting was performed with Prism 8.3.1 or 9.1.1 (GraphPad) or SigmaPlot (V14). Datasets were analyzed with the Shapiro-Wilk test for normality, and parametric (*P* > 0.05) or nonparametric (*P* < 0.05) statistical tests (two-tailed) were performed as appropriate. *F* test or Bartlett’s test was used to assess equality of variance. Fear conditioning behavioral data were analyzed with the robust regression and outlier removal (ROUT) method to identify outliers from nonlinear regression, and one outlier was removed from the BirA^ER^ + NA test group. Notably, the one outlier in this test group exhibited lower eGFP fluorescence intensity, absence of NA binding upon terminal NA infusion, and a more lateral position of the implanted cannula, suggesting that the lack of effect on fear behavior was due to a lack of AMPAR cross-linking in the CA1 region. Test details and statistical outcomes for all experiments are reported in the relevant figure legends.

## Supplementary Materials

### This PDF file includes:

Table S1 Figs. S1 to S22 Key Resources Table
