# Supplementary material for: High-resolution imaging and manipulation of endogenous AMPA receptor surface mobility during synaptic plasticity and learning
Source: Sci Adv. 2022 Jul 27;8(30):eabm5298. doi: 10.1126/sciadv.abm5298 (PMC9328687; doi:10.1126/sciadv.abm5298)
Supplement: Supplementary file 1 — Table S1 Figs. S1 to S22 Key Resources Table [file sciadv.abm5298_sm.pdf]

Science Advances
AAAS

Supplementary Materials for

High-resolution imaging and manipulation of endogenous AMPA receptor surface mobility during synaptic plasticity and learning

Angela M. Getz *et al.*

Corresponding author: Daniel Choquet, daniel.choquet@u-bordeaux.fr

Sci. Adv. **8**, eabm5298 (2022)
DOI: 10.1126/sciadv.abm5298

This PDF file includes:

Table S1
Figs. S1 to S22
Key Resources Table

SUPPLEMENTARY EXPERIMENTAL PROCEDURES

Table S1: AP-GluA2 KI PCR Genotyping Strategy

AP-GluA2 Genotyping Primers sequences	KI Amplicon	WT Amplicon
5’ GGAAAAGGAGGAAAGGGAAACGAGG	494 bp	350 bp
3’ CCTTTCCCCTCTATTGTGTCAAAAG		

SUPPLEMENTARY FIGURES AND FIGURE LEGENDS

Supplementary Figure S1: CRISPR-Cas9 strategy for AP-TEV knock-in on *Gria2*

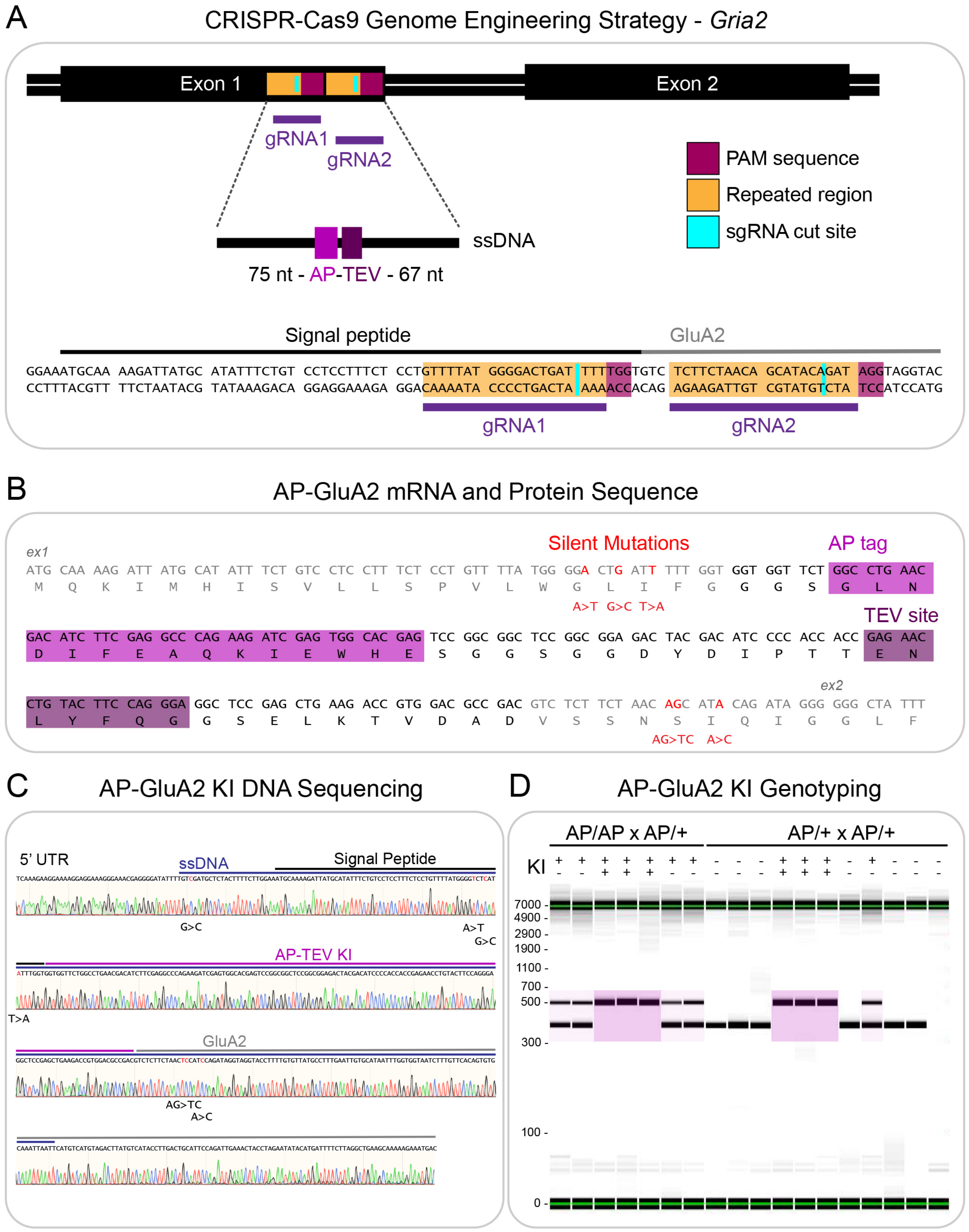

Supplementary Figure S1: CRISPR-Cas9 strategy for AP-TEV knock-in on *Gria2*

(**A**). Overview of the genome editing approach to modify *Gria2* by CRISPR-Cas9 (*43*), with a single stranded DNA donor template carrying the AP-TEV sequence targeted by two guide RNAs to PAM sequences in exon 1. (**B**). Modification of the N-terminal GluA2 mRNA/protein sequence by AP-TEV knock-in, including six silent mutations (three per gRNA target) encoded on the ssDNA template to avoid re-cutting after homology directed repair. Endogenous *Gria2* coding sequence is shown in grey, knock-in sequence is shown in black, and silent mutations are shown in red. The biotin acceptor peptide tag (AP) sequence is highlighted pink, and the tobacco etch virus (TEV) protease consensus sequence is highlighted purple. (**C**). Sanger sequencing of the male F0 founder. One unexpected silent mutation (G>C) was found in the 5' UTR. (**D**). Example of PCR genotyping results obtained from homozygous or heterozygous KI and WT animals using capillary electrophoresis (LabChip GX). Sequencing primers (see Table S1) produce an amplicon of 494 bases from AP-TEV KI *Gria2*, highlighted in pink, and 350 bases from WT *Gria2*.

Supplementary Figure S2: TEV protease cleaves the AP tag and reverses biotinylated AP-GluA2 surface labeling

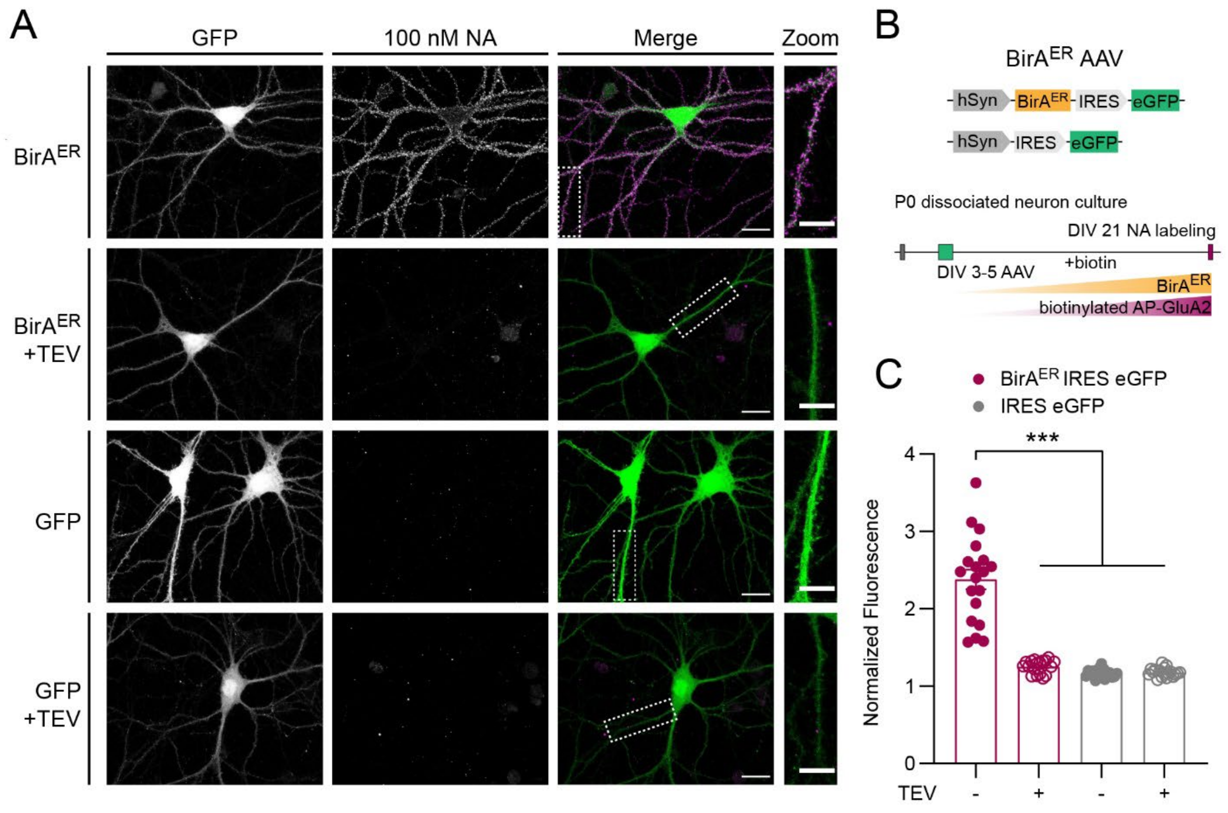

Supplementary Figure S2: TEV protease cleaves the AP tag and removes the biotinylated AP-GluA2 surface label

(**A-B**). Primary hippocampal neuron cultures from AP-GluA2 KI mice were transduced with AAV1 encoding BirA^{ER}-eGFP or eGFP control and maintained for 21 days in media supplemented with 10 μM biotin. Cells were incubated with 100 nM NA conjugated to DyLight 633 to label surface localized bAP-GluA2, then incubated with a control buffer or 100 U TEV protease to cleave the AP tag and remove the NA surface label. (**A**). Representative confocal images of neurons transduced with BirA^{ER}-eGFP or eGFP control AAV1, incubated with 100 nM NA, then with or without TEV. NA labeling is specific to AP-GluA2 KI with BirA^{ER} and without TEV. Scale bars, 20 and 10 μm. (**B**). Schematic representation of the experiment. (**C**). Normalized fluorescence intensity of NA-DyLight 633, coincident with the eGFP reporter. N≥17. ***P≤0.0007 (Kruskal-Wallis test; F=47.18, P<0.0001; Dunn's post-hoc test). Error Bars, SEM.

Supplementary Figure S3: Time course of biotinylated AP-GluA2 expression with AAV-mediated expression of BirA^{ER}

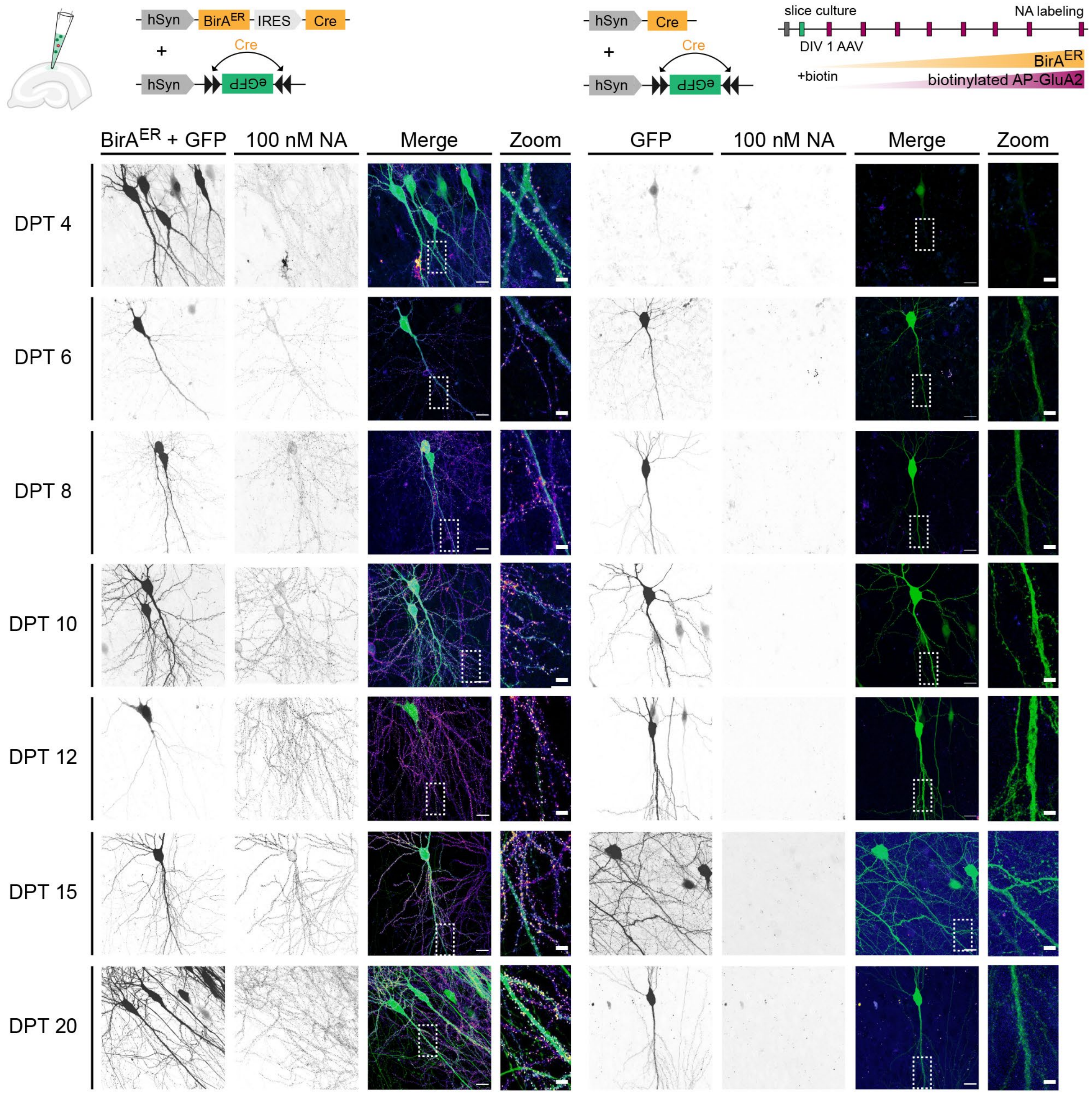

Supplementary Figure S3: Time course of biotinylated AP-GluA2 expression with AAV-mediated expression of BirA^{ER}

Organotypic slice cultures from AP-GluA2 KI mice were transduced with AAV9 encoding BirA^{ER}-Cre + FLEx eGFP or Cre + FLEx eGFP control in CA1 and maintained for 5-21 days in media supplemented with 10 µM biotin. Slices were incubated with 100 nM NA conjugated to DyLight 633 to label surface localized bAP-GluA2. Representative confocal images of CA1 pyramidal neurons in organotypic hippocampal slices transduced with BirA^{ER}-Cre + FLEx eGFP (left) or Cre + FLEx eGFP control (right). DPT, days post transduction. Scale bars, 20 and 5 µm. Schematic representation of the experiment is shown above. N≥3. Normalized fluorescence intensity of NA-DyLight 633, coincident with the eGFP reporter, is shown in Figure 1E.

Supplementary Figure S4: Time course of biotinylated AP-GluA2 expression with plasmid-mediated expression of BirA^{ER}

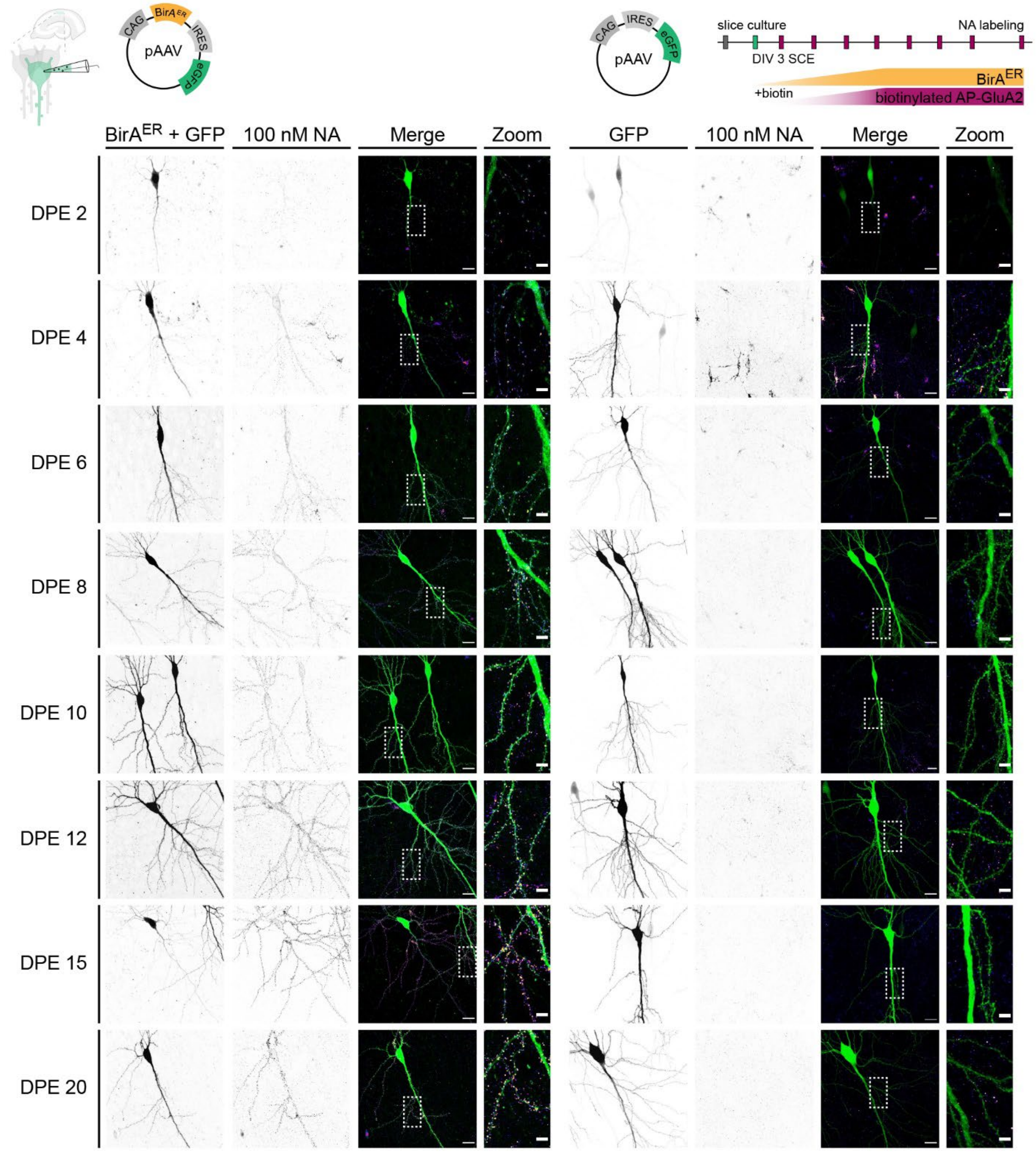

Supplementary Figure S4: Time course of biotinylated AP-GluA2 expression with plasmid-mediated expression of $BirA^{ER}$

CA1 pyramidal neurons in organotypic slice cultures from AP-GluA2 KI mice were electroporated with 13 ng/uL pAAV plasmid encoding $BirA^{ER}$-eGFP or eGFP control and maintained for 5-23 days in media supplemented with 10 µM biotin. Slices were incubated with 100 nM NA conjugated to DyLight 633 to label surface localized bAP-GluA2. Representative confocal images of CA1 pyramidal neurons in organotypic hippocampal slices electroporated with $BirA^{ER}$-eGFP (left) or eGFP control (right). DPE, days post electroporation. Scale bars, 20 and 5 µm. Schematic representation of the experiment is shown above. N≥5. Normalized fluorescence intensity of NA-DyLight 633, coincident with the eGFP reporter, is shown in Figure 1G.

Supplementary Figure S5: Normal spine density on CA1 pyramidal neurons expressing BirA^{ER} in AP-GluA2 KI

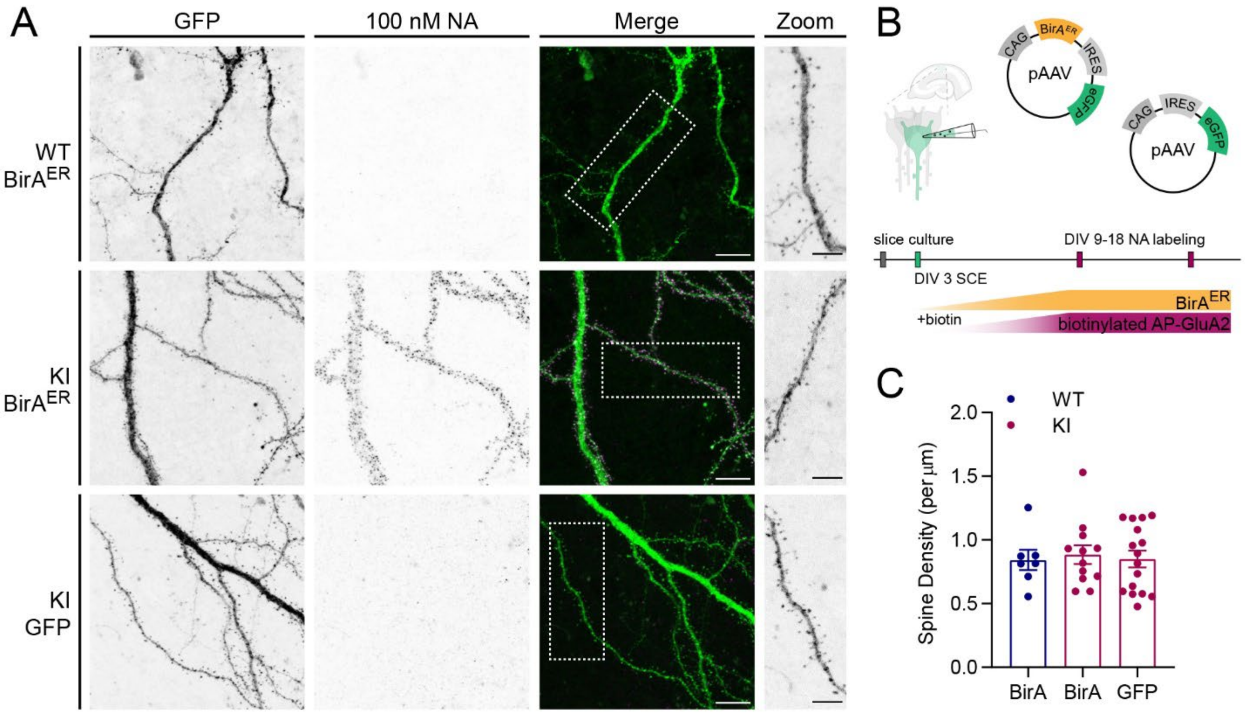

Supplementary Figure S5: Normal spine density in CA1 pyramidal neurons expressing BirA^{ER} in AP-GluA2 KI

(**A**). Representative confocal images (z-projections) of electroporated CA1 pyramidal neurons in organotypic hippocampal slices used to count spine density. Scale bars, 10 and 5 µm. CA1 pyramidal neurons in organotypic slice cultures from WT or AP-GluA2 KI mice were electroporated with 13 ng/uL pAAV plasmid encoding BirA^{ER}-eGFP or eGFP control and maintained for 9 or 18 days in media supplemented with 10 µM biotin. Slices were incubated with 100 nM NA conjugated to DyLight 633 to label surface localized bAP-GluA2. (**B**). Schematic representation of the experiment. (**C**). Spine density counts of CA1 neurons from WT + BirA^{ER}, KI + BirA^{ER} and KI + eGFP conditions, measured along a 50 µm length of dendrite in the *stratum radiatum*. N≥7. $P \geq 0.9302$ (one-way ANOVA; F=0.0868, P=0.9171; Tukey's post-hoc test). Error bars, SEM.

Supplementary Figure S6: Time course of AP-GluA2 biotinylation with soluble BirA incubation

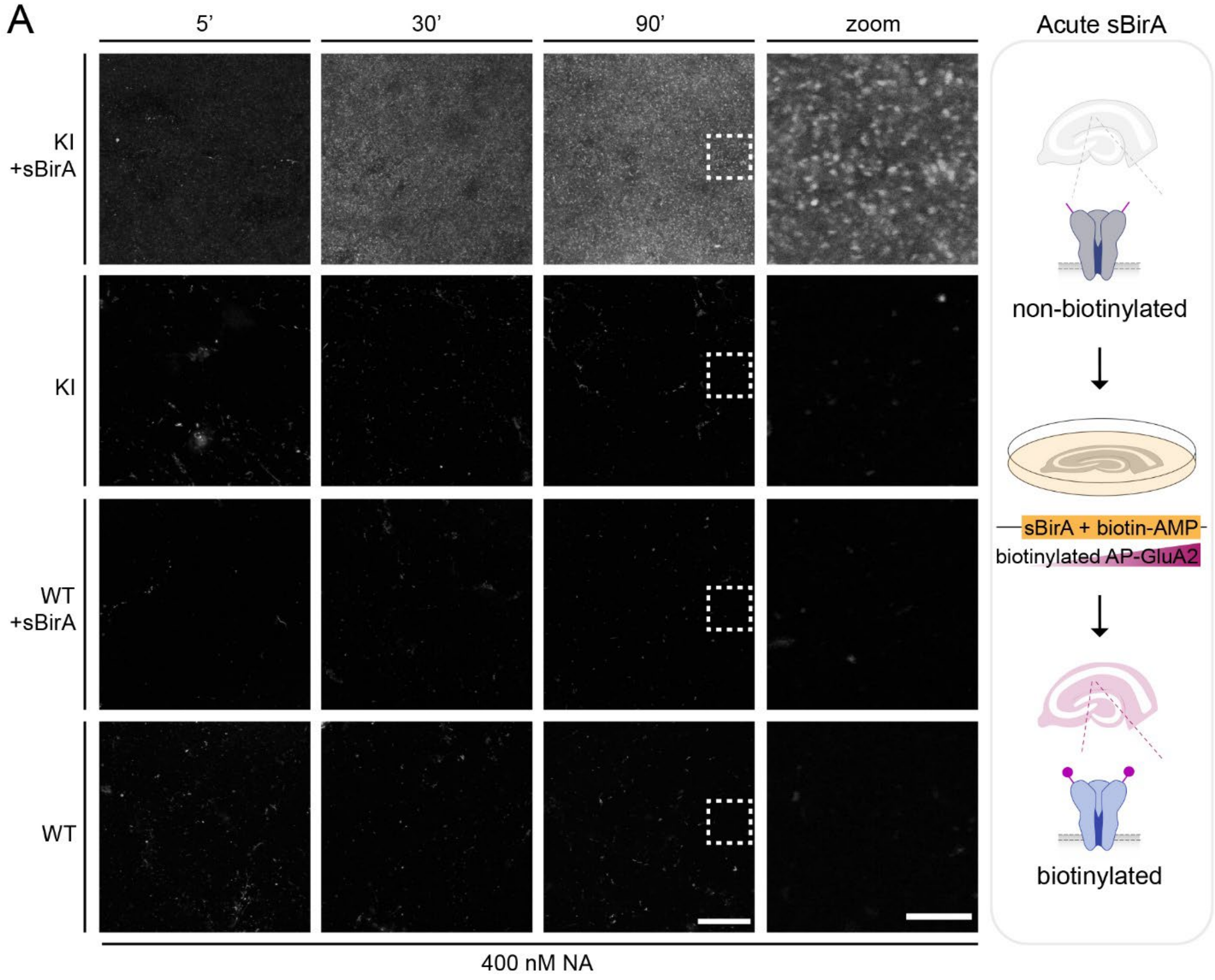

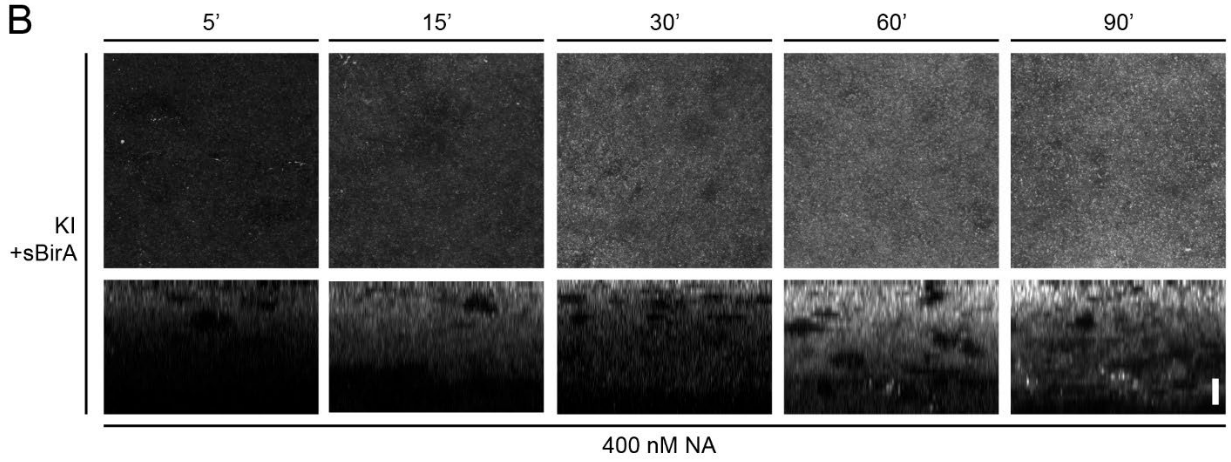

Supplementary Figure S6: Time course of AP-GluA2 biotinylation with soluble BirA incubation

(**A**). Organotypic slice cultures from AP-GluA2 KI or WT mice were incubated with 10 μM biotin-AMP and with or without 0.3 μM sBirA for 5, 30, or 90 min, then incubated with 400 nM NA conjugated to DyLight 633 to label surface localized bAP-GluA2. Representative confocal images from the *stratum radiatum* in organotypic hippocampal slices, as above. Scale bars, 20 and 5 μm. Schematic representation of the experiment is shown on the right. Normalized fluorescence intensity of NA-DyLight 633 is shown in Figure 1I. (**B**). Orthogonal projections (Y-Z) of NA-DyLight 633 labeled biotinylated AP-GluA2 after 5-90 min incubation with biotin-AMP and sBirA demonstrate progressive biotinylation of AP-GluA2 in depth over time. Scale bar, 10 μm.

Supplementary Figure S7: Specificity of mSA labeling of biotinylated AP-GluA2 in organotypic hippocampal slice cultures

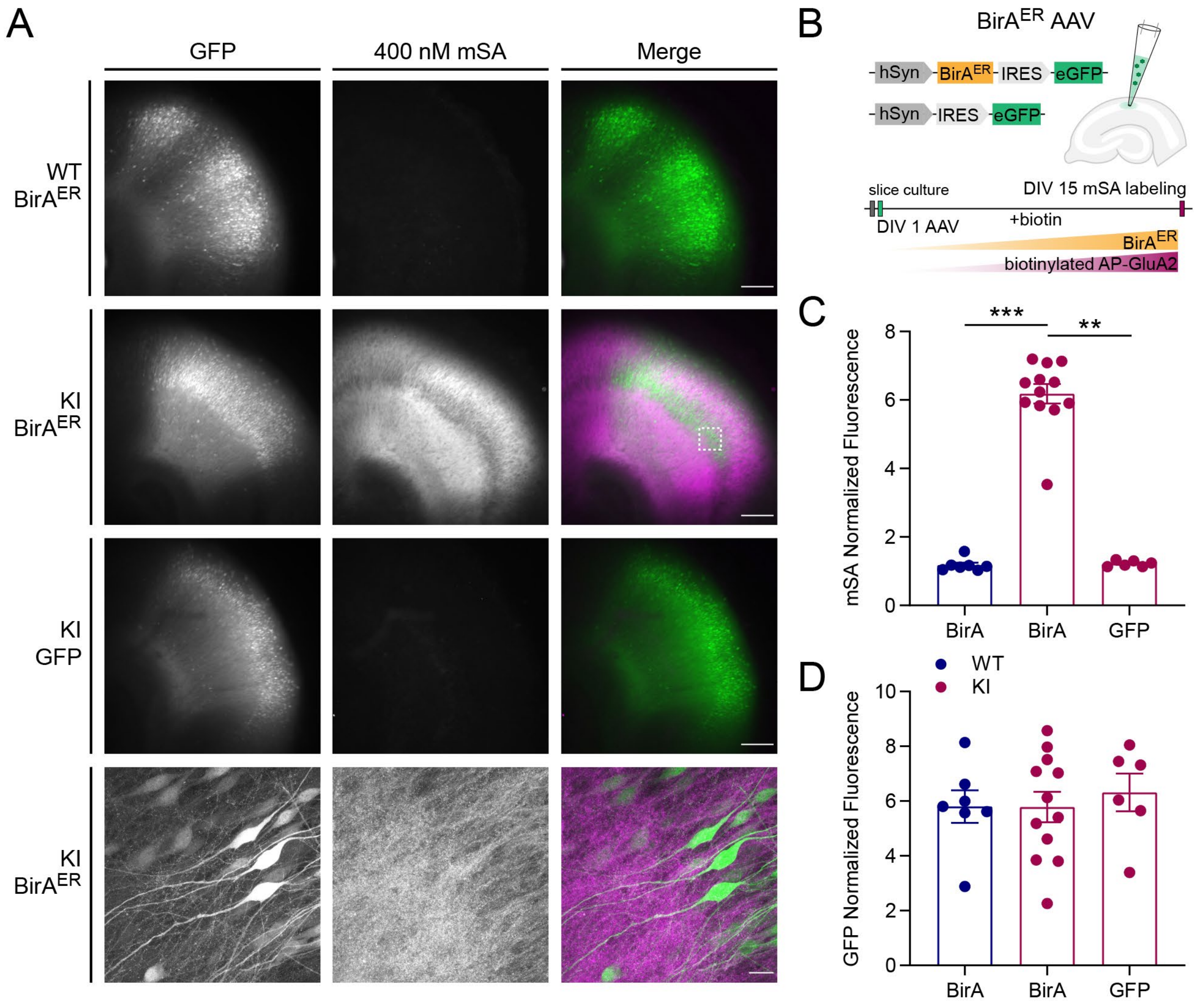

Supplementary Figure S7: Specificity of mSA labeling of biotinylated AP-GluA2 in organotypic hippocampal slice cultures

(**A-B**). Organotypic slice cultures from AP-GluA2 KI or WT mice were transduced with AAV9 encoding BirA^{ER}-eGFP or eGFP control in CA1 and maintained for 15 days in media supplemented with 10 µM biotin. Slices were incubated with 400 nM mSA conjugated to STAR 635P to label surface localized bAP-GluA2. (**A**). Representative widefield images of organotypic hippocampal slices transduced in CA1 with BirA^{ER}-eGFP or eGFP control from WT or KI mice. mSA labeling is specific to AP-GluA2 KI with BirA^{ER}. Bottom panel shows a representative confocal image from the boxed region. Scale bars, 200 and 20 µm. (**B**). Schematic representation of the experiment. (**C**). Normalized fluorescence intensity of mSA-STAR 635P labeling in CA1, coincident with the eGFP reporter. N≥6. **-****P*≤0.0072 (Kruskal-Wallis test; F=18.37, *P*=0.0001; Dunn's post-hoc test). (**D**). Normalized fluorescence intensity of eGFP in CA1. N≥6. *P*≥0.8237 (one-way ANOVA; F=0.1976, *P*=0.8222; Tukey's post-hoc test). Error Bars, SEM.

Supplementary Figure S8: Sparse labeling of biotinylated AP-GluA2 with serial dilution of BirA^{ER}-eGFP AAV

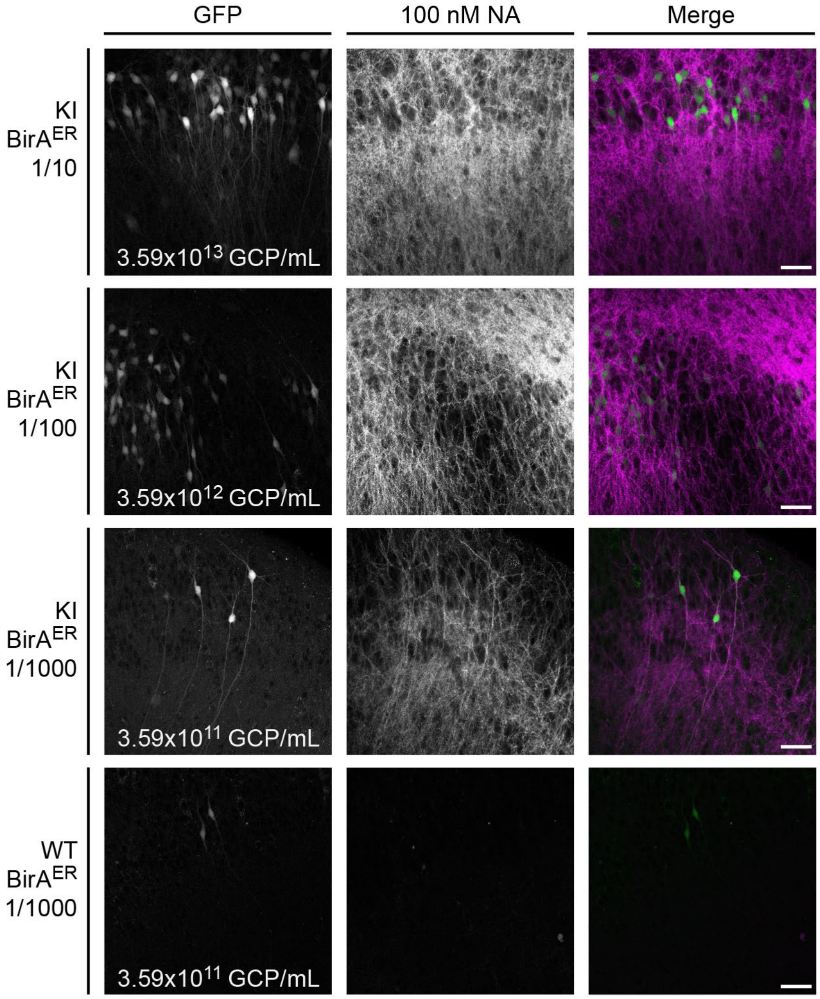

Supplementary Figure S8: Sparse labeling of biotinylated AP-GluA2 with serial dilution of BirA^{ER}-eGFP AAV

Organotypic slice cultures from AP-GluA2 KI or WT mice were transduced using 10-fold serial dilution of the AAV9 encoding BirA^{ER}-eGFP in CA1 and maintained for 15 days in media supplemented with 10 µM biotin. Slices were incubated with 100 nM NA conjugated to STAR 635P to label surface localized bAP-GluA2. N≥3. Representative single-plane confocal images of CA1 pyramidal neurons in KI or WT slices transduced with BirA^{ER}-eGFP. Viral titers are indicated on the left panel. NA labeling of bAP-GluA2 is specific to KI slices within the AAV infection zone, but can be observed below the detectable limit of the eGFP reporter. WT slices exhibit no NA labeling. Scale bars, 50 µm.

Supplementary Figure S9: Photomanipulation with lattice light-sheet microscopy

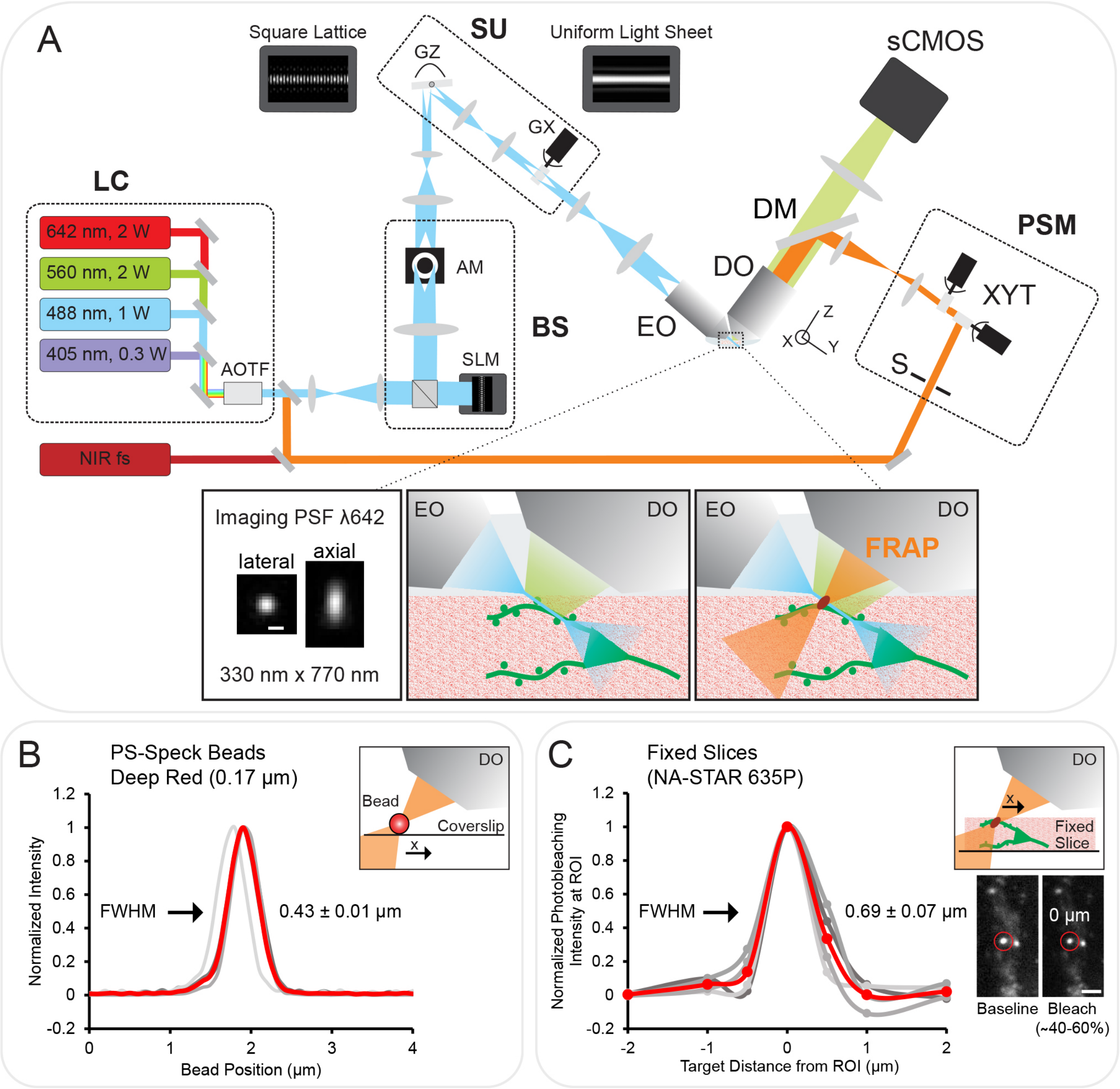

Supplementary Figure S9: Photomanipulation with lattice light-sheet microscopy

(**A**). Lattice light-sheet microscope (LLSM) showing the original Betzig setup (*56*), with the added photostimulation module (PSM). Excitation path is shown in blue, emission path in green, and photomanipulation path in orange. LC: four wavelengths laser combiner; NIR fs: pulsed NIR fs laser for 2-photon photostimulation and uncaging; AOTF: acousto-optic tunable filter; BS: beam shaper, composed of a spatial light modulator (SLM) and an annular mask (AM), generates the square lattice pattern; SU: scanning unit with Z and X galvos (GZ, GX), translates the square lattice pattern along the x and z axis, fast dithering with GX creates a uniform light sheet at the sample; EO: excitation objective; DO: detection objective; sCMOS camera detects fluorescence over the illuminated plane; PSM: photostimulation module, targets user-defined ROIs at high spatiotemporal resolution; S: shutter; XYT: galvo-based beam targeting system. Inserts show imaging point spread function at 642 nm excitation measured as $1/e^2$ radius (left), schematics of the sample with light sheet excitation and detected emission fluorescence (center), and the FRAP beam (right). The light sheet is a thin and extended plane (~ 0.5 µm in z, >15 µm in Y, 100 µm in X) allowing fast 3D high resolution imaging. Scale bar, 0.5 µm. (**B**). Photomanipulation beam dimension was measured by scanning a 0.17 µm diameter PS-Speck deep red bead (633/660 nm) across the beam focus (0.43 ± 0.01 µm full width at half maximum; FWHM). N=4, average profile shown in red. (**C**). FRAP dimension and efficiency in optically aberrating brain tissue was measured in fixed organotypic hippocampal slice cultures from AP-GluA2 KI mice that were transduced with $BirA^{ER}$-Cre + FLEx eGFP AAV9 and incubated with 100 nM NA-STAR 635P. The FRAP ROI was targeted at ± 0, 0.5, 1, and 2 µm from NA-labelled spines to measure the photobleaching profile in response to a ~3 mW, 50 ms pulse at 642 nm (~40-60% photobleach intensity; 0.69 ± 0.07 µm full width at half maximum; FWHM). N=4, average profile shown in red. Scale bar, 2 µm.

Supplementary Figure S10: FRAP recovery profiles do not correlate with AMPAR density

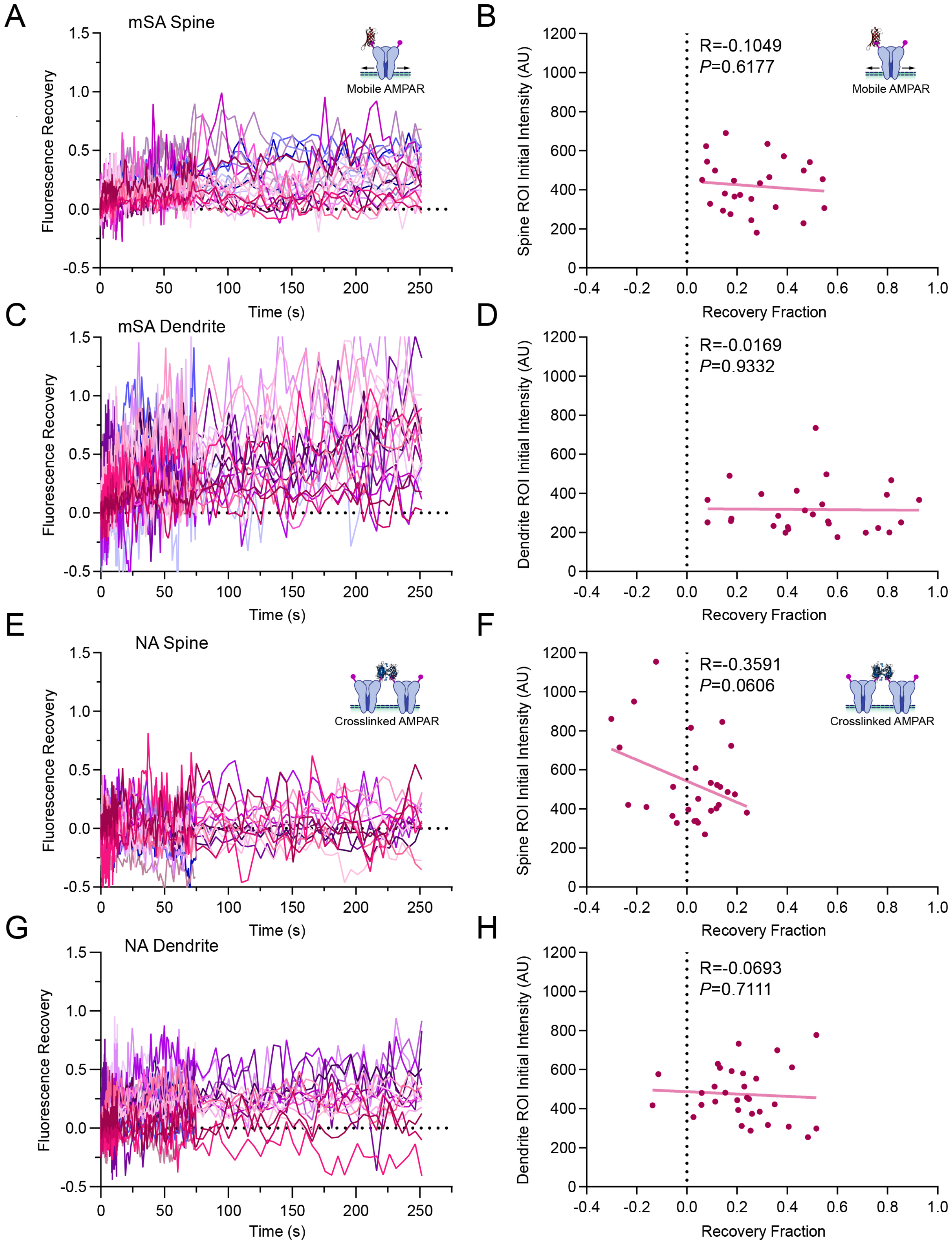

Supplementary Figure S10: FRAP recovery profiles do not correlate with AMPAR density

(**A**-**H**). Left panel: Individual FRAP profiles observed at synaptic or dendritic ROIs, bAP-GluA2 was labelled with 400 nM mSA-STAR 635P (**A**, **C**) or 100 nM NA-STAR 635P (**E**, **G**) in AP-GluA2 KI organotypic slices expressing $BirA^{ER}$ + eGFP. N ≥25. Right panel: No correlation between recovery fraction and AMPAR density was found, as measured by curve fitting of individual FRAP profiles to estimate AMPAR mobile fraction and by initial synaptic or dendritic ROI intensity of mSA-STAR 635P (**B**, **D**) or NA-STAR 635P (**F**, **H**). $P \geq 0.0606$. (Pearson R=-0.1049 P=0.6177, R=0.9332 P=0.9332, R=-0.3591 P=0.0606, R=-0.0693 P=0.7111).

Supplementary Figure S11: AP-GluA2 dendritic trafficking reveals extent of AMPAR internalization with monovalent or tetravalent biotin binding proteins

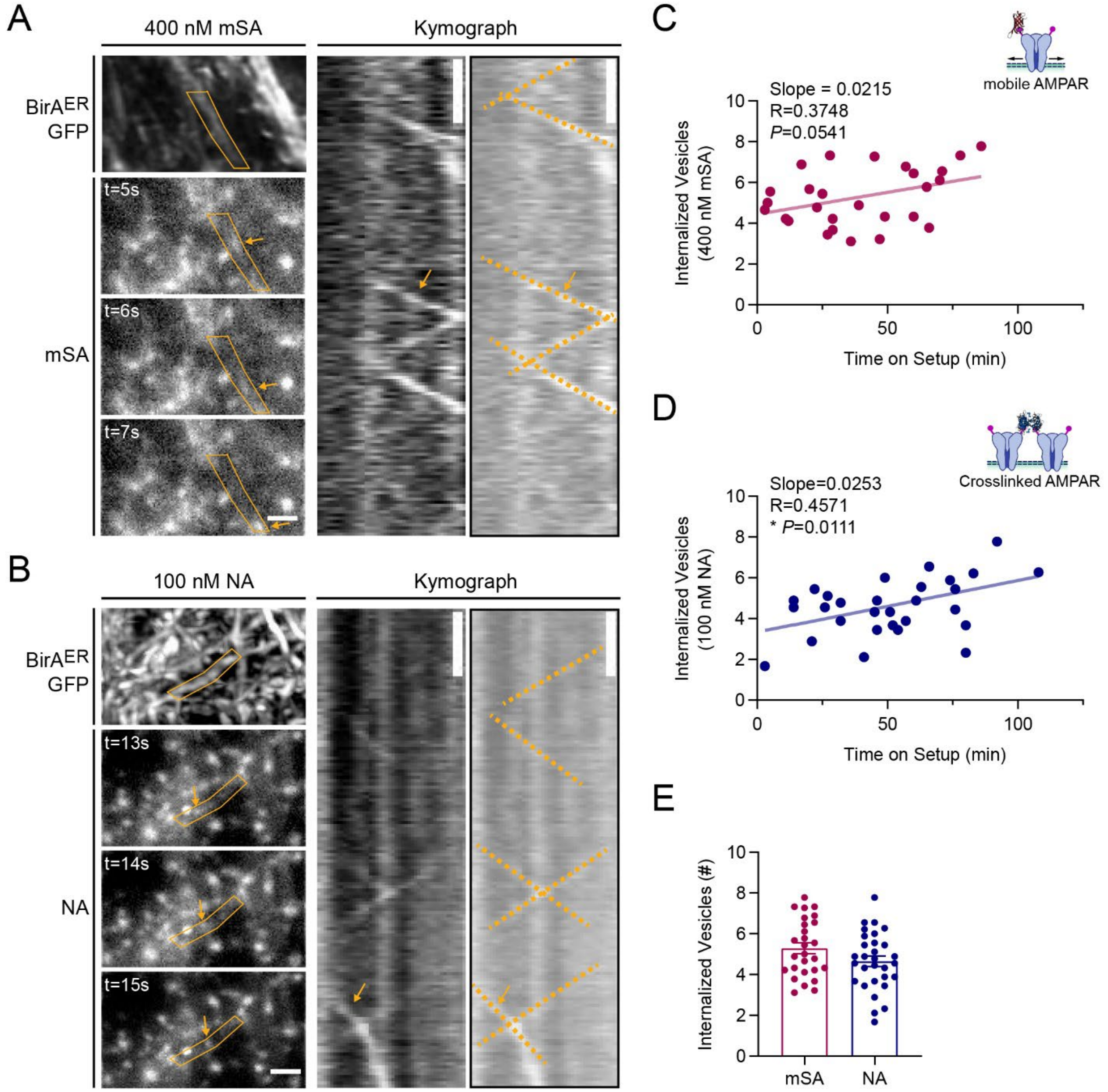

Supplementary Figure S11: AP-GluA2 dendritic trafficking reveals extent of AMPAR internalization with monovalent or tetravalent biotin binding proteins

(**A-B**). Representative LLSM images of CA1 pyramidal neurons in organotypic hippocampal slice cultures from AP-GluA2 KI mice transduced with AAV9 encoding $BirA^{ER}$-eGFP in CA1 and maintained for 12-15 days in media supplemented with 10 µM biotin. Slices were incubated with 400 nM mSA-STAR 635P (**A**) or 100 nM NA-STAR 635P (**B**) to label surface localized biotinylated AP-GluA2. Live 3D volume stack of the eGFP reporter (top left) and continuous single plane acquisitions (10 Hz) of mSA or NA reveal intracellular trafficking events of AMPAR-containing internalized vesicles (arrows), quantified with kymographs along ~5 µm of dendrite (right). Scale bars, 2 µm and 2 s. (**C-D**). Correlation of internalized vesicle counts vs time on setup for 400 nM mSA-STAR 635P (**C**; Pearson R=0.3748, *P*=0.0541) or 100 nM NA-STAR 635P (**D**; Pearson R=0.4571, **P*=0.0111) labeled slices. (**E**). Mean number of internalized vesicles detected. N≥27. *P*=0.0944 (unpaired T-test). Error Bars, SEM.

Supplementary Figure S12: TEV proteolytic cleavage of surface AP-GluA2 reveals extent of AMPAR internalization with monovalent or tetravalent biotin binding proteins

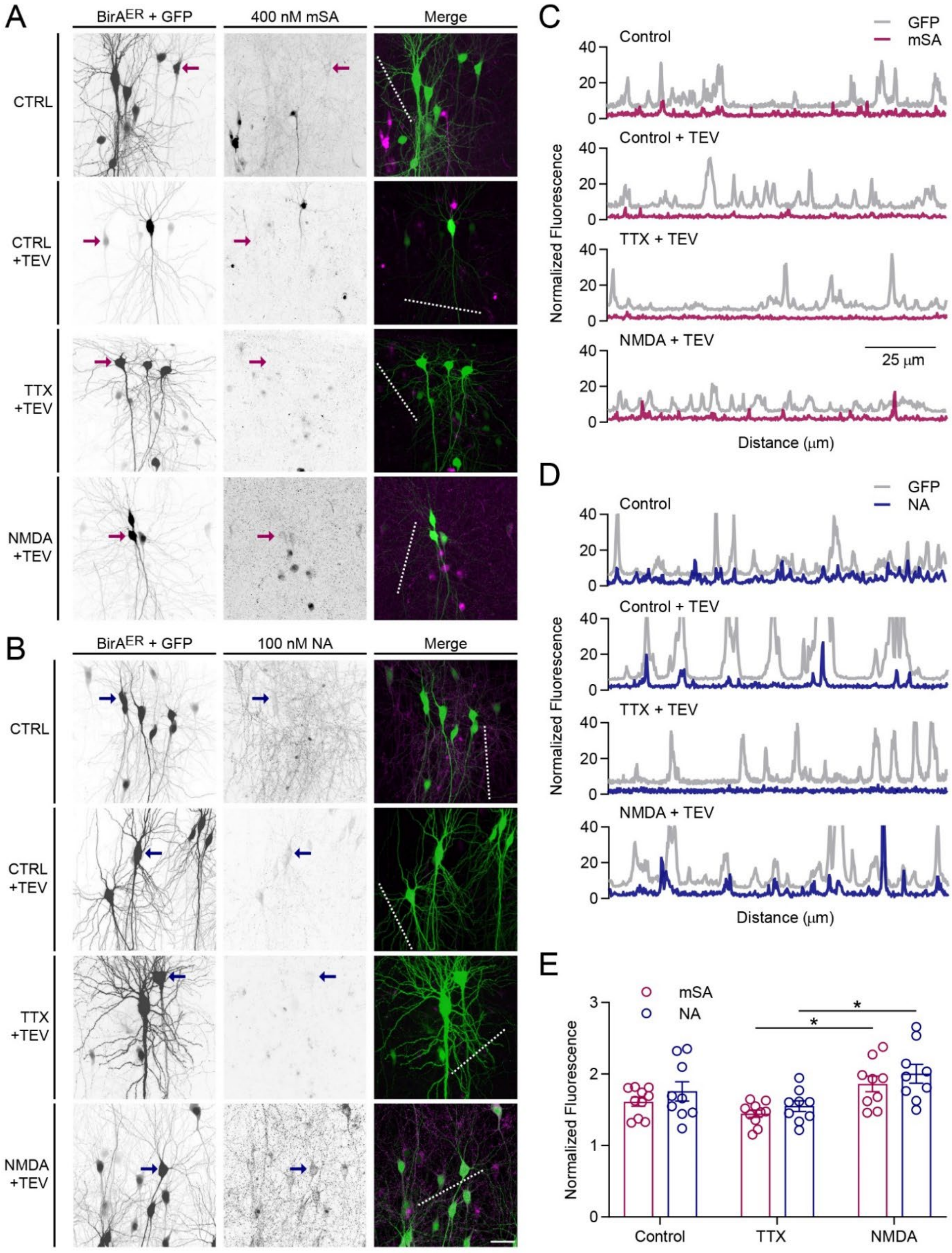

Supplementary Figure S12: TEV proteolytic cleavage of surface AP-GluA2 reveals extent of AMPAR internalization with monovalent or tetravalent biotin binding proteins

(**A-B**). Representative confocal images of CA1 pyramidal neurons in organotypic slice cultures from AP-GluA2 KI mice transduced with AAV9 encoding $BirA^{ER}$-Cre + FLEx eGFP in CA1 and maintained for 12-15 days in media supplemented with 10 µM biotin. Slices were incubated with 400 nM mSA-STAR 635P (**A**) or 100 nM NA-DyLight 633 (**B**) to label surface localized bAP-GluA2, then incubated for 30 min in control conditions, with TTX (1 µM), or after NMDA treatment (30 µM, 3 min). Slices were then incubated with a control buffer or 100 U TEV protease (10 min) to cleave the AP tag and remove the mSA or NA surface label to reveal internalized AMPAR. Scale bar, 20 µm. (**C-D**). Linescans (dashed lines in A, B) reveal colocalization of mSA (**C**) or NA (**D**) with the eGFP reporter, and differential AMPAR internalization upon TTX or NMDA treatment (+TEV). (**E**). Normalized fluorescence intensity of mSA-STAR 635P or NA-DyLight 633 after TEV proteolytic cleavage, coincident with the eGFP reporter. Crosslinking by tetravalent NA does not alter AMPAR surface distribution or recycling. $N \geq 9$. $*P \leq 0.0400$ (one-way ANOVA; $F=4.5940$, $P=0.0016$; Tukey's post-hoc test). Error Bars, SEM.

Supplementary Figure S13: Sample preparation for TEM

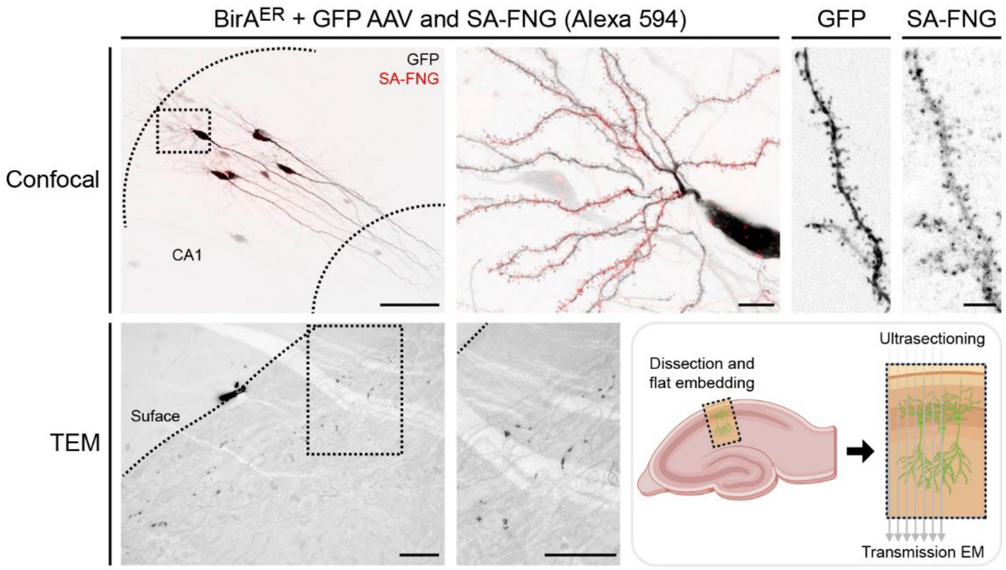

Supplementary Figure S13: Sample preparation for TEM

Representative confocal images of organotypic hippocampal slice cultures from AP-GluA2 KI mice transduced with AAV9 encoding BirA^{ER}-Cre + FLEx eGFP in CA1 and maintained for 15 days in media supplemented with 10 µM biotin, then incubated for 20 min with 100 nM StreptAvidin conjugated to AlexaFluor 594 FluoroNanoGold (SA-FNG) and fixed. Scale bars, 100, 10 and 5 µm (top panel). SA-FNG labeled slices were then processed and embedded for ultrasectioning of eGFP-positive ROIs. A low magnification image in TEM shows nanogold particle labeling after pre-embedding silver enhancement. Scale bars, 1 µm (bottom panel). Schematic representation of the experiment is shown on the bottom right. TEM synaptic micrographs are shown in Figure 4K, and quantification is shown in Figure 4L.

Supplementary Figure S14: Characterization of AP-GluA2 and synaptic protein expression in KI and WT brain

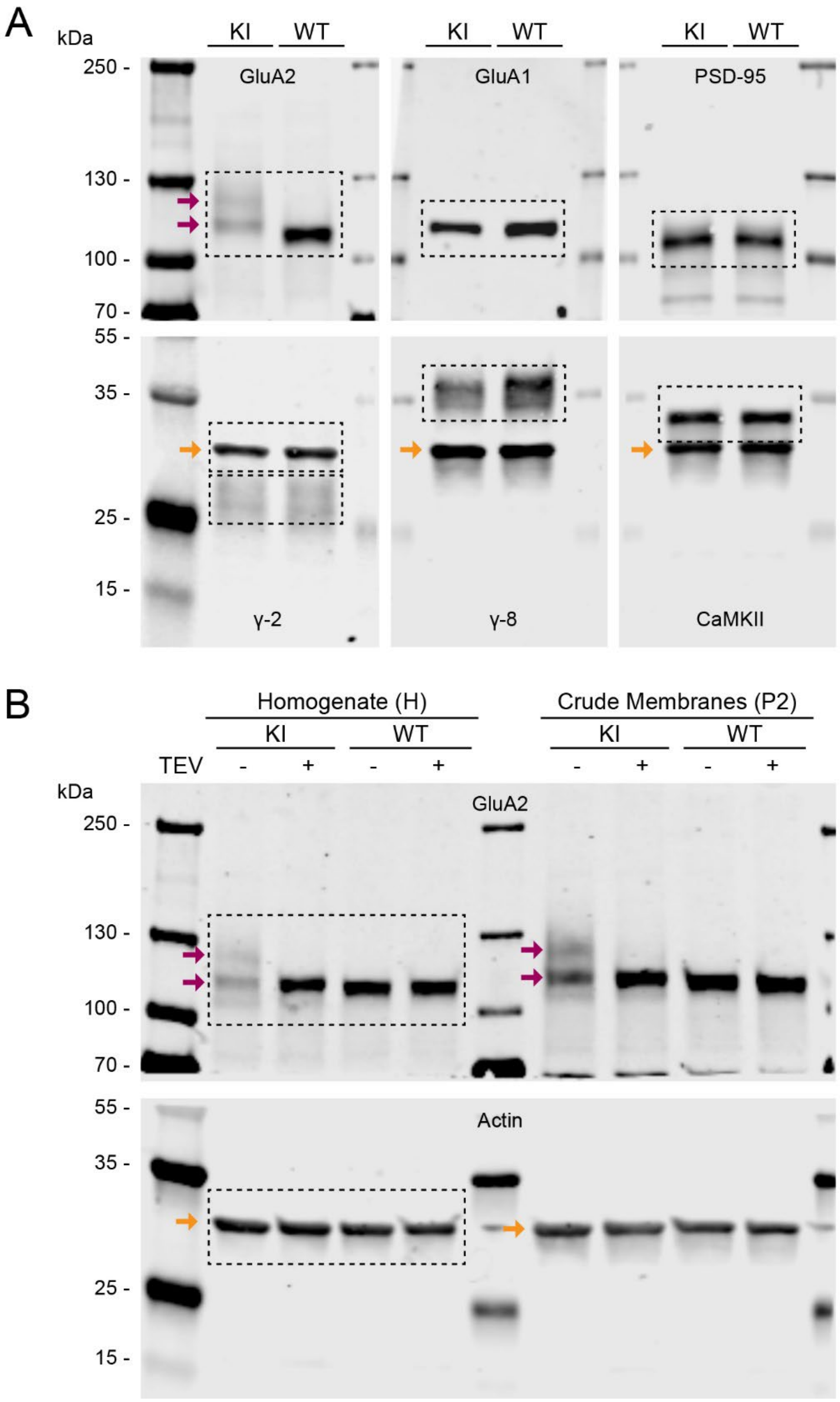

Supplementary Figure S14: Characterization of AP-GluA2 and synaptic protein expression in KI and WT brain

(**A**). Representative Western blots of synaptic proteins in AP-GluA2 KI and WT whole brain protein samples. Double banding of GluA2 is observed for AP-GluA2 KI (red arrows). Boxed regions are shown in Figure 5A, and quantification is shown in Figure 5B. (**B**). Representative Western blot of the TEV proteolytic cleavage assay with whole brain lysate (H) or crude membrane fractions (P2) from KI and WT mice. Double banding of GluA2 is observed for AP-GluA2 KI without TEV (red arrows), and a single band with the same relative expression as WT is observed upon incubation with TEV protease. Boxed regions are shown in Figure 5C, and quantification is shown in Figure 5D. Membranes were cut between the 70 and 55 kDa markers to facilitate differential antibody labeling. β-actin was used as a loading control (orange arrows).

Supplementary Figure S15: N-terminal degradation underlies the double banding pattern of AP-GluA2

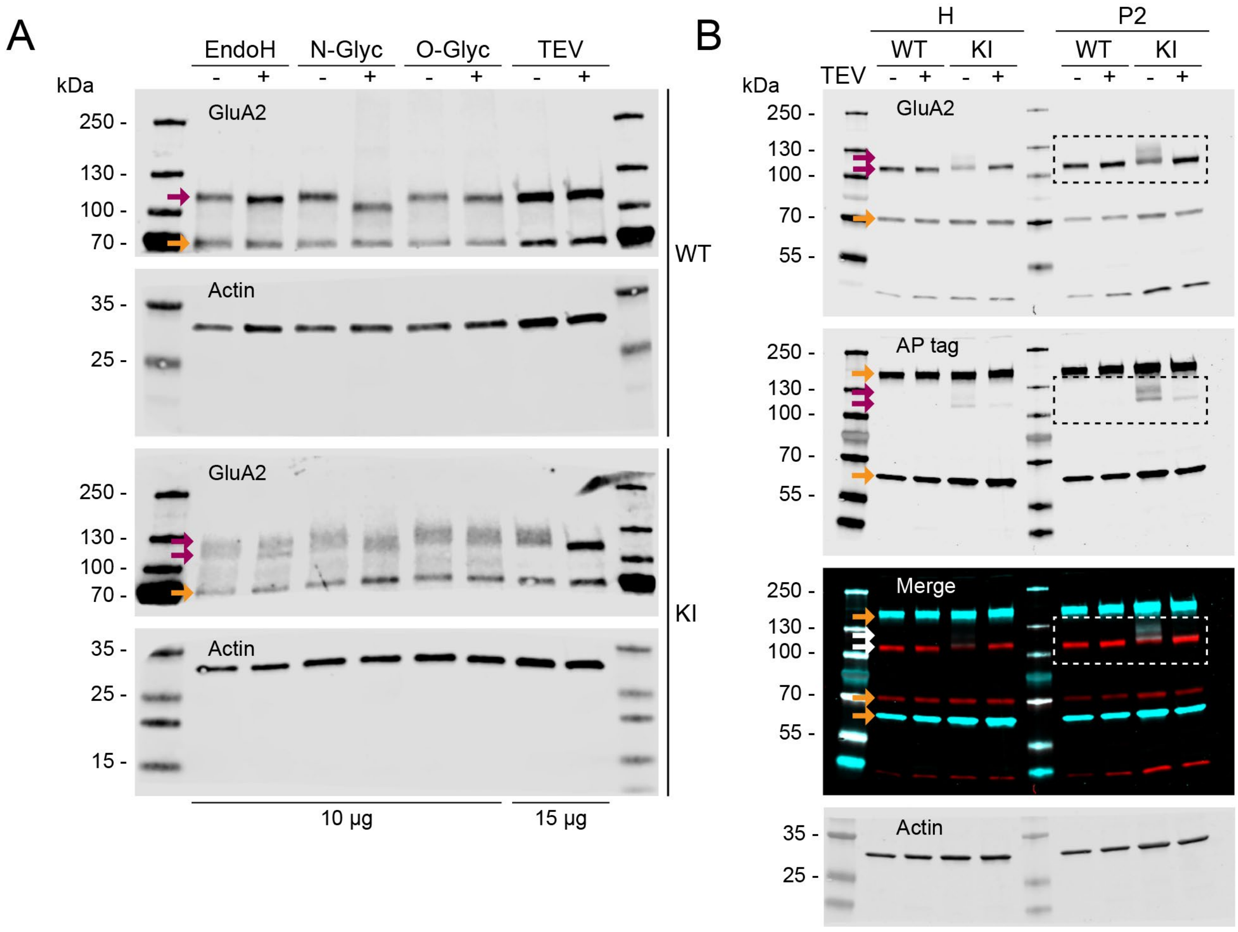

Supplementary Figure S15: N-terminal degradation underlies the double banding pattern of AP-GluA2

(**A**). Representative Western blots of the GluA2 deglycosylation assay in WT and KI mice, protein samples were incubated with or without EndoH (N-glycan), PNGase F (N-Glyc; N-glycan), O-Glycosidase (O-Glyc; O-glycan), or TEV protease. Double banding of GluA2 is observed for AP-GluA2 KI (red arrows). Deglycosylation of GluA2 increases migration but does not affect double banding pattern of AP-GluA2. (**B**). Representative Western blots of AP tag and GluA2 (red arrows) in whole brain lysate (H) or crude membrane fractions (P2) from WT and KI mice, incubated with or without TEV protease. Boxed regions are shown in Figure 5E. Orange arrows indicate aspecific bands of the GluA2 or AP tag antibody. Membranes were cut between the 70 and 55 kDa markers to facilitate differential antibody labeling. β-actin was used as a loading control.

Supplementary Figure S16: Biochemical characterization of AP-GluA2 in vitro biotinylation

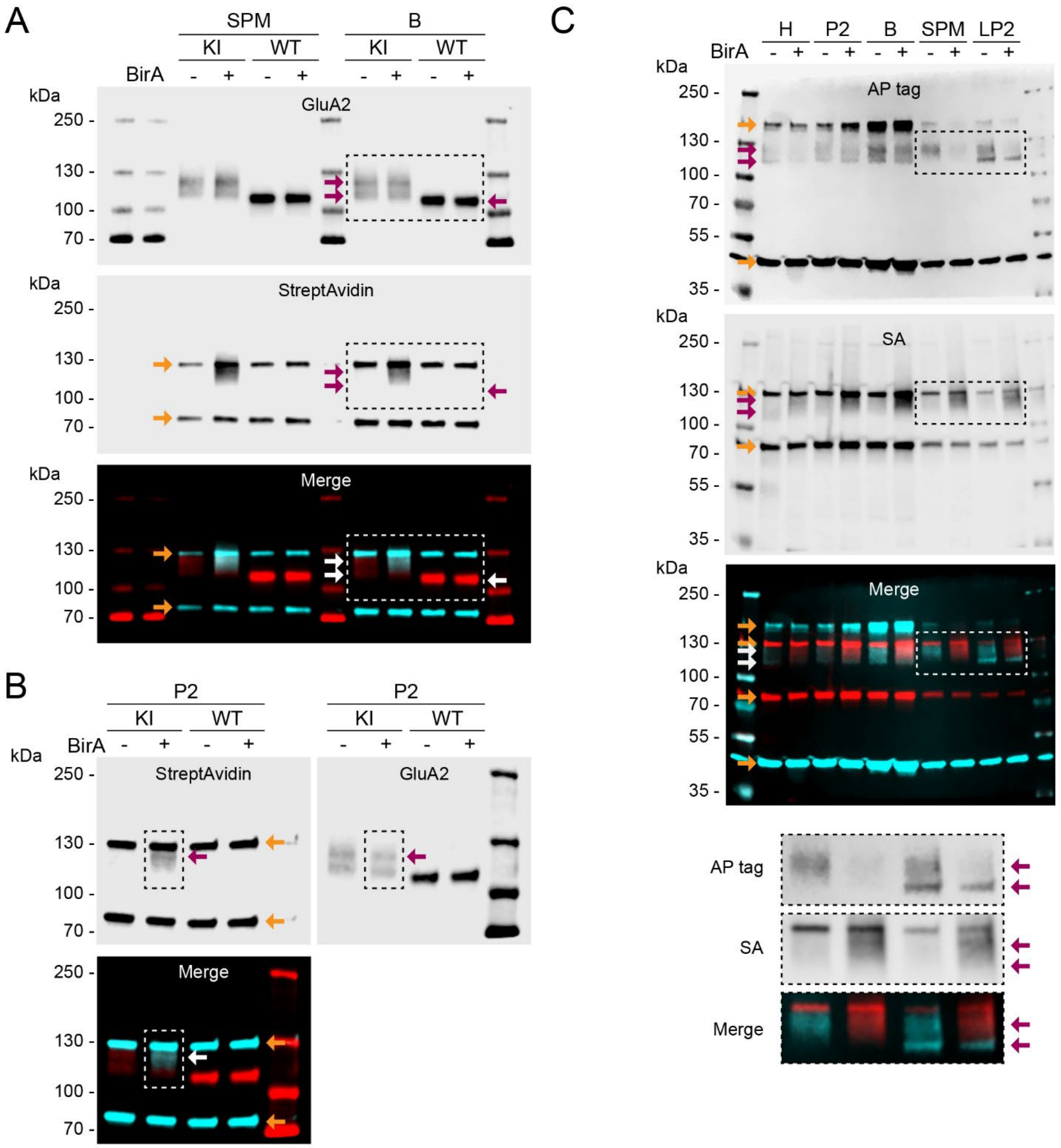

Supplementary Figure S16: Biochemical characterization of AP-GluA2 in vitro biotinylation

(**A**). Representative Western blot of GluA2 in AP-GluA2 KI and WT synaptic plasma membrane (SPM) or synaptosome (B) fractions, incubated with or without soluble recombinant BirA. Double banding of GluA2 is observed for AP-GluA2 KI (red arrows). StreptAvidin binding of biotinylated AP-GluA2 is observed on the upper band. Orange arrows indicate StreptAvidin binding to endogenous biotin binding proteins pyruvate carboxylase (upper) and β-methyl crotonyl CoA carboxylase (lower). β-methyl crotonyl CoA carboxylase was used as a loading control. Boxed regions are shown in Figure 5G. Quantification of SA binding (+ relative to - sBirA), normalized to β-methyl crotonyl CoA carboxylase loading control, is shown in Figure 5K. (**B**). Representative Western blot of GluA2 and SA in crude membrane fractions (P2), as in (A), boxed regions used for linescan analysis are shown in Figure 5H. (**C**). Representative Western blot of AP tag in whole brain lysate (H), crude membrane fractions (P2), synaptosome (B), synaptic plasma membrane (SPM), or intracellular vesicle (LP2) fractions from AP-GluA2 KI mice, incubated with or without soluble recombinant BirA. Double banding of GluA2 is observed with the AP tag antibody (red arrows). StreptAvidin binding of biotinylated AP-GluA2 is observed on the upper band. Orange arrows indicate aspecific bands of the AP tag antibody or StreptAvidin binding to endogenous biotin binding proteins.

Supplementary Figure S17: Subcellular fractionation reveals differential trafficking of AP-GluA2 protein isoforms

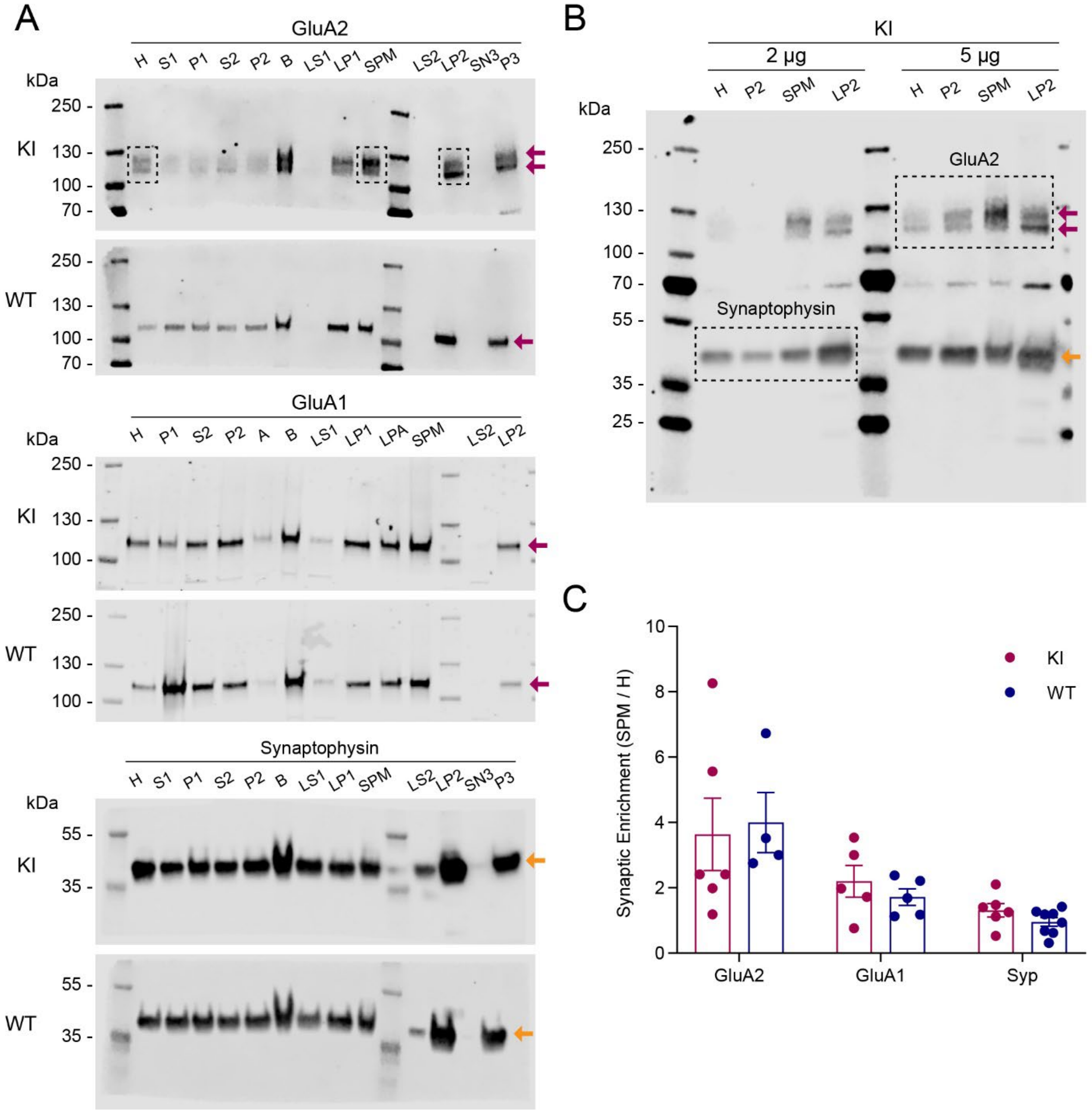

Supplementary Figure S17: Subcellular fractionation reveals differential trafficking of AP-GluA2 protein isoforms

(**A**). Representative Western blots of GluA2, GluA1, and Synaptophysin in AP-GluA2 KI and WT subcellular fractions (red or orange arrows), as described in (*82*). Boxed regions used for linescan analysis are shown in Figure 5J, and quantification is shown in Figure 5L (H, homogenate; P2, crude synaptosomes, used to isolate SPM and LP2 fractions; SPM, synaptic plasma membrane; LP2; crude synaptic vesicles). Synaptophysin was used as a marker of intracellular vesicles, and is enriched in LP2 (orange arrows). (**B**). Representative Western blot of GluA2 (red arrows) and Synaptophysin (orange arrow) in AP-GluA2 KI protein fractions of interest. Boxed regions are shown in Figure 5I. The upper GluA2 band (full length AP-GluA2, biotinylated) is enriched in the SPM fraction. The lower GluA2 band (AP-GluA2 degradation product, not biotinylated) is enriched in the LP2 fraction. (**C**). Quantification of the synaptic enrichment factor for GluA2, GluA1 and Synaptophysin (Syp) from Western blots of subcellular fractionation experiments. Band intensity in synaptic plasma membrane fraction (SPM) was divided by band intensity in total homogenate (H). $N \geq 4$. $P \geq 0.1582$ (unpaired T-tests). Error Bars, SEM.

Supplementary Figure S18: Secondary-only control for GluA2 immunohistochemistry

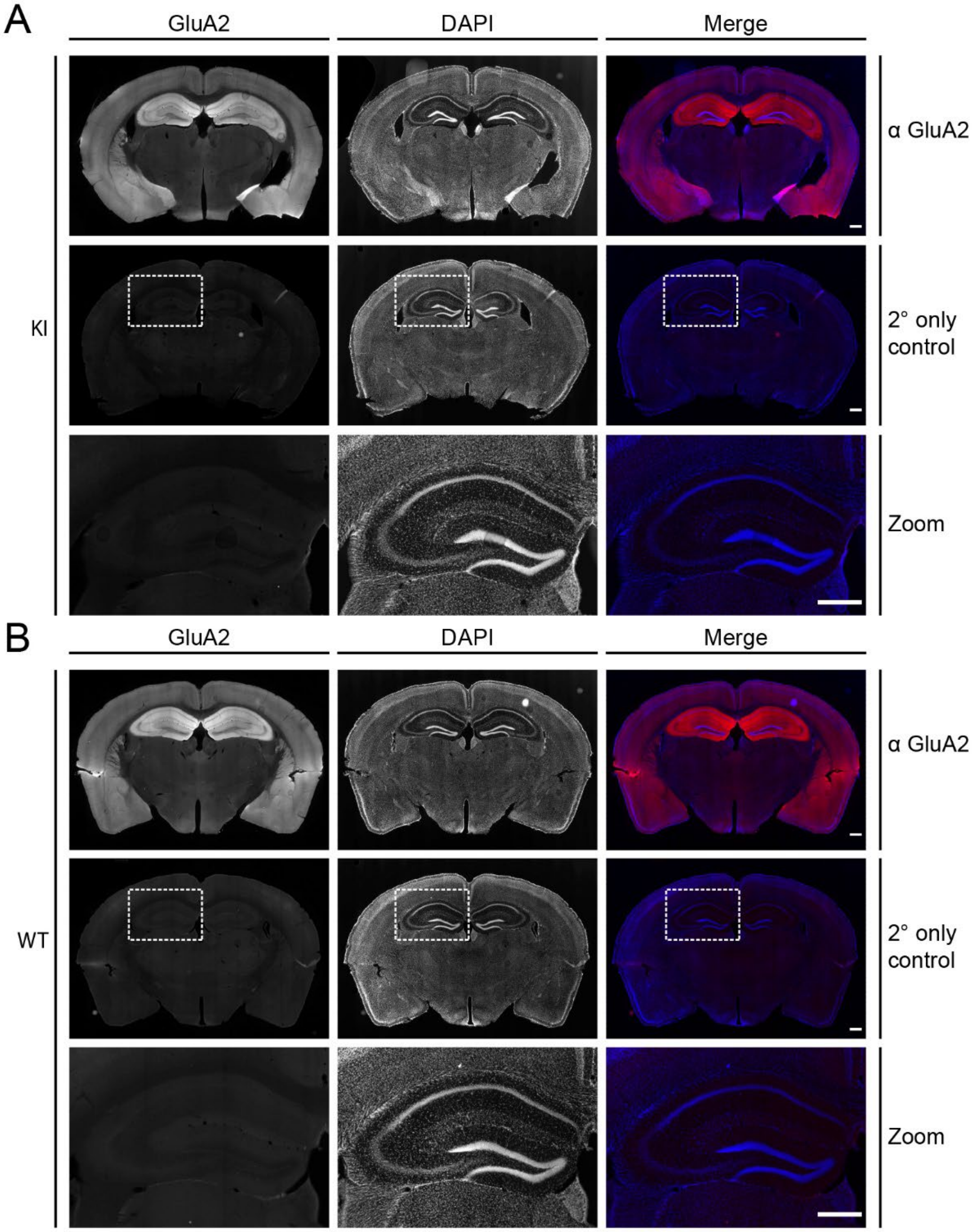

Supplementary Figure S18: Secondary-only control for GluA2 immunohistochemistry

(**A-B**). Characterization of GluA2 protein expression in frontal sections from AP-GluA2 KI (**A**) or WT mice (**B**), incubation without GluA2 primary antibody is shown to validate specificity of the fluorescent signal. Serial sections incubated with or without GluA2 primary antibody were used to normalize the fluorescence intensity signal for the data reported in Figure 6E. Scale bars, 500 µm.

Supplementary Figure S19: Synaptic network characterization in acute slices from AP-GluA2 KI mice

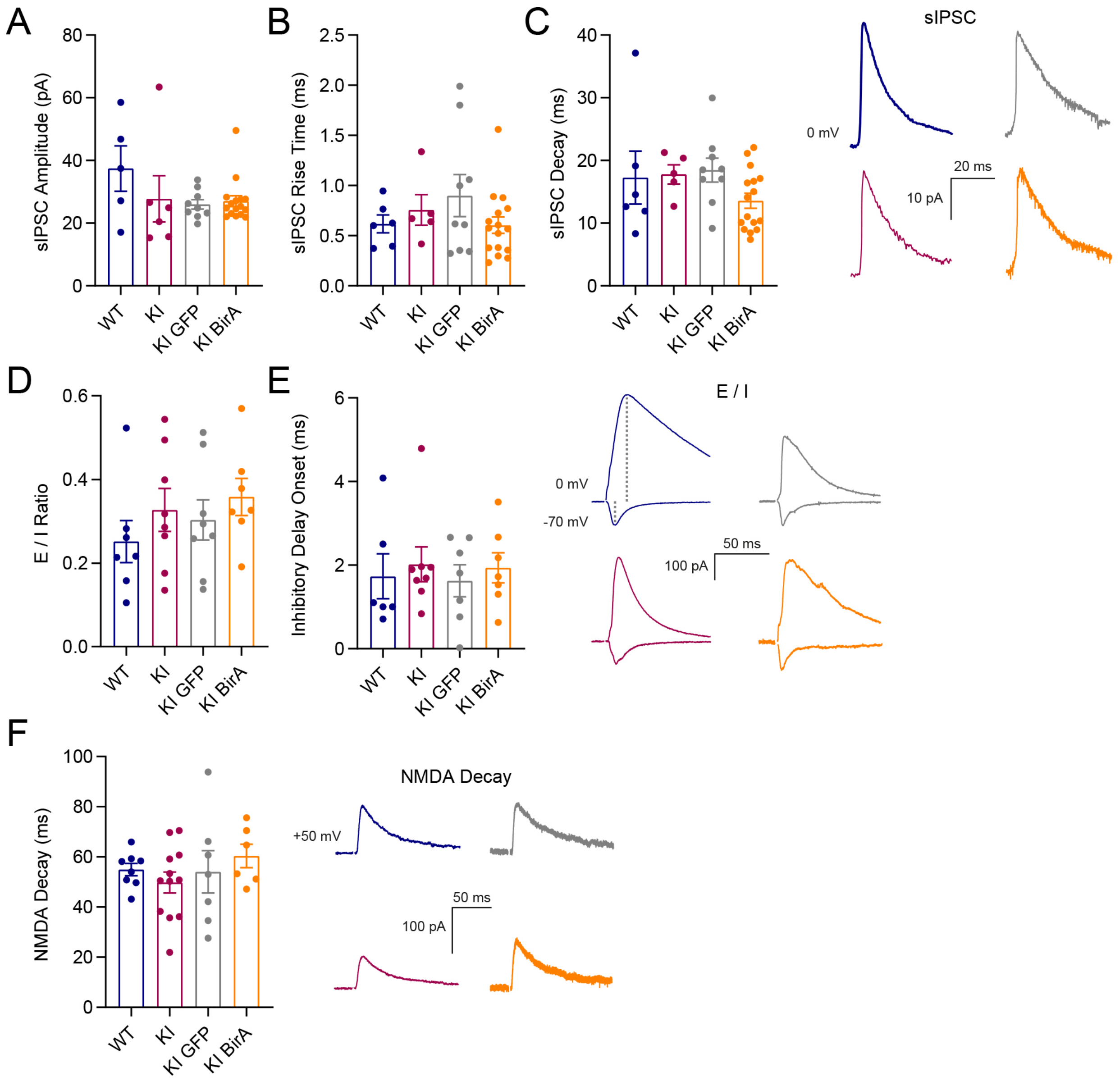

Supplementary Figure S19: Synaptic network characterization in acute slices from AP-GluA2 KI mice

(**A-F**). Characterization of synaptic physiology at CA3-CA1 Schaeffer collaterals in acute slices from WT, KI, KI + eGFP or KI + $BirA^{ER}$ experimental groups using whole-cell voltage-clamp electrophysiological recordings. Representative current responses in CA1 pyramidal neurons and holding potentials are shown on the right (WT in blue, KI in red, KI + eGFP AAV in grey, KI + $BirA^{ER}$-eGFP AAV in orange). (**A-C**). Characterization of the amplitude (**A**), rise time (**B**) and decay time (**C**) of spontaneous inhibitory postsynaptic currents (sIPSC). N≥5. $P \geq 0.2396$ (Kruskal-Wallis test or one-way ANOVA; F=3.282 P=0.3516, F=2.039 P=0.5644, F=1.560 P=0.2183; Dunn's or Tukey's post-hoc test). (**D**-**E**). Feed-forward inhibition evoked by stimulation of Schaeffer collaterals was used to assess the excitation-inhibition balance (E/I) of hippocampal circuits (**D**) and delay of inhibitory current onset (**E**). N≥7. $P \geq 0.4555$ (one-way ANOVA or Kruskal-Wallis test; F=0.8011 P=0.5045, F=0.8110 P=0.8468; Tukey's or Dunn's post-hoc test). (**F**). NMDA current decay time was used to assess synaptic composition of NMDA receptors. N≥6. $P \geq 0.4882$ (one-way ANOVA; F=0.7097, P=0.5541; Tukey's post-hoc test). Error Bars, SEM.

Supplementary Figure S20: Basal transmission controls for field recordings in acute slices

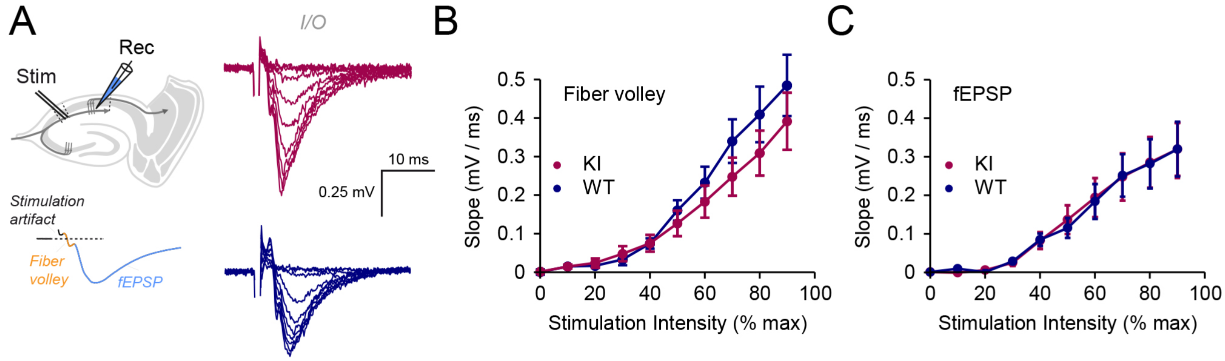

Supplementary Figure S20: Basal transmission controls for field recordings in acute slices

(**A**). Functional impact of AP-GluA2 KI was assessed using extracellular field recordings of presynaptic fiber volleys (FV) and excitatory postsynaptic potentials (fEPSP) resulting from Schaeffer collateral stimulations of increasing intensity. Representative responses for stimulations between 10-90% of maximal intensity are shown on the right. (**B-C**). Input/output (I/O) curves of FV (**B**) and fEPSP (**C**) responses to stimulations of increasing intensity recorded in the CA1 region of acute slices from adult KI and WT mice. N≥14. *P*≥0.0539 (unpaired T-test or Mann-Whitney U-test). Error bars, SEM.

Supplementary Figure S21: SHIRPA behavioral assessment of AP-GluA2 KI mice

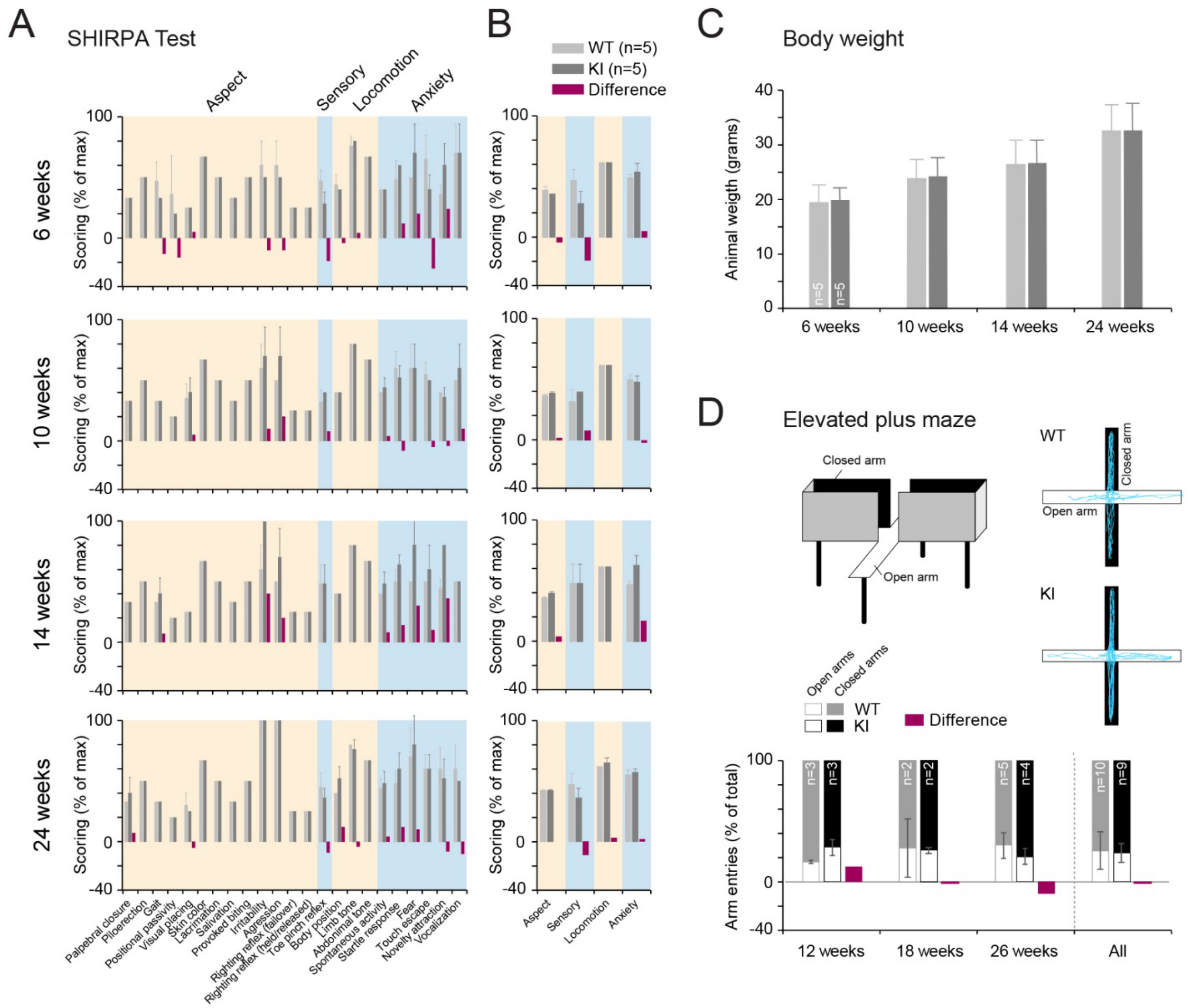

Supplementary Figure S21: SHIRPA behavioral assessment of AP-GluA2 KI mice

(**A-B**). Behavioral assessment protocol for phenotype characterization of the AP-GluA2 KI mouse line. SHIRPA (SmithKline Beecham, Harwell, Imperial College, Royal London Hospital, phenotype assessment) longitudinal analysis of WT and KI mice at 4 time points (6, 10, 14 and 24 weeks). n=5. (**A**). Detailed screening behavior analysis for general aspect investigation, anxiety, locomotion and sensory abnormalities. Red bars represent the difference ratio between the two groups. (**B**). Grouped analysis for each aspect, Aspect-Sensory-Locomotion-Anxiety. Red bars represent the difference ratio between the two groups. (**C**). Body weight of WT and KI mice at 4 time points (6, 10, 14 and 24 weeks). $P \geq 0.8781$ (unpaired T-tests). (**D**). As the SHIRPA results suggested a possible effect on anxiety levels of AP-GluA2 KI mice at 14 weeks of age, this scoring was complemented by conducting the elevated plus maze test in adult WT and KI mice. The elevated plus maze apparatus consisted of two opposed open and enclosed arms (50×10×40 cm LWH) and an open central square (10×10 cm LW), elevated 50 cm above the floor. At the beginning of each trial, the mouse was placed at the end of the closed arm. Then animal displacement was assessed for 10 consecutive min using EthoVision XT (Noldus). Schematic representation of the elevated plus maze test (top left). Deep lab cut tracking profile analysis of WT and KI mice in open and closed arms of the maze are shown in blue (top right). Percentage of open (white) and closed (shaded) arm entries observed in WT and KI mice at 3 time points (12, 18 and 26 weeks). Red bars represent the difference ratio between the two groups. $P=0.8246$ (unpaired T-test; all data). Error Bars, SEM.

Supplementary Figure S22: AMPAR immobilization by NA crosslinking does not affect US-evoked freezing increase during acquisition of cued fear memory

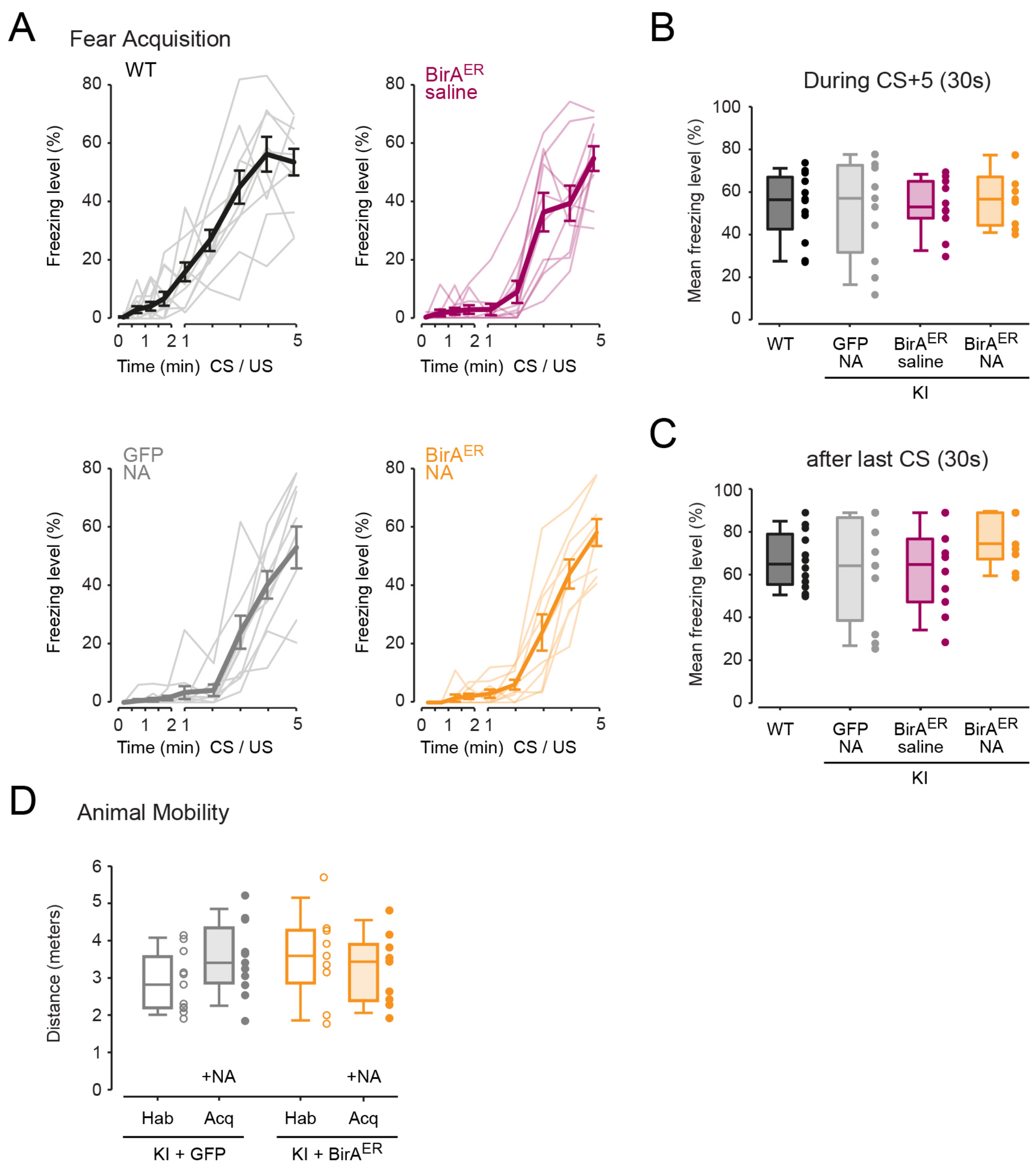

Supplementary Figure S22: AMPAR immobilization by NA crosslinking does not affect US-evoked freezing increase during acquisition of cued fear memory

(**A**). Effect of $BirA^{ER}$ expression and/or NA application on animal sensitivity to noxious stimuli was tested by comparing the freezing levels observed along CS/US applications. As expected, upon US deliveries (timely associated with CS+ sounds), fear reaction increased. This effect was similar between groups. (**B**). Comparison of the mean freezing levels observed during CS5 presentation (30 s, terminating with US delivery). N≥9. P≥0.8309 (one-way ANOVA; F=0.1718, P=0.9148; Dunnett's post-hoc test). (**C**). Comparison of the mean freezing levels observed after last US delivery (30 s following US delivery). N≥9. P≥0.2200 (one-way ANOVA; F=1.258, P=0.3024; Dunnett's post-hoc test). (**D**). To control for the effect of NA-mediated AMPAR crosslinking on total locomotion, we compared total distance made by $BirA^{ER}$-eGFP or eGFP control AAV injected animals during 2 minutes in habituation (Hab; before NA infusion) and immediately before CS/US pairs, 10-15 minutes after Na infusion (Acq), in context A. N≥9. P≥0.2496 (two-way ANOVA; F=2.364, P=0.1416; Sidak's multiple comparisons test). Error Bars, SEM.

KEY RESOURCES TABLE

Reagent or Resource	Source	Identifier
**Antibodies**		
GluA2 (15F1), mouse monoclonal IgG1κ	Provided by E. Gouaux (*83*)	Clone 15F1
GluA2	Millipore	Cat # MAB397
GluA2	Alomone	Cat # AGC-005
GluA1	Neuromab	Cat # 75-327
PSD-95	Neuromab	Cat # 75-028
Gamma 8	Frontier Institute	Cat # TARPgamma8-RbAF1000
Stargazin	CST	Cat # 8511
CaMKII	Millipore	Cat # 05-532
β-Actin	Sigma-Aldrich	Cat # 5316
Synaptophysin	Abcam	Cat # 32127
AP tag	Kerafast	Cat # EGO016
Goat anti-mouse Alexa 568	ThemoFisher	Cat # A-21124
Goat anti-mouse IRDye680LT	LiCor	Cat # 926-68020
Goat anti-rabbit IRDye680LT	LiCor	Cat # 926-68021
Goat anti-rabbit IRDye800CW	LiCor	Cat # 926-32211
**Biochemical Reagents**		
AcTEV	Invitrogen	Cat # 12575-015
Biotin-AMP	Jena Bioscience	Cat # NU-894-BIO-S
Recombinant BirA	Sigma-Aldrich	Cat # SRP0417
MNI-caged-L-glutamate	Hello Bio	Cat # HB0423
NMDA	Sigma-Aldrich	Cat # M3262
TTX	Tocris	Cat # 1069
Picrotoxin	Sigma-Aldrich	Cat # P1675
Spermine	Tocris	Cat # 0958
D-AP5	Tocris	Cat # 0106
endoH	NEB	Cat # P0702S
PNGase F	NEB	Cat # P0704S
O-Glycosidase	NEB	Cat # P0733S
Calbiochem protease inhibitor	Millipore	Cat # 539134
Halt phosphatase inhibitor	ThermoFisher	Cat # 78420
Pierce BCA assay	ThermoFisher	Cat # 23225
PageRuler plus prestained protein ladder	ThermoFisher	Cat # 26619
**Experimental Models**		
Mouse: C57BL/6J	Charles River / JAX®	Strain # 000664 https://www.jax.org/strain/000664

Mouse: AP-GluA2 KI or WT B6J-Gria2$^{em1(AP-TEV)Ics/Iins}$ N2	Custom, PHENOMIN Mouse Clinical Institute	N/A
**Molecular Probes**		
Monomeric StreptAvidin (mSA)	Custom (*30*)	N/A
Unconjugated NeutrAvidin (NA)	ThermoFisher	Cat # 31000
STAR 635P	Abberior	Cat # ST635P
NA-STAR 635P	Abberior	Cat # ST635P-0121
NA-DyLight 550	Invitrogen	Cat # 84606
NA-DyLight 633	Invitrogen	Cat # 22844
NA-Texas Red	Invitrogen	Cat # A2665
StreptAvidin FluoroNanoGold AlexaFluor 546 (SA-FNG)	Nanoprobes Inc.	Cat # 7416
StreptAvidin FluoroNanoGold AlexaFluor 594 (SA-FNG)	Nanoprobes Inc.	Cat # 7316
StreptAvidin IRDye800CW	LiCor	Cat # 926-32230
**Recombinant DNA and Viruses**		
pDisplay: IgK leader HA BirA^{ER}	Gift from A. Ting (*28*)	Addgene; Cat # 20856
pAAV: CAG IgK leader HA BirA^{ER} IRES eGFP	Custom	N/A
pAAV: hSyn IgK leader HA BirA^{ER} IRES eGFP	Custom	N/A
pAAV: hSyn IgK leader HA BirA^{ER} IRES Cre	Custom	N/A
pAAV: CAG IRES eGFP	Custom	N/A
pAAV: hSyn IRES eGFP	Custom	N/A
AAV1/9: hSyn IgK leader HA BirA^{ER} IRES eGFP	Custom	N/A
AAV1/9: hSyn IRES eGFP	Custom	N/A
AAV9: hSyn IgK leader HA BirA^{ER} IRES Cre	Custom	N/A
AAV9: SynP DIO eGFP	Gift from I. Wickersham	Addgene; Cat # 100043
AAV9: hSyn Cre WPRE hGH	Gift from J. Wilson	Addgene; Cat # 105553
AAV9: Syn FLEx GCaMP6f WPRE SV40	Gift from D. Kim & GENIE Project (*76*)	Addgene; Cat # 100833
**Software**		
Clampex 10.7<br>Clampfit 10.7	Molecular Devices, LLC	https://www.moleculardevices.com/products/axon-patch-clamp-

		system/acquisition-and-analysis-software/pclamp-software-suite
Igor Pro 8.04 64-bit	Wavemetrics	https://www.wavemetrics.com/products/igorpro
NeuroMatic v3.0c	Written by J. Rothman (*87*)	http://www.neuromatic.thinkrandom.com/
ANY-maze 6.3 video tracking	Stoelting Co	https://www.any-maze.com/
Fiji ImageJ 1.53c or 1.53f51	NIH	https://imagej.net/Fiji
MetaMorph	Molecular Devices	https://www.moleculardevices.com/products/cellular-imaging-systems/acquisition-and-analysis-software/metamorph-microscopy
WaveTracer, PALM-Tracer	Custom (*86*)	https://imagxcell.com/wave-tracer/
Imaris 9.8.2	Oxford Instruments	https://imaris.oxinst.com/
LabVIEW	National Instruments	https://www.ni.com/fr-fr/shop/labview.html
Lattice Scope	Written by D. Milkie, Betzig team, Janelia Research Campus, HHMI	Custom LLSM acquisition software written in LabVIEW
LLSpy V0.4.8	Written by T. Lambert, Harvard Medical School	https://github.com/tlambert03/LLSpy
Prism 8.3.1 and 9.1.1	GraphPad	https://www.graphpad.com/scientific-software/prism/
SigmaPlot V14	Systat Software Inc	https://systatsoftware.com/products/sigmaplot/
Adobe Illustrator 22.0.1	Adobe	www.adobe.com
